# Experimentally evolving *Drosophila erecta* populations may fail to establish an effective piRNA-based host defense against invading *P*-elements

**DOI:** 10.1101/gr.278706.123

**Published:** 2024-03

**Authors:** Divya Selvaraju, Filip Wierzbicki, Robert Kofler

**Affiliations:** 1 Institut für Populationsgenetik, Vetmeduni Vienna, 1210 Vienna, Austria;; 2 Vienna Graduate School of Population Genetics, Vetmeduni Vienna, 1210 Vienna, Austria

## Abstract

To prevent the spread of transposable elements (TEs), hosts have developed sophisticated defense mechanisms. In mammals and invertebrates, a major defense mechanism operates through PIWI-interacting RNAs (piRNAs). To investigate the establishment of the host defense, we introduced the *P*-element, one of the most widely studied eukaryotic transposons, into naive lines of *Drosophila erecta*. We monitored the invasion in three replicates for more than 50 generations by sequencing the genomic DNA (using short and long reads), the small RNAs, and the transcriptome at regular intervals. A piRNA-based host defense was rapidly established in two replicates (R1, R4) but not in a third (R2), in which *P*-element copy numbers kept increasing for over 50 generations. We found that the ping-pong cycle could not be activated in R2, although the ping-pong cycle is fully functional against other TEs. Furthermore, R2 had both insertions in piRNA clusters and siRNAs, suggesting that neither of them is sufficient to trigger the host defense. Our work shows that control of an invading TE requires activation of the ping-pong cycle and that this activation is a stochastic event that may fail in some populations, leading to a proliferation of TEs that ultimately threaten the integrity of the host genome.

Transposable elements (TEs) are sequences of DNA that selfishly spread in genomes. As this selfish activity enhances the transmission rate, TEs may spread in genomes even if this activity reduces host fitness (Doolittle and Sapienza 1980; Orgel and Crick 1980; Hickey 1982). TEs have been highly successful as they have invaded the genomes of virtually all eukaryotic species investigated so far (Wicker et al. 2007). Although some TE insertions could beneficially impact the host (González et al. 2008; Casacuberta and González 2013), it is assumed that most TE insertions are neutral or deleterious (Nuzhdin 1999; Arkhipova 2018). Theoretical work suggests that the selfish spread of TEs can reduce the fitness of host populations to such an extent that the survival of populations or species is threatened (Kofler 2019, 2020). In agreement with this, experimental populations invaded by a highly active TE went extinct after a few generations (Wang et al. 2023). Because of these deleterious effects, host organisms have developed a broad range of sophisticated defense mechanisms, which frequently involve small RNAs (Sarkies et al. 2015). In mammals and invertebrates, the host defense against TEs is based on piRNAs, small RNAs ranging in size from 23 to 29 nt (Brennecke et al. 2007, 2008; Gunawardane et al. 2007; Senti and Brennecke 2010; Yamanaka et al. 2014; Czech and Hannon 2016; Lewis et al. 2018). These piRNAs bind to PIWI-clade proteins and mediate the repression of TEs at the transcriptional and the post-transcriptional level (Brennecke et al. 2007; Gunawardane et al. 2007; Sienski et al. 2012; Le Thomas et al. 2013). Most piRNAs are derived from discrete genomic source loci, termed piRNA clusters (Brennecke et al. 2007). In *Drosophila melanogaster* about 142 clusters were found, which account for ∼3.5% of the genome (Brennecke et al. 2007; Yamanaka et al. 2014). A central component of the piRNA pathway is the ping-pong cycle, in which piRNAs, bound to the cytoplasmic proteins Aub and AGO3, direct the cleavage of TE transcripts (Brennecke et al. 2007; Gunawardane et al. 2007). Cleavage by AGO3 yields novel piRNAs, which may in turn be loaded into Aub and vice versa. As a result, the ping-pong cycle amplifies the amount of piRNAs targeting a TE. Cleavage by AGO3 (Aub) may also trigger “phasing,” in which cleaved piRNA precursors are further processed into piRNAs by Zuc. These phased piRNAs are mostly bound by Piwi and mediate the transcriptional silencing of the TE in the nucleus. Although the ping-pong cycle amplifies the amount of piRNAs, phasing is thought to increase the diversity of piRNAs targeting a TE (Han et al. 2015; Mohn et al. 2015; Czech et al. 2018). piRNAs bound to PIWI-clade proteins have an important additional function: They are frequently maternally deposited into the egg, and these piRNAs are thought to define the position of piRNA clusters in the following generation (Le Thomas et al. 2014; Hermant et al. 2015). Furthermore, maternally deposited piRNAs likely initiate the ping-pong cycle in the next generation (Le Thomas et al. 2014). Therefore, once a host defense against a TE has been established, maternal piRNAs maintain the host defense against the TE in the next generation by (1) defining the sites of piRNA-producing loci and (2) initiating the ping-pong cycle. However, in the case of a newly invading TE, an important open question remains on how such a piRNA-based host defense gets established in the first place. Under the trap model, an insertion into a piRNA cluster triggers the production of piRNAs against an invading TE, which in turn directs the TE's silencing (Bergman et al. 2006; Malone et al. 2009; Zanni et al. 2013; Goriaux et al. 2014; Yamanaka et al. 2014; Ozata et al. 2019). Recently, it was suggested that maternally inherited siRNAs, produced from Dcr-2-mediated cleavage of dsRNA, could trigger the conversion of a locus into a piRNA-producing site (Luo et al. 2023). Such dsRNA may be readily formed from sense and antisense transcripts of TEs (Luo et al. 2023).

Alternative mechanisms that could trigger the host defense against an invading TE include failed splicing of TE transcripts (Yu et al. 2019), environmentally induced spontaneous formation of piRNA clusters (Casier et al. 2019), and cross talk of piRNAs between TEs, in which piRNAs from different TEs may have some sequence similarity to a newly invading TE (Komarov et al. 2020) To investigate the establishment of the host defense, we monitored *P*-element invasions in experimental populations. The *P*-element is a 2907-bp DNA transposon with four exons that encode a single protein, the transposase (Bingham et al. 1982; O'Hare and Rubin 1983; Majumdar and Rio 2015). The *P*-element is active solely in the germline but not in the soma, which is thought to reduce deleterious fitness effects to hosts (Burt and Trivers 2006). This tissue-specific activity of the *P*-element is regulated by alternative splicing of its third intron (IVS3). Retention of IVS3 in the soma leads to a nonfunctional transposase (Laski et al. 1986). It was recently proposed that the piRNA pathway regulates *P*-element activity by suppressing IVS3 splicing in the germline rather than by regulating the expression of the *P*-element (Teixeira et al. 2017). In contrast to this, reciprocal crosses among flies with and without the *P*-element suggest that *P*-element expression is markedly reduced in offspring with piRNA-based defense against the *P*-element (Khurana et al. 2011; Moon et al. 2018). In addition to piRNAs, *P*-element activity may also be regulated by certain nonautonomous *P*-element insertions containing internal deletions (IDs), such as the *KP*-element or *D50* (Black et al. 1987; Ramussson et al. 1993). Expression of these elements with IDs leads to nonfunctional transposases that may occupy the transposase binding sites and thus prevent functional transposases from mobilizing the *P*-element (Lee et al. 1998).

Here we aim to shed light on the establishment of host defenses against an invading TE, by monitoring *P*-element invasions in experimentally evolving populations of *Drosophila erecta*.

## Results

### *P*-element invasion in *D. erecta*

**Figure 1.**
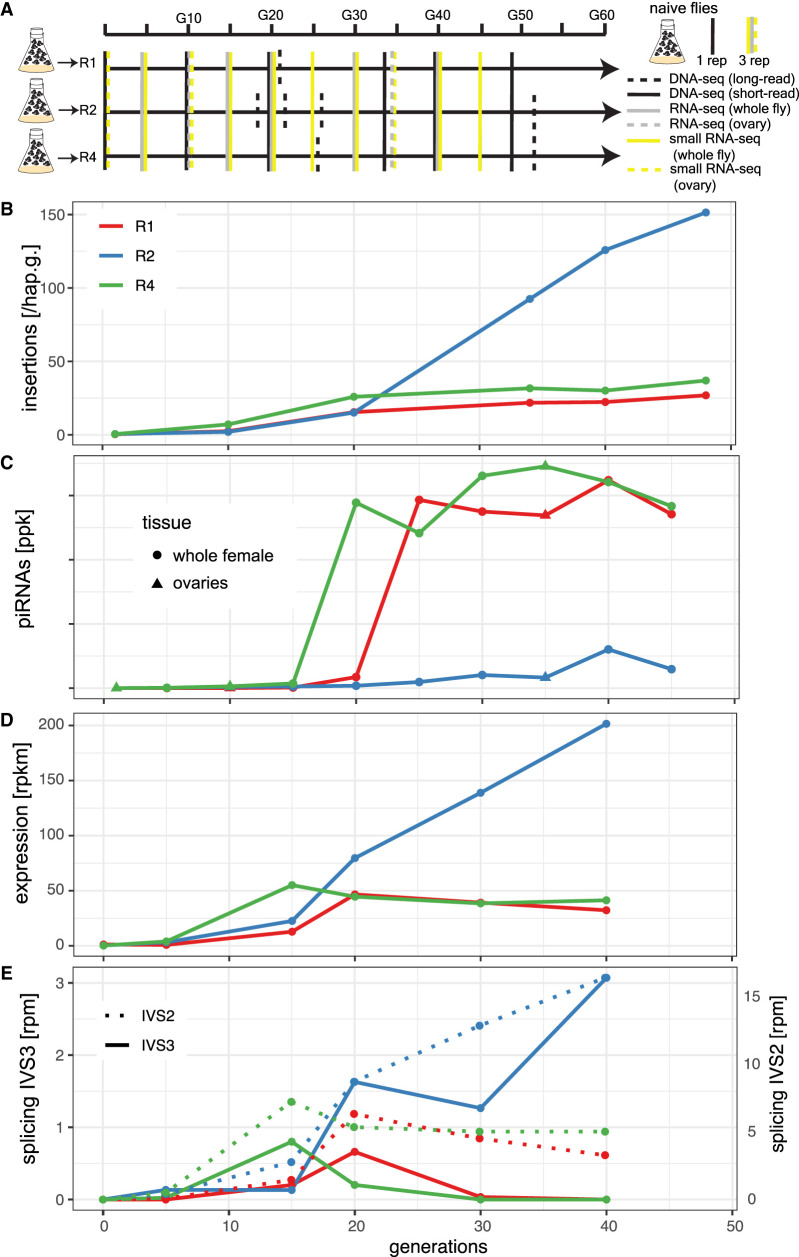
Invasion dynamics of the *P*-element in experimental *D. erecta* populations. Data are shown for three replicate populations (R1, R2, R4). (*A*) Overview of the experiment and the sequenced samples. (*B*) Insertions per haploid genome (hap.g.) during the invasion. (*C*) Abundance of piRNAs complementary to the *P-*element. (ppk) *P*-element piRNAs per 1000 piRNAs. (*D*) Sense expression of the *P*-element in whole female flies. Naive flies are shown at generation zero. (*E*) Splicing of the second (IVS2) and the third (IVS3) intron of the *P*-element. Naive flies are shown at generation zero. (rpm) Spliced reads per million mapped reads.

To investigate the establishment of host control, we monitored *P*-element invasions in experimental populations of *D. erecta*, a species that does not have *P*-element insertions (i.e., naive species) (Brookfield et al. 1984). We used the *D. erecta* strain 01 as this strain is highly inbred and was used for generating the reference genome (*Drosophila* 12 Genomes Consortium 2007; Kim et al. 2021). We first confirmed the absence of the *P*-element in strain 01 using PCR and Illumina sequencing (Supplemental Figs. S1, S2A). Next, we introduced the *P*-element of *D. melanogaster* into the *D. erecta* strain 01 via microinjection of a *P*-element-carrying plasmid into embryos (ppi25.1; kindly provided by Dr. Erin Kelleher). The transformed flies were screened for the presence of the *P*-element using PCR (Supplemental Fig. S2) and maintained in the laboratory for three generations before we mixed them with naive *D. erecta* flies of strain 01. The experimental populations were maintained at a population size of *N* = 250 for 50 generations. We used nonoverlapping generations, three replicates, and a constant temperature of 25°C. As our populations are based on highly inbred lines with a low level of polymorphism, the influence of selection should be minimal (in contrast to previous works in which the *P*-element invaded populations with high levels of standing genetic variation) (Kofler et al. 2018, 2022). We investigated the spread of the *P*-element by monitoring several key parameters: the abundance of *P*-element insertions, the expression and splicing of the *P*-element, and the amount of piRNAs complementary to the *P*-element (Fig. 1A).

To trace the spread of the *P*-element, we sequenced the populations as pools (Pool-seq) (Schlötterer et al. 2014) at about each 10th generation using Illumina paired-end sequencing (for an overview of all used Pool-seq samples, see Supplemental Table S1). We estimated the number of *P*-element insertions with DeviaTE (Weilguny and Kofler 2019), which normalizes the coverage of the *P*-element to the coverage of single-copy genes. Initially, *P*-element copy numbers rapidly increased in all three replicates, but the spread considerably slowed around generation 20 in replicates 1 and 4 (Fig. 1B; Supplemental Figs. S3–S5). By generation 48, each haploid genome carried about 27 and 37 *P*-element insertions in replicates 1 and 4, respectively (Supplemental Table S2). In contrast to this, *P*-element copy numbers continued to increase in replicate 2. By generation 48, each haploid genome accumulated 151 *P*-element copies in replicate 2 (i.e., a staggering 302 *P*-element insertions per fly) (Fig. 1B; Supplemental Table S2). At late generations (34 or greater), *P*-element copy numbers are significantly higher in replicate 2 than in replicates 1 and 4 (Wilcoxon rank-sum test *P* = 0.024) (Supplemental Table S2). The effective transposition rate (*u*′) in replicate 2 is higher than in the other two replicates (Supplemental Table S2). Note that we are solely able to measure the effective transposition rate, that is, the novel insertions gained through transpositions, minus the insertions lost via negative selection against the TEs (*u*′ = *u* − *x*). An analysis independent of DeviaTE, based on the fraction of raw reads mapping to the *P*-element, confirms that the *P*-element proliferates in replicate 2, whereas the invasion is largely controlled by generation 20 in replicates 1 and 4 (Supplemental Fig. S6; Supplemental Table S2). To further substantiate these findings and to investigate the heterogeneity of *P*-element copy numbers within replicates, we sequenced 12 individual flies for each replicate at generation 42 (Supplemental Fig. S7). Different flies within a given replicate had similar copy numbers, but flies from replicate 2 had significantly higher copy numbers than did flies from replicates 1 and 4 (Wilcoxon rank-sum test *W* = 338, *P* = 2.5 × 10^−10^) (Supplemental Fig. S7), further supporting the proliferation of the *P*-element in replicate 2 relative to the other replicates. We found that *D. erecta* flies carrying the *P*-element may induce atrophied ovaries (gonadal dysgenesis [GD]) in the offspring of crosses with naive flies not having the *P*-element (Supplemental Figs. S16, S17; Supplemental Table S6). Of note, replicate 2 could not be maintained beyond generation 62 because not enough female flies eclosed at the experimental conditions (25°C). We were able to rescue the population by back-crossing females of replicate 2 to naive males. It is likely that the accumulating load of deleterious *P*-element insertions was driving replicate 2 nearly to extinction (for fecundity at generation 88, see Supplemental Fig. S8; see also Lama et al. 2022).

In *Drosophila*, TE activity is primarily controlled by piRNAs, small RNAs ranging in size between 23 and 29 nt (Brennecke et al. 2007; Gunawardane et al. 2007). To test whether piRNAs against the *P*-element emerged in our experimental populations, we sequenced small RNAs at every fifth generation during the experiment. Initially, we aimed to sequence ovarian RNA. Because of the workload associated with repeated dissections of large numbers of ovaries, we sequenced whole bodies of female flies at later generations (for an overview of all sequenced small RNA libraries, see Supplemental Table S3). We normalized the abundance of small RNAs to a million piRNAs as this yields comparable estimates of piRNA abundance among ovaries and whole bodies of females (Supplemental Fig. S9).

Only very few piRNAs complementary to the *P*-element were found in the naive *D. erecta* strain 01 (one read in the ovaries and three reads in the whole bodies) (Fig. 4). In the experimental populations, piRNA copy numbers rapidly increased in replicates 1 and 4 around generation 20 but remained at a significantly lower level in replicate 2 (Wilcoxon rank-sum test at generations of 25 and greater, *P* = 0.00067) (Fig. 1C). In replicates 1 and 4, most piRNAs have a length between 25 and 27 nt and are antisense to the *P*-element, as expected for piRNAs (Supplemental Fig. S10; Vagin et al. 2006; Brennecke et al. 2007). Furthermore, these piRNAs show a pronounced U-bias at the first nucleotide, as described for piRNAs bound to Aub or Piwi (Supplemental Fig. S11; Saito et al. 2006; Brennecke et al. 2007) By generation 45, the piRNAs are distributed along the *P*-element in replicates 1 and 4, but very few piRNAs are found along the *P*-element in replicate 2 (Supplemental Fig. S12). Our data thus suggest that the *P*-element is largely controlled by piRNAs in replicates 1 and 4 around generation 20–25, whereas the abundance of piRNAs may be insufficient to stop the *P*-element invasion in replicate 2 (Fig. 1).

We next asked how piRNAs act to control the *P*-element invasion in replicates 1 and 4. It is an open question on whether piRNAs regulate the expression or the splicing of the third intron (IVS3) of the *P*-element (Khurana et al. 2011; Teixeira et al. 2017; Moon et al. 2018). We thus performed stranded RNA-seq (poly[A] selected) at about each 10th generation (for an overview of the RNA-seq data, see Supplemental Table S4). Although very few RNA-seq reads map to the *P*-element in naive flies (zero to one reads), large numbers of reads align to the *P*-element in our experimental populations (Supplemental Table S4). The position of the *P*-element introns is identical in *D. erecta* and *D. melanogaster* (Supplemental Figs. S13, S14). In replicates 1 and 4, the expression (sense) of the *P*-element in whole flies increases until generations 15–20 and remains at a high level thereafter (Fig. 1D; Supplemental Fig. S13). In replicate 2, the expression of the *P*-element increases until generation 40. We additionally sequenced *P*-element expression in ovaries at generations 10 and 35 (Supplemental Fig. S14). In ovaries, the *P*-element expression increased from generations 10 to 35 (Supplemental Fig. S14). At generation 35, the *P*-element expression was even higher in ovaries than in whole flies (rpkm) (Supplemental Figs. S13, S14). Our data thus suggest that the emergence of piRNAs does not cause a marked decrease in *P*-element expression. In contrast, the level of splicing of IVS3 showed a pronounced response to the emergence of piRNAs (Fig. 1E). In replicates 1 and 4, the level of splicing of IVS3 increased until generations 15–20, but splicing of IVS3 stopped at later generations (Fig. 1E; Supplemental Figs. S13, S14). Only in replicate 2, in which few piRNAs complementary to the *P*-element emerged, did the level of splicing of IVS3 continue to increase. In the ovaries, we also found that splicing of IVS3 stopped by generation 35 in replicates 1 and 4 but not in replicate 2 (Supplemental Fig. S14). In contrast to IVS3, the number of spliced reads for the other two introns of the *P*-element (IVS1, IVS2) remained at a high level in all replicates (Fig. 1E; Supplemental Fig. S15). A linear model suggests that piRNAs have a significant negative effect on the splicing level of IVS3 and IVS1 but no effect on the splicing level of IVS2 or on the expression level of the *P*-element (*P* < 0.01) (Supplemental Table S5). To test whether the effect of piRNAs on splicing is independent of the expression level of the *P*-element, an additional linear model, in which *P*-element expression and piRNA abundance were used as independent variables, confirms that piRNAs have a significant negative effect on the splicing of IVS3 (*P* = 0.0057; no significant effect was found for IVS1 and IVS2) (Supplemental Table S5). Overall, our data support the hypothesis of Teixeira et al. (2017) that *P*-element piRNAs primarily act to repress the splicing of the *P*-element's third intron, thus preventing the expression of functional *P*-element transcripts.

### Ping-pong is not active in replicate 2

**Figure 2.**
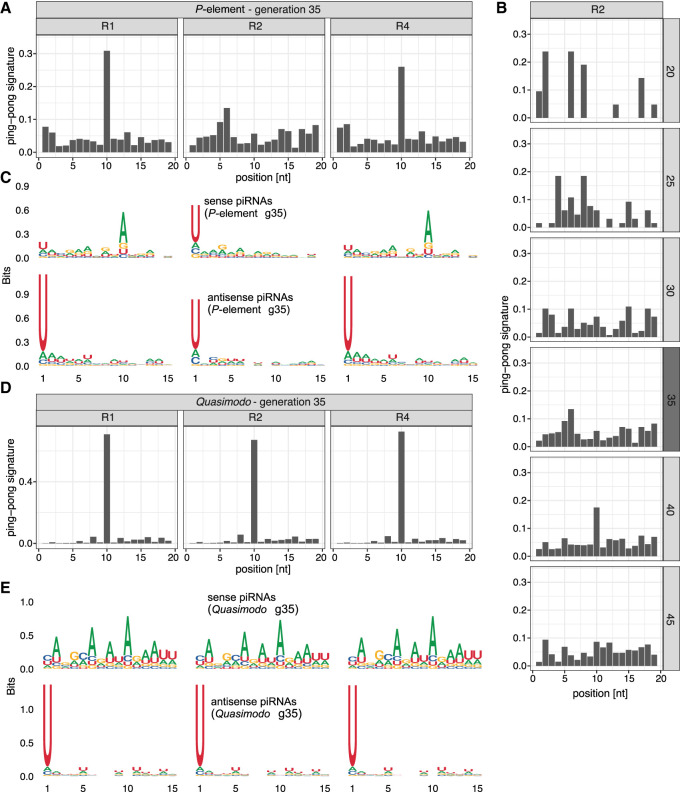
*P*-element ping-pong is inactive in replicate 2. (*A*) The ping-pong signature (peak at position 10) of the *P*-element in ovaries at generation 35. Note that the ping-pong signature is missing in replicate 2. (*B*) The ping-pong signature of the *P*-element in replicate 2. Data are shown for all generations with sufficient piRNAs. Small RNA extracted from ovaries and whole flies is shown in dark and light gray, respectively. (*C*) Motifs of sense and antisense piRNAs (23–29 nt) complementary to the *P*-element in ovaries at generation 35. Sense piRNAs in replicate 2 do not show an A-bias at position 10. (*D*) *Quasimodo* displays a pronounced ping-pong signature in all replicates (ovaries, generation 35). (*E*) Motifs of sense and antisense piRNAs of *Quasimodo* (ovaries, generation 35).

Although replicate 2 has some piRNAs complementary to the *P*-element, the abundance of these piRNAs was lower than in the other replicates. We thus asked whether the ping-pong cycle is active for the *P*-element in replicate 2. Because of the interaction between the PIWI clade proteins Aub and AGO3, an active ping-pong cycle generates a characteristic peak at position 10 when plotting the distance between the 5′ ends of sense and antisense piRNAs, that is, the ping-pong signature (Brennecke et al. 2007; Gunawardane et al. 2007). In ovaries sampled at generation 35, we observed a clear ping-pong signature in replicates 1 and 4 but not in replicate 2, which suggests that the ping-pong cycle is inactive in replicate 2 (Fig. 2A). This absence of the peak at position 10 in replicate 2 was observed at all generations with sufficient amounts of piRNAs, irrespective of whether the small RNAs were extracted from whole flies or ovaries (Wilcoxon rank-sum test comparing the peak at position 10 between R2 and R1 + R4 for generations 20 to 45:*W* = 1, *P* = 0.0012) (Fig. 2B; Supplemental Fig. S26A). Because ping-pong signatures may be sensitive to the amount of piRNAs, we subsampled the number of piRNAs to identical numbers across replicates and again found ping-pong signatures in replicates 1 and 4 but not in replicate 2 (Supplemental Fig. S26B). For most TE families, antisense piRNAs are largely bound to Piwi and Aub, whereas sense piRNAs are frequently associated with AGO3 (Brennecke et al. 2007; Senti et al. 2015). Piwi- and Aub-bound piRNAs frequently show a strong U-bias at position 1, whereas AGO3-bound piRNAs show an A-bias at position 10 (Brennecke et al. 2007; Gunawardane et al. 2007; Czech and Hannon 2016). We find a pronounced U-bias at position 1 for antisense piRNAs of the *P*-element in all three replicates, in agreement with expectations for piRNAs (Fig. 2C; Supplemental Fig. S28). We also find the A-bias at position 10 for sense piRNAs in replicates 1 and 4 but not in replicate 2, where we find a U-bias at position 1 instead (Fig. 2C; Supplemental Fig. S27). This suggests that the ping-pong cycle is inactive in replicate 2, because AGO3-bound piRNAs are absent.

Our findings raise the question of whether the ping-pong cycle is defective in replicate 2. We thus investigated the ping-pong signatures and the small RNA motifs of *Quasimodo* (LTR) and *BS* (non-LTR). We observed a high contiguous coverage of these two TEs in *D. erecta*, suggesting that *D. erecta* has full-length copies of *Quasimodo* and *BS*. *BS* and *Quasimodo* show notable ping-pong signatures in all three replicates (Fig. 2D; Supplemental Fig. S29). Furthermore, the U-bias at position 1 of antisense piRNAs and the A-bias at position 10 of sense piRNAs are also present in all three replicates (Fig. 2E; Supplemental Fig. S29). Our data thus suggest that the ping-pong cycle is fully functional in all three replicates, including replicate 2.

In summary, we show that the ping-pong cycle against the *P-*element is active in replicates 1 and 4 but not in replicate 2, although the ping-pong cycle is fully functional in all three replicates.

### Insertions in piRNA clusters

We next investigated the reasons as to why the initiation of the ping-pong cycle failed in replicate 2 but not in replicates 1 and 4. It is usually thought that the host defense, including the ping-pong cycle, is triggered when a copy of the invading TE jumps into a piRNA cluster (trap model). One important aspect that is frequently neglected in functional discussions of the trap model is that a TE insertion in a piRNA cluster will initially be solely present in a single individual of the population (i.e., one out of 250 flies in our experiment), and thus, the TE is initially silenced only in a single individual. To inactivate the TE throughout the population, the piRNA-producing loci need to spread to all individuals of a population. For this, three hypotheses are feasible.

**Figure 3.**
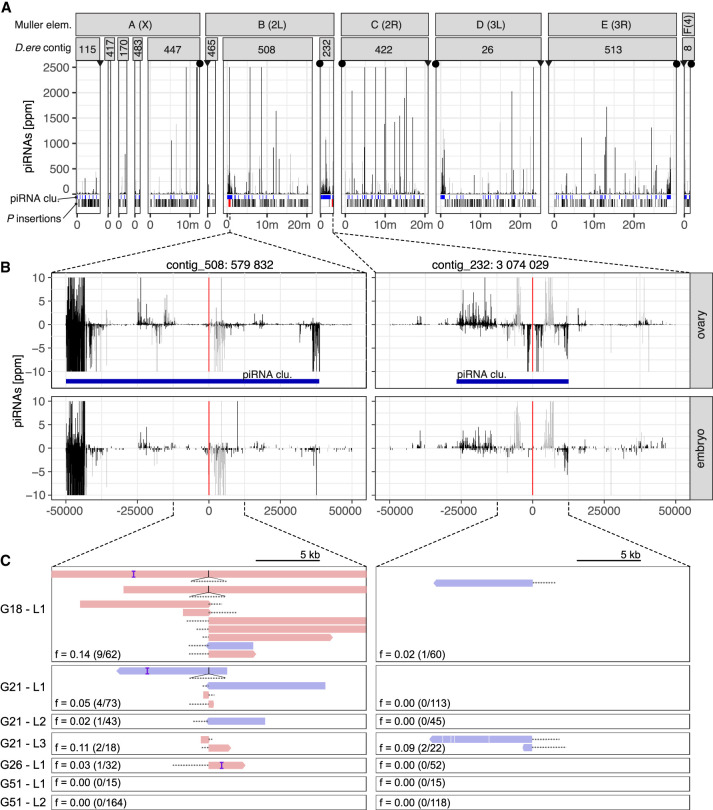
At least two *P*-element insertions in germline clusters are present in replicate 2 at early stages of the invasion (generations 18–26). (*A*) Overview of ambiguously (light gray) and unambiguously (black) mapping piRNA in 1-kb windows along the 12 largest contigs of the *D. erecta* assembly. The corresponding Muller element and the likely direction of the telomere (triangle) and the centromere (circle) are shown. At the *bottom*, we show the positions of the annotated piRNA clusters (blue) of *P*-element insertions outside (black) and inside of piRNA clusters (red bold; solely insertions in replicate 2 at generations 26 or less supported by at least two long reads are shown). (*B*) Abundance of piRNAs in a 100-kb window around the two insertions in piRNA clusters for both ovaries and embryos. Sense piRNAs are on the positive *y*-axis and antisense piRNAs on the negative *y*-axis. Ambiguously (light gray) and unambiguously (black) mapped reads are shown. (*C*) Support of the *P*-element insertions by long reads at different generations (*Gxx*) and in different sequencing libraries (*Lx*). Sense reads are shown in red, and antisense reads are in blue. Regions of the reads aligning to the *P*-element are shown as dashed lines (true to scale). For each library, we indicate the population frequency of the *P*-element insertion (*f*), the number of reads supporting the insertion, and coverage at the insertion site (in parentheses: reads/coverage). Note that both insertions are likely full-length *P*-element insertions (almost the complete *P*-element is covered by some reads) and that both insertions are likely lost (or at very low frequency) by generation 51.

First, a cluster insertion may be positively selected and sweep through the population until all individuals carry the same cluster insertion (sweep model) (Blumenstiel 2011). Under this sweep model, we expect to find at least one fixed cluster insertion in replicates with an active host defense (i.e., replicate 1 and replicate 4). To find *P*-element insertions in piRNA clusters, we relied on a recent long-read assembly of *D. erecta* (Kim et al. 2021). The annotation of the piRNA clusters was based on small RNA data from ovaries of naive *D. erecta* flies and a previously described algorithm (Kofler et al. 2018, 2022). We also sequenced small RNAs from embryos of naive *D. erecta* flies to distinguish germline (dual-strand clusters) from somatic clusters (uni-strand clusters). To identify *P*-element insertions in the repetitive piRNA clusters with high confidence, we sequenced the experimental populations at multiple generations using long-read sequencing (for an overview of the long-read data, see Supplemental Table S7). We did not find a single fixed cluster insertion around generation 20 in any sample from all three replicates (Supplemental Table S8). A cluster insertion in contig_508 at generation 18 of replicate 2 had the highest population frequency among all replicates (G18-L1:*f* = 0.14) (Fig. 3C; Supplemental Table S8). An analysis of the population frequencies of *P*-element insertions based on Illumina short-read data suggests that not a single *P*-element insertion is fixed in the experimental populations (Supplemental Figs. S19–S21). Our data are thus not compatible with the sweep model.

Second, it was proposed that because of a high TE activity, many different cluster insertions emerge independently, such that a TE invasion will be stopped by several segregating cluster insertions (shotgun model) (Kelleher et al. 2018; Kofler et al. 2018; Kofler 2019). Under this scenario, each individual in a population will carry a distinct set of cluster insertions. Computer simulations under this scenario show that a TE invasion is stopped when each diploid individual carries, on average, about four cluster insertions, although it was assumed during the simulations that a single insertion per diploid is sufficient to silence the TE (Kofler 2019). Recombination and random assortment among segregating cluster insertions will lead to a distribution of cluster insertions in populations, in which some individuals will end up with several cluster insertions and others with just a few or even none. The TE will be active in the individuals without a single cluster insertion. Solely when diploids carry an average of about four cluster insertions, the vast majority of the offspring will end up with at least one cluster insertion. Under this model, we expect to find around four cluster insertions in replicates with an active host defense (i.e., replicate 1 and replicate 4). Based on our long-read data, we find that all replicates solely carry between 3% and 29% of the required number of cluster insertions (*R*1 = 10%, *R*2 = 3% − 29%, *R*4 = 6%) (Supplemental Table S9). Therefore, our data are not in agreement with the shotgun model. This is also consistent with our previous work, in which we found an insufficient number of cluster insertions in experimental *Drosophila simulans* populations being invaded by the *P*-element (Kofler et al. 2018, 2022).

Finally, it was noted that dispersed TE insertions may generate piRNAs (Mohn et al. 2014; Shpiz et al. 2014). Because the deletion of large piRNA clusters did not lead to an activation of TEs, it was proposed that these dispersed TEs have an important role in the silencing of TEs (Gebert et al. 2021). The conversion of a regular TE insertion into a piRNA-producing locus may be triggered by maternally deposited piRNAs (De Vanssay et al. 2012; Le Thomas et al. 2014; Hermant et al. 2015). In the case of an invasion of a novel TE, the dependency of TE-conversions on maternally deposited piRNAs raises an important question on the origin of the very first piRNAs that could trigger them (Scarpa and Kofler 2023). One option is that one insertion into a piRNA cluster triggers the origin of the very first piRNAs complementary to an invading TE. Once such initial piRNAs have emerged, increasing numbers of regular TE insertions, in different individuals, may be converted into piRNA-producing loci as the invasion progresses (TE-conversion model). Under this model, we expect to find at least one cluster insertion, possibly at a low population frequency in replicates 1 and 4 but not in replicate 2. Although we found some cluster insertions at early generations (around generation 20) in replicates 1 and 4 (Supplemental Table S8; Supplemental Fig. S18), we also found two cluster insertions in replicate 2 (Fig. 3A). These cluster insertions in replicate 2 are likely reliable, as they are supported by several long reads from different strands in multiple sequencing libraries of distinct generations (Fig. 3C). As piRNAs are found in both the ovaries and embryos of naive flies and piRNAs align to both strands, the two insertion sites are likely present in germline clusters (dual-strand clusters) (Fig. 3B; Malone et al. 2009; Czech et al. 2018). An analysis of the genomic short-read data also supports the idea that cluster insertions are present in all replicates, including replicate 2, at early generations (albeit different ones) (Supplemental Fig. S20). Consequently, our data suggest that the cluster insertions in replicate 2 were not sufficient to trigger the ping-pong cycle.

In summary, we are able to rule out the two hypotheses that a fixed cluster insertion (sweep model) or several segregating cluster insertions (shotgun model) control TE invasion. Because we found a few cluster insertions in all replicates, we cannot rule out that these cluster insertions drive the conversion of regular *P*-element insertions into piRNA-producing loci. Although it is feasible that cluster insertions are a precondition for activating the host defense, our data also suggest that *P*-element insertions in piRNA clusters are insufficient to activate the ping-pong cycle.

### siRNAs emerge before piRNAs

In addition to piRNAs, siRNAs (20–22 nt) may also contribute to the silencing of TEs (Chung et al. 2008; Czech et al. 2008; Barckmann et al. 2018). In contrast to piRNAs, which are solely found in the germline of most *Drosophila* species, siRNAs are found both in the germline and the somatic cells (Lewis et al. 2018). The siRNA pathway is distinct from the piRNA pathway, relying on entirely different sets of enzymes (e.g., Dcr-2, AGO2) (Vagin et al. 2006; Czech et al. 2008). A recent work suggested a link between the piRNA and siRNA pathway (Luo et al. 2023). siRNA-guided slicing of a complementary transcript might create new piRNAs, which in turn act at the chromatin level and thereby convert a locus (e.g., a TE insertion) into a piRNA-producing locus (Luo et al. 2023). Dispersed TE insertions could generate sense and antisense transcripts, which may form dsRNA (and thus siRNAs). In agreement with this, we found that in our RNA-seq libraries ∼7.41% of all *P*-element transcripts are antisense. As a control, solely 0.25% of the reads aligning to all *D. erecta* transcripts are antisense, which is significantly lower than for the *P*-element (Wilcoxon rank-sum test *W* = 0, *P* = 7.4 × 10^−10^). Antisense transcripts of the *P*-element were found in all replicates already at generation 5 (Supplemental Fig. S22). Furthermore, antisense transcripts were found in RNA extracted from ovaries and whole flies (Supplemental Fig. S22). Therefore, the substrate necessary for generating siRNAs is likely already present in early generations of our experimental populations.

**Figure 4.**
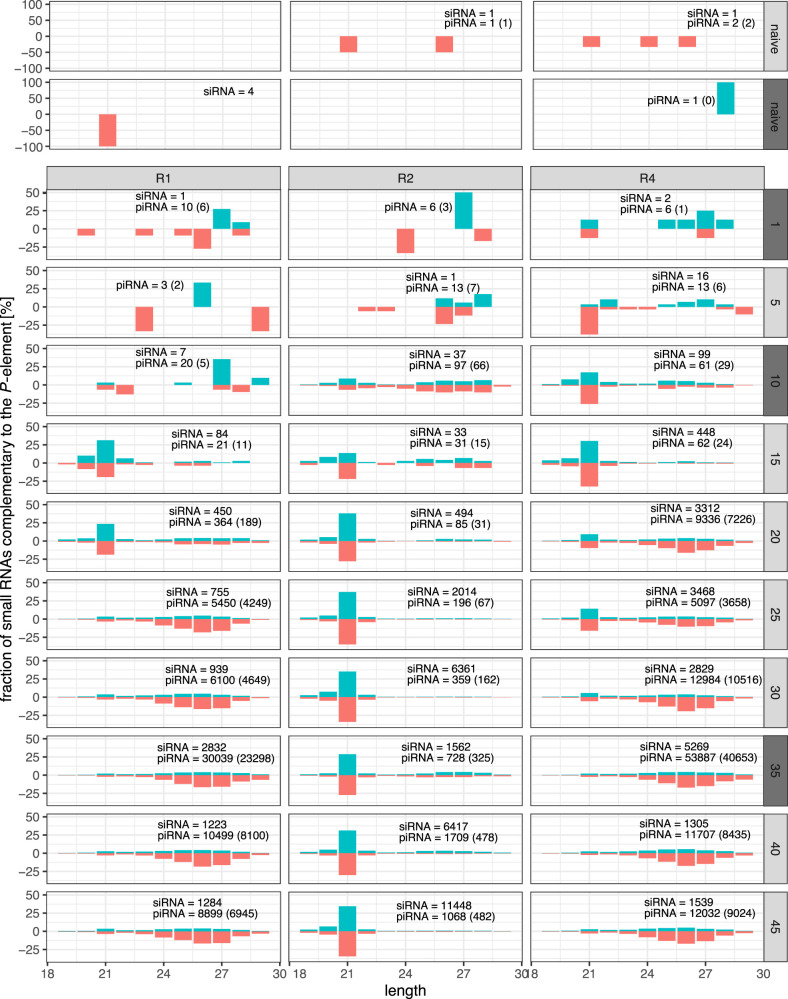
Length distribution of small RNAs mapping to the *P*-element during the invasions. Data are shown for naive flies and the three replicates (*top* panel) at all five generations (*right* panel). We extracted small RNAs, either from whole bodies of female flies (light gray panels) or from ovaries (dark gray panels). For each sample, we show the percentage of the small RNAs mapping to a given size category (hence the total number of reads adds up to 100% in each sample). Sense RNAs are on the positive *y*-axis, and antisense RNAs are on the negative *y*-axis. The total number of siRNAs (20–22 nt) and piRNAs (23–29 nt) mapping to the *P*-element are shown (antisense piRNAs are in parentheses).

If siRNAs activate piRNA-producing loci against the *P*-element (Luo et al. 2023), we expect siRNAs to emerge before piRNAs in the experimental populations. We monitored the length distribution of small RNAs during the invasions at every fifth generation. We assumed that small RNAs with a length between 20 and 22 nt and between 23 and 29 nt correspond to siRNAs and piRNAs, respectively. In naive flies and at early generations of the experimental populations, few small RNAs aligning to the *P*-element were found, and the length distribution of these RNAs was nonspecific (Fig. 4). However, around generations 10–15, the siRNAs peaks emerged in all replicates. Apart from the distinct length (∼21 nt), these small RNAs have additional features typical for siRNAs, such as the balanced strand bias (Fig. 4) and a less pronounced 5′-U-bias than the piRNAs (Supplemental Figs. S11, S23; Czech et al. 2008; Ghildiyal et al. 2008; Kemp and Imler 2009). These siRNAs were distributed over the entire sequence of the *P*-element (Supplemental Fig. S24). The piRNA peaks, with lengths between 23 and 29 nt, emerged only at later generations in replicates 1 and 4 but not in replicate 2 (Fig. 4). Although the abundance is much lower than in the other replicates, the presence of antisense piRNAs in R2 suggests that at least some antisense transcripts of *P*-element insertions are processed into piRNAs in this replicate. In replicate 2, the abundance of siRNAs reached similar levels as the abundance of piRNAs in replicates 1 and 4 (Supplemental Fig. S25). However, in replicate 2, the abundance of siRNAs was much lower in the ovarian sample than in whole flies (generation 35) (Supplemental Fig. S25), suggesting that many siRNAs are of somatic origin. Nevertheless, abundant siRNAs were also present in the ovarian samples of all replicates (generation 35) (Fig. 4).

In summary, we observed that siRNAs emerged before piRNAs, which is expected under the hypothesis of Luo et al. (2023) that siRNAs trigger the silencing of a TE. However, our data also suggest that siRNAs are insufficient to trigger the piRNA-based host defense, as abundant *P*-element siRNAs were also found in replicate 2.

### Differences among replicates

Our results raise the important question as to why the ping-pong cycle is inactive against the *P*-element solely in replicate 2. Sense and antisense transcripts of TEs are the substrates of the ping-pong cycle. These transcripts are sliced by Aub and AGO3, thereby generating novel piRNAs (Brennecke et al. 2007; Czech et al. 2018). However, we showed that all replicates have a similar ratio between sense and antisense transcripts of the *P*-element (Supplemental Fig. S22). Furthermore, given that replicate 2 has the highest expression level of the *P*-element (Fig. 1D), a lack of substrate cannot explain the absence of ping-pong in replicate 2.

It is feasible that there is a trade-off between the defense against TEs and viruses (Roy et al. 2020). A virus infection may, for example, preoccupy important enzymes from the siRNA or piRNA pathway such that they are no longer available for establishing a defense against an invading TE. We thus investigated the amount of small RNAs mapping to different *Drosophila* viruses but found similarly low numbers of reads aligning to viruses in all replicates (Supplemental Table S10).

A recent landmark study by Moon et al. (2018) identified the gene *lok* (*Chk2*) as a crucial factor for triggering the ping-pong cycle against the *P*-element. We thus speculated that the expression of this gene might be aberrant in replicate 2. However, FBtr0141271, the *D. erecta* ortholog of *lok*, has a very similar expression level among the three replicates at all time points (Supplemental Fig. S30).

**Figure 5.**
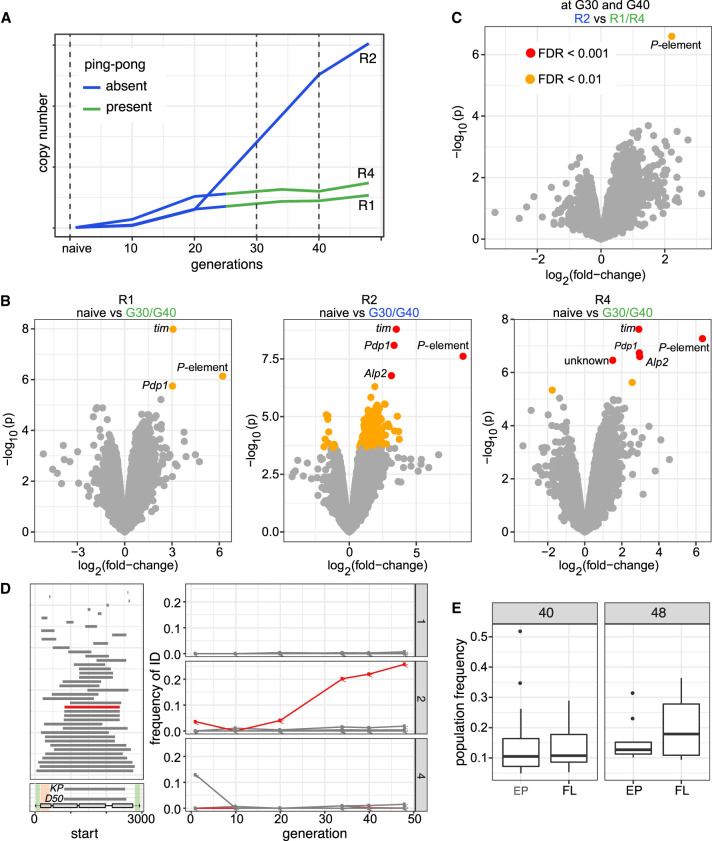
Differences among replicates with (R1, R4) and without (R2) *P*-element ping-pong. (*A*) Schematic overview of the RNA-seq samples used for identifying genes differentially expressed among samples with (green) and without (blue) *P*-element ping-pong. (*B*) Volcano plots highlighting expression differences for TEs and *D. erecta* transcripts between naive flies and invaded flies (generations 30 and 40). Data are shown for all three replicates (R1, R2, R4). (*C*) Volcano plot highlighting expression differences between replicates with and without *P*-element ping-pong at generations 30 and 40. (*D*) Overview of *P*-element insertions with internal deletions in the experimental populations (*left*). The *lower left* panel shows the composition of the *P*-element and the deleted regions of *D50* and the *KP*-element. The DNA-binding domain (orange) and regions necessary for mobilizing the *P*-element (green) are indicated (Majumdar and Rio 2015). The *right* panel shows the frequency of the IDs (relative to all *P*-element insertions) in the three replicates. Note that the *EP*-element (red), that is, a *P*-element variant with deletion of a similar region than for the *KP*-element, is increasing in frequency in replicate 2 but not in the other replicates. (*E*) Population frequency of *EP*-element insertions and the full-length (FL) insertions of the *P*-element in replicate 2 at generations 40 and 48.

It is possible that we have not yet identified all genes required for triggering the ping-pong cycle. Thus, we asked whether any of the *D. erecta* transcripts are differentially expressed among replicates with and without *P*-element ping-pong (Fig. 5A; Supplemental Fig. S26). Orthology to *D. melanogaster* genes was established with BLAST. When comparing the naive flies and the evolved lines at generations 30 and 40 at which R1 and R4 but not R2 have *P*-element ping-pong (Supplemental Fig. S26), we mostly found that the *P*-element and some genes involved with circadian rhythm (*tim*, *Pdp1*) were differentially expressed (Fig. 5B). The differential expression of circadian rhythm genes likely reflects slight differences in sampling times between evolved and naive populations. None of the differentially expressed genes were specific to replicate 2. We next asked if at generations 30 and 40 (at which R1 and R4 but not R2 have *P*-element ping-pong) any genes are differentially expressed between replicates with (R1, R4) and without (R2) *P*-element ping-pong. Except for the *P*-element, which is most highly expressed in replicate 2, we did not detect any significant expression differences among these replicates (Figs. 1D, 5C). Our data thus suggest that the aberrant expression of genes necessary for triggering the ping-pong cycle in replicate 2 is not responsible for the absence of *P*-element ping-pong in this replicate.

We next asked if differences in the sequence of the *P*-element might be responsible for the observed differences among the replicates. In terms of base substitutions, the sequence of the *P*-element is highly similar among all replicates. Only a few rare SNPs were found in all replicates (Supplemental Table S11). We were particularly interested in whether the abundance of IDs of the *P*-element varies among replicates, as some IDs may repress *P*-element activity (Black et al. 1987; Ramussson et al. 1993). IDs arise from interruption of the repair of gaps resulting from the excision of a *P*-element (Engels et al. 1990). We used our previously published tool DeviaTE (Weilguny and Kofler 2019) to identify the location and abundance of IDs within the *P*-element. Based on split-reads (reads mapping to the breakpoints of an ID), DeviaTE quantifies the abundance of IDs relative to the total abundance of *P*-element insertions, but it is not possible to identify the genomic location of the IDs. We found 43 *P*-element IDs in the experimental populations (Fig. 5D). The vast majority of the IDs (42/43) occurred in a single replicate, confirming our previous finding that IDs of the *P*-element are usually replicate-specific (Weilguny and Kofler 2019). Most of the IDs remained at a low frequency. However, one ID, in which nucleotides 827–2375 are deleted (henceforth “*EP*-element”; for erecta *P*-element) rose to a frequency of 25.8% in replicate 2 (Fig. 5D; Supplemental Figs. S3–S5).

In *D. melanogaster*, some insertions with IDs, like *D50* or the *KP*-element, repress *P*-element activity (Black et al. 1987; Ramussson et al. 1993). We were interested in whether the *EP*-element might be one such repressor of *P*-element activity. The proteins encoded by the *EP-* and *KP*-elements are quite similar. Both deletions lead to a premature stop codon, where the resulting protein has a length of 207aa and 208aa for the *KP*-element and the *EP*-element, respectively. Furthermore, for the *EP*-element, the first 206 codons are identical to the full-length *P*-element (which has 751 codons) (Ghanim et al. 2020), whereas for the *KP*-element, the first 199 codons are identical to the full-length element. Hence, the *EP*-element retains the DNA-binding domain (the first 88 codons) (Lee et al. 1998) but probably does not produce a functional transposase (the vast majority of the codons are missing). Similarly to the *KP*-element, the *EP*-element is therefore likely a repressor of *P*-element activity.

This raises the question of why the *EP*-element rose to a high frequency in replicate 2. In principle, two hypotheses are viable. The *EP*-element may be positively selected (as it reduces deleterious *P*-element activity), or it could be preferentially mobilized. These two hypotheses can be distinguished by investigating the population frequency of the different *P*-element insertions as positive selection increases the population frequency of beneficial insertions. If *EP*-elements are positively selected, their population frequency should be higher than the frequencies of full length (FL) insertions. In contrast, preferential mobilization leads to many novel insertions, and novel insertions initially have a low population frequency (1/ 2N). If *EP*-elements are preferentially mobilized, their population frequency should, on average, be lower than the frequency of FL insertions. To address this question, we linked the information obtained from short- and long-read sequencing. Long-read sequencing provides the identity of *P*-element insertions (e.g., to distinguish between *EP*-elements and FL insertions), whereas short-read sequencing provides estimates of the population frequency of *P*-element insertions. Based on the long-read data from generation 51 and the short-read data from generations 48 and 40, we found that *EP*-elements have a lower (albeit not significantly) population frequency than FL insertions (Wilcoxon rank-sum test *P*_48_ = 0.31, *P*_4o_ = 0.64) (Fig. 5E). The high abundance of the *EP*-element in replicate 2 can thus most likely be explained by preferential mobilization of the *EP*-element. This is also in agreement with previous works suggesting that *P*-element insertions with IDs may be more readily mobilized than FL insertions (Itoh et al. 2007; Kofler et al. 2018; Srivastav et al. 2019).

In summary, out of the investigated factors (viral load, fraction of antisense transcripts, *lok* expression, expression pattern of known genes, SNPs, and IDs of the *P*-element), solely the abundance of an ID, the *EP*-element, was substantially higher in replicate 2 than in the other replicates.

### Influence of the genome versus maternally transmitted factors

**Figure 6.**
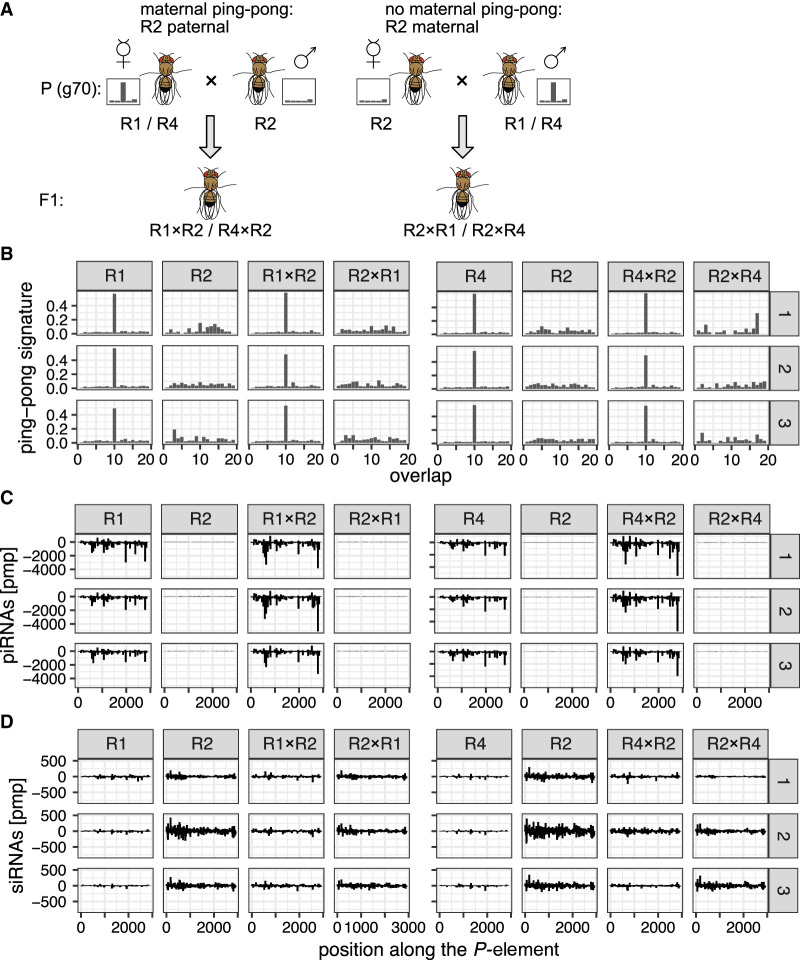
Maternally transmitted factors (possibly small RNAs), and not the genomic composition, are responsible for the absence of *P*-element ping-pong in replicate 2. (*A*) Crossing scheme for testing the influence of maternal piRNAs. We performed reciprocal crosses among replicates with (R1, R4) and without (R2) ping-pong signature for the *P*-element. Note that in the F_1_ offspring of the reciprocal crosses (e.g., R1 × R2 vs. R2 × R1), the genomic background is largely identical, whereas the composition of the maternally deposited piRNAs differs. The flies for this experiment were sampled around generation 70 from the experimental populations. (*B*) Ping-pong signatures for the *P*-element in all three replicates (R1, R2, R4) and in the F_1_ offspring of the reciprocal crosses among the replicates (e.g., R1 × R2: R1-female × R2-male). Small RNA was extracted from whole female flies, and three subreplicates (*right* panel) were used for each sample. (*C*) Distribution of piRNAs (23–29 nt) in the three replicates and the F_1_ offspring. (*D*) Distribution of siRNAS (20–22 nt) in the three replicates and the F_1_ offspring.

The presence of the *EP*-element in replicate 2 is the only notable difference among replicates with (replicates 1, 4) and without (replicate 2) *P*-element ping-pong. We were thus wondering if the *EP*-element could interfere with *P*-element ping-pong in replicate 2. To test this hypothesis, we performed reciprocal crosses among flies sampled from the replicates. Reciprocal crosses among replicates enable us to distinguish between the influence of the genome (which is identical among offspring of the reciprocal crosses) and the influence of maternally transmitted factors (which differs among the offspring of reciprocal crosses), such as small RNAs (Fig. 6A). Flies for the crosses were sampled between generations 67 and 70. For each cross, we set up three subreplicates. We sequenced the small RNAs of the female parents and of the female F_1_ offspring (Fig. 6A). Note that we sequenced in total six independent samples for the parents of replicate 2 (because they participate in two crosses) and three samples for the parents of replicates 1 and 4.

Our small RNA data show that even by generation 67, all six samples of replicate 2 do not show a ping-pong signature (Wilcoxon rank-sum test comparing the peak at position 10 between R2 and R1 + R4:*W* = 0, *P* = 0.024) (Fig. 6B), although the ping-pong cycle is fully functional in all subreplicates (Supplemental Fig. S31). The piRNAs and siRNAs were evenly distributed along the *P*-element (Fig. 6C,D). As seen before, replicate 2 had fewer piRNAs but more siRNAs compared with replicates 1 and 4 (Fig. 6C,D).

Given that we found 74 *P*-element insertions in piRNA clusters in replicate 2 by generation 51 (based on long-read sequencing), our data confirm that cluster insertions are not sufficient to trigger the host response against an invading TE. However, the offspring of crosses among replicates only shows ping-pong signatures if the female is sampled from replicate 1 or 4 but not if females were sampled from replicate 2 (Fig. 6B). As the genome is largely identical among offspring of the reciprocal crosses (e.g., R1 × R2 vs. R2 × R1), these results suggest that maternally transmitted factors are responsible for the absence of ping-pong in replicate 2. Although any maternally transmitted component, such as imprinting or the abundance of some protein, could be responsible, we suspect that maternally transmitted piRNAs might be responsible as these piRNAs are important to trigger the ping-pong cycle in the next generation (Le Thomas et al. 2014).

In summary, reciprocal crosses among replicates show that differences in maternal transmitted factors among replicates (possibly small RNAs), and not the genomic composition, are responsible for the absence of ping-pong in replicate 2. By excluding an influence of the genome, we can rule out an influence of the *EP*-element, of differences in the abundance and quality of piRNA-producing loci among replicates, and of mutations or polymorphisms that are just present in some replicates.

## Discussion

### Invasion dynamics in *D. erecta*

Here, we introduced the *P*-element into a naive *D. erecta* strain and monitored the ensuing invasions in several replicates using pooled genomic sequencing, long-read sequencing, RNA-seq, small RNA-seq, and GD assays. The current study is thus the most comprehensive investigation of a TE invasion to date.

Overall, we found that the dynamics of the *P*-element invasions in *D. erecta* are very similar to other species. First, the *P*-element has a similar, albeit slightly higher, effective transposition rate in *D. erecta* (*u*′ = 0.217; average of the first 20 generations) (Supplemental Table S2) than in *D. simulans* (*u*′ = 0.15; hot temperature) (Kofler et al. 2018). Second, the positions of the introns of the *P*-element are conserved between *D. melanogaster* and *D. erecta* (Supplemental Figs. S13, S14). Third, similar to other species, IDs of the *P*-element arise rapidly in *D. erecta* (Fig. 5; Black et al. 1987; Kofler et al. 2018, 2022). Fourth, the *P*-element is inducing GD in *D. erecta* similarly as in *D. melanogaster* and *D. simulans* (Kidwell et al. 1977; Hill et al. 2016). Finally, we found that piRNAs are likely not regulating the expression level of the *P*-element but rather the splicing of its introns, especially of IVS3 (Fig. 1E). However, we cannot rule out that piRNAs are just repressing *P*-element expression in the germline stem cells, namely, the cells in which the *P*-element is thought to be active (Moon et al. 2018). Germline stem cells contribute little bulk RNA to RNA samples extracted from ovaries or whole flies, as performed here.

On the whole, we find that the invasion dynamics of the *P*-element, as well as the host response to the invasion, are similar among *D. erecta*, *D. melanogaster*, and *D. simulans*.

### Absence of ping-pong in replicate 2

The level of piRNAs in replicate 2 was lower than in other replicates, owing to the absence of the ping-pong cycle. The ping-pong cycle was inactive for the *P*-element in replicate 2 for at least 67 generations (Figs. 2, 6). It is an important open question as to why the ping-pong cycle is activated in replicates 1 and 4 but not in replicate 2. The presence of ping-pong signatures for other TEs suggests that the ping-pong cycle is functional in replicate 2. One possibility is that the fuel of the ping-pong cycle, that is, sense and antisense transcripts of the *P*-element, is missing in replicate 2. The transcripts identified by RNA-seq may not be exported to the cytoplasm, where transcripts are processed into piRNAs (Czech and Hannon 2016). The presence of sense as well as antisense piRNAs in R2 suggests that at least some transcripts from both strands are processed into piRNAs in replicate 2. However, we cannot exclude the possibility that the amount of antisense (sense) transcripts in the cytoplasm is insufficient for the ping-pong cycle.

**Figure 7.**
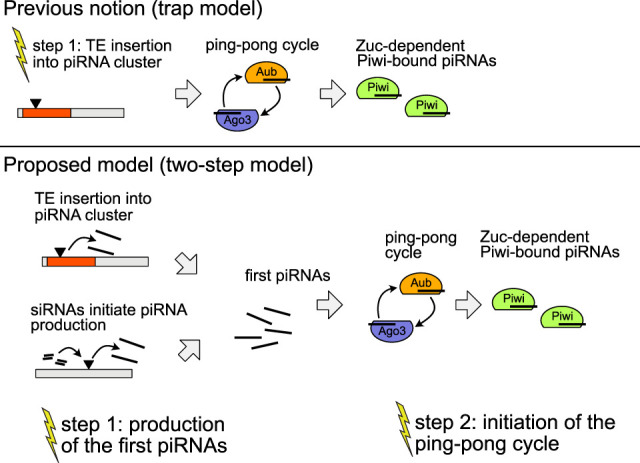
Two steps may be necessary to activate the host defense against an invading TE. According to the prevailing view, that is, the trap model, a TE insertion into a piRNA cluster is sufficient to trigger the host control over an invading TE. In our updated model, the two-step model, we propose that silencing of an invading TE requires two steps: (1) the generation of some first piRNAs and (2) the initiation of the ping-pong cycle. Yellow flashes indicate necessary events for establishing the host control.

We could rule out an influence of the expression of a key gene involved in triggering ping-pong, *lok* (*Chk2*) (Moon et al. 2018); the abundance of antisense transcripts against the *P*-element; the viral load (Roy et al. 2020); and differences in expressions of *D. erecta* transcripts. With the reciprocal crosses among replicates, we could further preclude an influence of the genomic composition, as the genomes are largely identical among the reciprocal crosses. As a consequence, we can also rule out any influence of particularly potent cluster insertions that may just be present in replicates 1 or 4 but not in replicate 2. Furthermore, the reciprocal crosses also rule out any influence of the prominent ID found solely in replicate 2 (i.e., the *EP*-element) and of mutations or polymorphism that may be specific to some replicate. These crosses instead suggest that some maternally transmitted factor (possibly small RNAs) is responsible for the absence of ping-pong in replicate 2 (Fig. 6). Maternally deposited factors are crucial for the ping-pong cycle. It has been shown that maternally deposited piRNAs initiate the ping-pong cycle in the next generation (Le Thomas et al. 2014). This raises the important question as to which events trigger the ping-pong cycle de novo for a newly invading TE, in which no or few piRNAs complementary to the TE are maternally deposited. Our data suggest that neither insertions in piRNA clusters nor siRNAs are sufficient to initiate the ping-pong cycle. However, insertions in piRNA clusters and/or siRNAs may be necessary preconditions for triggering the ping-pong cycle. It is thus feasible that the host defense is established in two steps (Fig. 7). First, insertions into piRNA clusters (or siRNAs) may trigger the emergence of some piRNAs complementary to an invading TE. Second, the ping-pong cycle is activated. Activation of the host defense by two independent events may help to minimize deleterious effects arising from the host defense against TEs such as the silencing of genes as off-targets (Miller et al. 2023). It is, however, unclear what triggers the activation of the ping-pong cycle. Also, an alternative hypothesis could account for the silencing of TEs (Supplemental Text S1).

### Limitations of this study

One limitation of our work is that, due to the COVID-19 outbreak, we could not carefully control the size of the experimental populations following generation 34 (i.e., after the host defense was established in replicates 1 and 4). This could have resulted in unnoticed fluctuations of the population size, which might have led to the amplification of stochastic events occurring in some replicates or differences in the strength of selection (positive or negative) in some replicates. However, because of the reciprocal crosses among the replicates, we could rule out that failure to establish ping-pong in replicate 2 is owing to some mutations or polymorphisms specific to a replicate. These crosses showed that failure to establish ping-pong is owing to a maternally transmitted component, likely small RNAs. We can, however, not rule out that other maternally transmitted epigenetic factors play a role. Finally, it is feasible that *P*-element siRNAs observed in the small RNA data from total ovaries are just derived from the somatic follicle cells (Rozhkov et al. 2010).

### Consequences for natural populations

Here we showed that a *P*-element invasion could escape host control in experimental populations. Notably, our data suggest that the failed host defense was not limited to a few individuals but occurred in all examined individuals of a population (estimates of the ping-pong signatures were based on the RNA of 10–30 flies). This raises the question as to whether such a failed host defense could also happen in natural populations. The invasion dynamics in natural populations may be different than in our experimental populations. Whereas experimental populations are largely panmictic, more population subdivision is expected in natural populations. Migration between populations could help to spread the host defense to populations with an unchecked TE invasion. Also, the larger population size of natural populations might reduce the impact of stochastic events that could have contributed to failure to initiate the ping-pong cycle in replicate 2. An unchecked invasion, as described here, could thus solely be a threat to isolated small populations or largely panmictic species. A related open question is how often TE invasions escape host control. In our three experimental populations, host defenses were not established in a single replicate. It is thus likely that an unchecked TE invasion is a rare event. An important question is the consequences of an unchecked invasion to natural populations. The TE will likely attain unusually high copy numbers in such (local) populations without host control. It is feasible that such an unchecked proliferation of TEs can lead to reduced fecundity and possibly even to extinction of populations. For example, *P*-element invasions led to extinction of several experimental populations in *D. melanogaster* (Wang et al. 2023). This raises the possibility that TE invasions could drive natural populations, or possibly even species, to extinction. TE invasions could be much more abundant than previously assumed. For example, at least eight different TEs invaded the genome of *D. melanogaster* during the past 200 years (Scarpa et al. 2023). TE invasions could thus pose a persistent threat to genome integrity of organisms. This problem could be especially severe when species are already stressed, for example, owing to climate change, or when an environmental change increases the activity of the invading TE. Rising global temperatures will, for example, increase the activity of the *P*-element (Moon et al. 2018).

## Methods

A detailed description of the Methods is available as Supplemental Text S2.

### Strains and transformation

We introduced the *P*-element into the *D. erecta* strain 14021-0224.01 using microinjection. Injections were performed by Rainbow Transgenic Flies (https://www.rainbowgene.com/). We obtained seven lines having the *P*-element by crossing the transformed adults (two males and three females). The transformed lines were maintained at 20°C for three generations before the experimental populations were set up.

### Experimental populations

To establish the experimental populations, we crossed five males from five *P*-element lines with five naive virgin females and allowed them to mate for 3 d. After mating, we mixed the 50 flies from the crosses with 200 naive *D. erecta* flies. We maintained three replicates of the experimental populations with a size of *N* = 250 for 50 generations at 25°C using nonoverlapping generations.

### Short-read sequencing of genomic DNA

At about each 10th generation, we sequenced pools of 60 flies using Illumina 2- × 125-bp reads. Individual flies at generation 42 were sequenced by BGI using 2- × 150-bp reads (BGI Tech Solutions).

### Abundance and diversity of TE insertions

We estimated the abundance and diversity of the *P*-element with DeviaTE (Weilguny and Kofler 2019). The short reads were trimmed to a size of 125 bp and aligned with BWA-SW (v0.7.17) (Li and Durbin 2009) to the consensus sequences of TEs in *D. melanogaster* (which contains the *P*-element) (Quesneville et al. 2005) and three single copy genes of *D. erecta*: *tj*, *RpL32*, and *rhi* (from FlyBase release 2017_05).

To identify the genomic position and population frequency of *P*-element insertions, we used PoPoolationTE2 (v1.10.04) (Kofler et al. 2016) and reads trimmed to a size of 75 bp at the 3′-end. The number of reads mapping to the *P*-element (rpm) was also estimated with PopoolationTE2.

### RNA sequencing

We sequenced RNA either from whole female flies or from ovaries. We used 30 flies for the extractions of all samples except generation 10, in which solely 10–15 flies were used. Small RNA and RNA samples were sequenced by Fasteris. The RNA samples were treated with DNase I and poly(A)-selected before they were sequenced on the Illumina NovoSeq machine, with a read length of 2 × 100 bp.

### Analysis of small RNA data

Adaptor sequences were removed with cutadapt (v2.6) (Martin 2011), and reads with a length between 18 and 35 nt were retained. We aligned small RNA reads to the *D. erecta* tRNAs, miRNAs, mRNAs, snRNAs, snoRNAs, rRNAs, and the consensus sequences of TEs from *D. melanogaster* using NovoAlign (v3.09.00; http://www.novocraft.com/). The abundance of different small RNAs, the distribution of piRNAs within the *P*-element, the length distribution of the piRNAs, and the ping-pong signal were computed using previously described Python scripts (Kofler et al. 2018). The motifs of small RNAs were computed with a novel script (smallRNA-U-bias.py) and the R-package ggseqlogo (Wagih 2017; R Core Team 2022).

To identify piRNA clusters, we mapped the small RNA data from naive ovaries to a long-read assembly of *D. erecta* (Kim et al. 2021) with NovoAlign (see above), counted unambiguously mapped reads, and identified piRNA clusters with a previously described algorithm (Kofler et al. 2018, 2022). Clusters <2000 bp were ignored. We estimated the abundance of virus-derived reads in the small RNA libraries by aligning the small RNA data to a collection of *Drosophila* viruses (Obbard 2018; Wallace et al. 2021) with NovoAlign (v3.03.02).

### Analysis of expression data

We aligned the RNA data with gsnap (version 2014-10-22) (Wu and Nacu 2010) to the transcripts of *D. erecta* (r1.3; FlyBase) and the consensus sequences of TEs in *D. melanogaster* (Quesneville et al. 2005). The coverage and the splicing level of the *P*-element were visualized in R. Significant differences in expression levels were identified with edgeR based on the raw counts (v3.38.1; glmQLFit test) (Robinson et al. 2010; R Core Team 2022). Volcano plots were generated in ggplot2 (Wickham 2016). The orthologous sequence of *lok* was identified by aligning *D. melanogaster* genes to *D. erecta* transcripts with BWA-SW (v0.7.17) (Li and Durbin 2009).

### Long-read sequencing

For Oxford Nanopore sequencing we used 60 flies, the ligation sequencing kit SQK-LSK109, and R9 flow cells. The long reads were aligned with minimap2 (v2.10-r761) (Li 2018) to the long-read assembly of *D. erecta* and the sequence of the *P*-element (see above). We identified reads supporting *P*-element insertions (Pele-insertion-finder.py), filtered reads for insertions in piRNA clusters (find-lr-bedinsertion.py), and identified *P*-element insertions in piRNA clusters by grouping reads supporting a cluster insertion at similar positions (group-cluster-insertions.py). To estimate the population frequency of different *P*-element variants, we identified the location of full-length *P*-element and *EP*-element insertions from long reads and used the frequency estimates provided by PoPoolationTE2 (see above). For each *P*-element insertion, we used the frequency estimate of the nearest insertions.

### Crosses among replicates

At generations 67–70, we performed reciprocal crosses between flies from replicate 2 with flies from replicates 1 and 4. We crossed 15 virgin females from replicate 2 with males from replicates 1 and 4 and vice versa. For each cross, we set up three subreplicates. The parental females and the F_1_ females were used for RNA extraction and sequencing.

## Data access

All raw and processed sequencing data generated in this study have been submitted to the NCBI BioProject database (https://www.ncbi.nlm.nih.gov/bioproject/) under accession number PRJNA916392. All scripts used in this work have been submitted to SourceForge (https://sourceforge.net/) under the project te-tools (https://sourceforge.net/projects/te-tools/; directories ”ere” and ”longread”) and are available as Supplemental Code.

## Supplementary Material

Supplement 1

Supplement 2

Supplement 3

Supplement 4

Supplement 5

Supplement 6

Supplement 7

Supplement 8

Supplement 9

Supplement 10

Supplement 11

Supplement 12

Supplement 13

Supplement 14

Supplement 15

Supplement 16

Supplement 17

Supplement 18

Supplement 19

Supplement 20

Supplement 21

Supplement 22

Supplement 23

Supplement 24

Supplement 25

Supplement 26

Supplement 27

Supplement 28

Supplement 29

Supplement 30

Supplement 31

Supplement 32

Supplement 33

Supplement 34

Supplement 35

Supplement 36

Supplement 37

Supplement 38

Supplement 39

Supplement 40

Supplement 41

Supplement 42

Supplement 43

Supplement 44

Supplement 45
