# Supplementary material for: Experimentally evolving Drosophila erecta populations may fail to establish an effective piRNA-based host defense against invading P-elements
Source: Genome Res. 2024 Mar;34(3):410–25. doi: 10.1101/gr.278706.123 (PMC11067887; doi:10.1101/gr.278706.123)
Supplement: Supplement 1 [file Supplementary_Fig_S1.pdf]

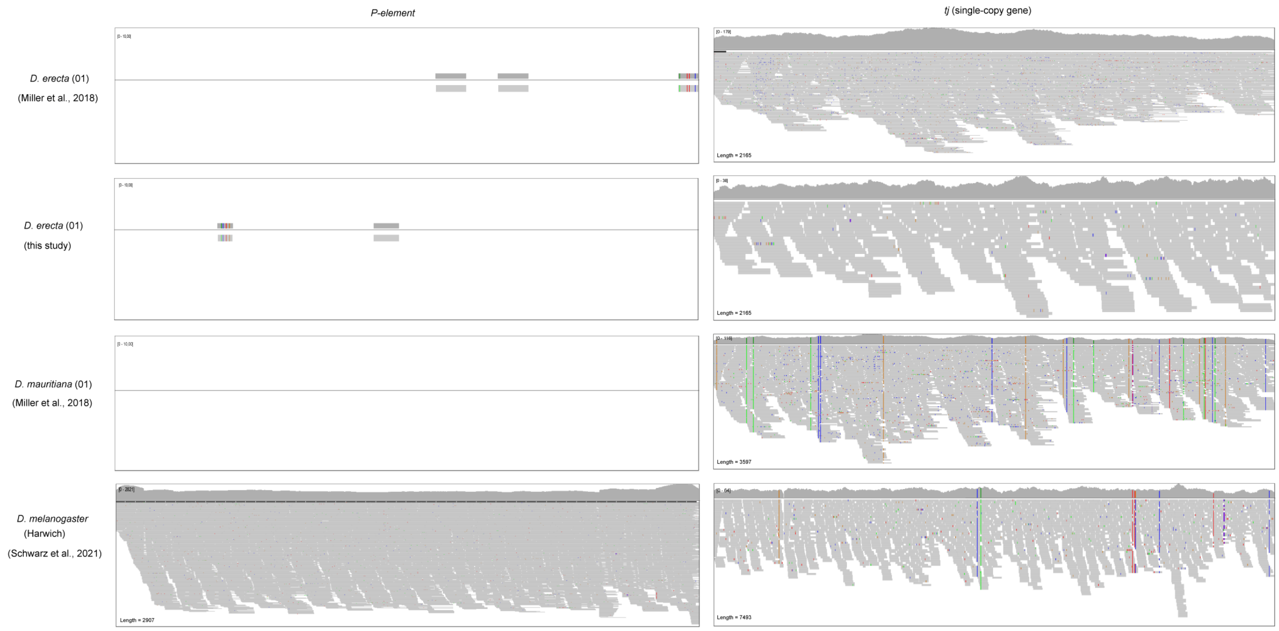

Figure 1: Abundance of reads aligning to the *P-element* and the single-copy gene *tj* in *D. erecta* (strain 01), *D. mauritiana* (strain 01) and *D. melanogaster* (Harwich). Previous works showed that the *P-element* is present in the *D. melanogaster* strain Harwich but absent in *D. mauritiana* [Kofler et al., 2015, Brookfield et al., 1984, Srivastav et al., 2019]. In agreement with this, many reads align to the *P-element* in *D. melanogaster* but none in *D. mauritiana*. Although many reads align to the single-copy gene solely 2-3 reads align to the *P-element* in *D. erecta*, suggesting that the *P-element* is absent in *D. erecta*.
