# Supplementary material for: Experimentally evolving Drosophila erecta populations may fail to establish an effective piRNA-based host defense against invading P-elements
Source: Genome Res. 2024 Mar;34(3):410–25. doi: 10.1101/gr.278706.123 (PMC11067887; doi:10.1101/gr.278706.123)
Supplement: Supplement 2 [file Supplementary_Fig_S2.pdf]

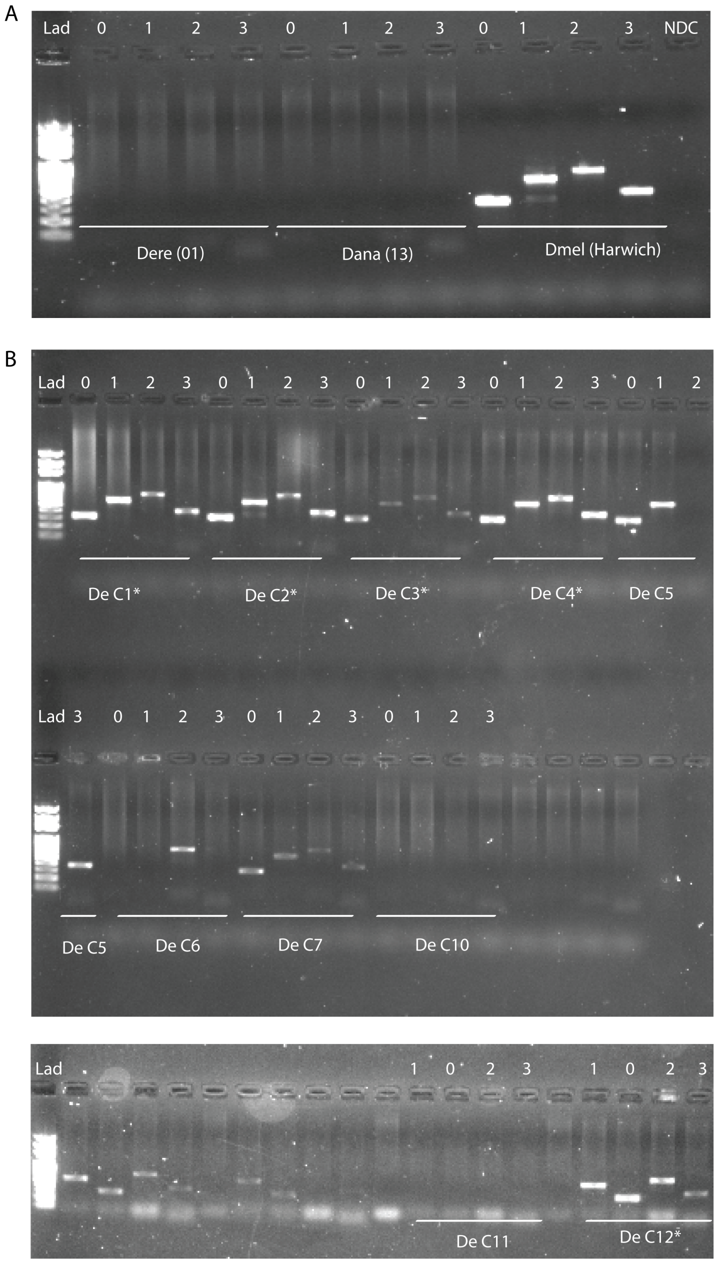

Figure 2: Presence of the *P-element* in different *D. erecta* lines. We tested each line (species) with four different primer pairs (number on top), where a primer pair was designed for each of the four ORFs of the *P-element* [Hill et al., 2016]. A) The *P-element* is absent in the *D. erecta* strain 01. The *D. melanogaster* strain Harwich was used as a positive control and *D. ananassae* as negative control [Daniels et al., 1990, Srivastav et al., 2019]. B) Presence of the *P-element* in 12 transformed *D. erecta* lines. The plasmid ppi25.1 was microinjected into naive *D. erecta* 01 embryos. Surviving G0 flies were mated and 12 lines were established. The lines used for setting up the experimental populations are marked with a star (*).
