# Supplementary material for: Experimentally evolving Drosophila erecta populations may fail to establish an effective piRNA-based host defense against invading P-elements
Source: Genome Res. 2024 Mar;34(3):410–25. doi: 10.1101/gr.278706.123 (PMC11067887; doi:10.1101/gr.278706.123)
Supplement: Supplement 3 [file Supplementary_Fig_S3.pdf]

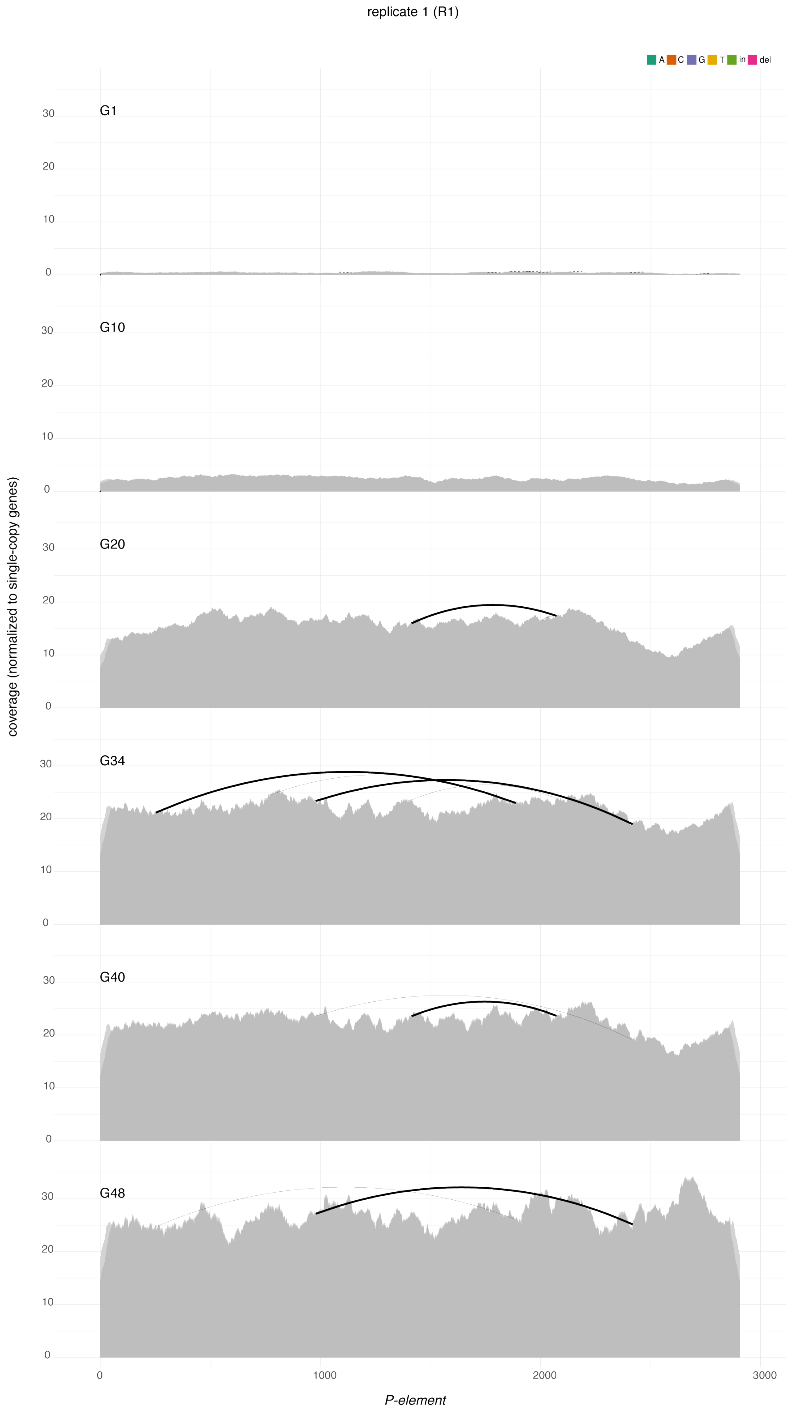


Figure 3: Abundance and diversity of the *P-element* during the invasion in replicate 1 visualized with DeviaTE [Weilguny and Kofler, 2019]. Single-nucleotide polymorphisms (SNPs) and small internal deletions (indels) are shown as colored lines. The absence of colored lines highlights that the *P-element* has no SNPs or solely SNPs segregating at a very low frequency. Large internal deletions are shown as black arcs.
