# Supplementary figures and images for: Experimentally evolving Drosophila erecta populations may fail to establish an effective piRNA-based host defense against invading P-elements
Source: Genome Res. 2024 Mar;34(3):410–25. doi: 10.1101/gr.278706.123 (PMC11067887; doi:10.1101/gr.278706.123)

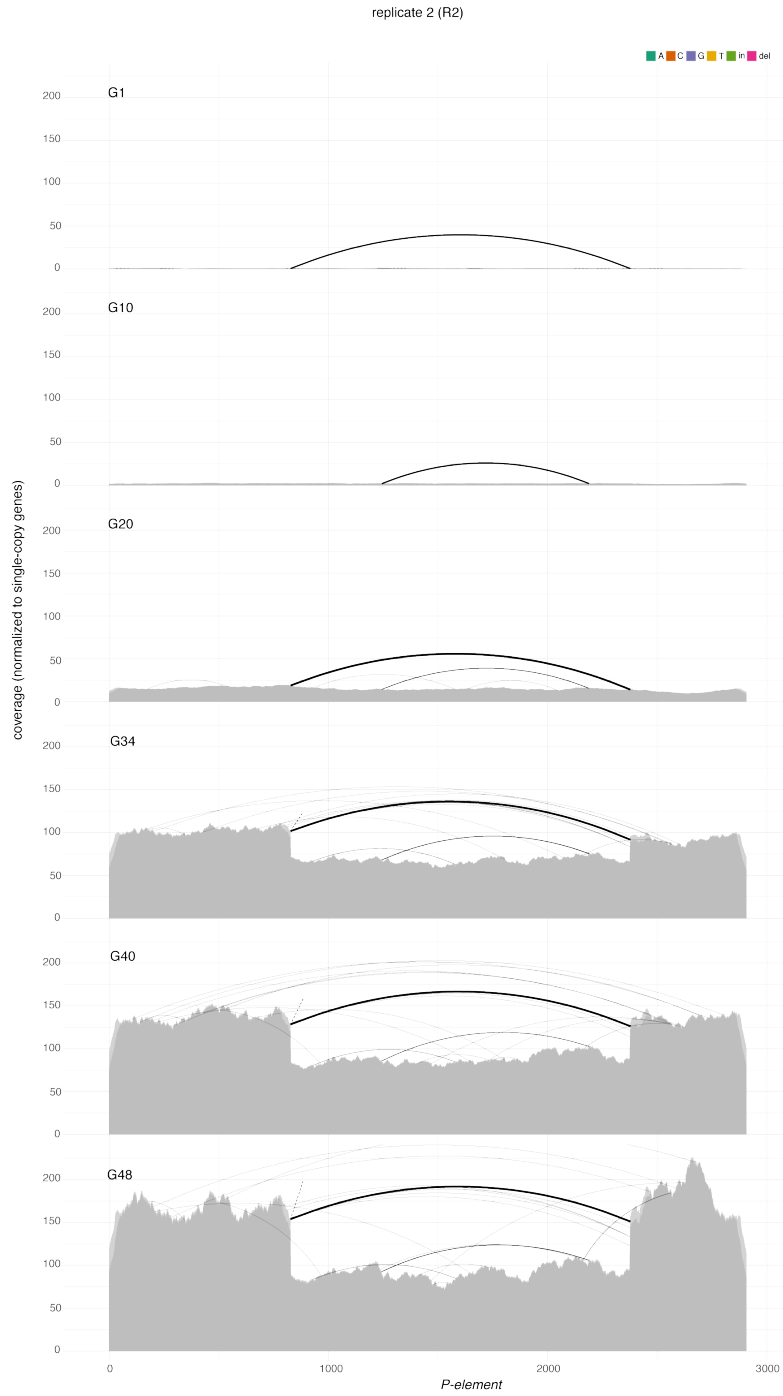

Figure 4: Abundance and diversity of the *P-element* during the invasion in replicate 2.

Supplement: Supplement 4 [file Supplementary_Fig_S4.pdf]

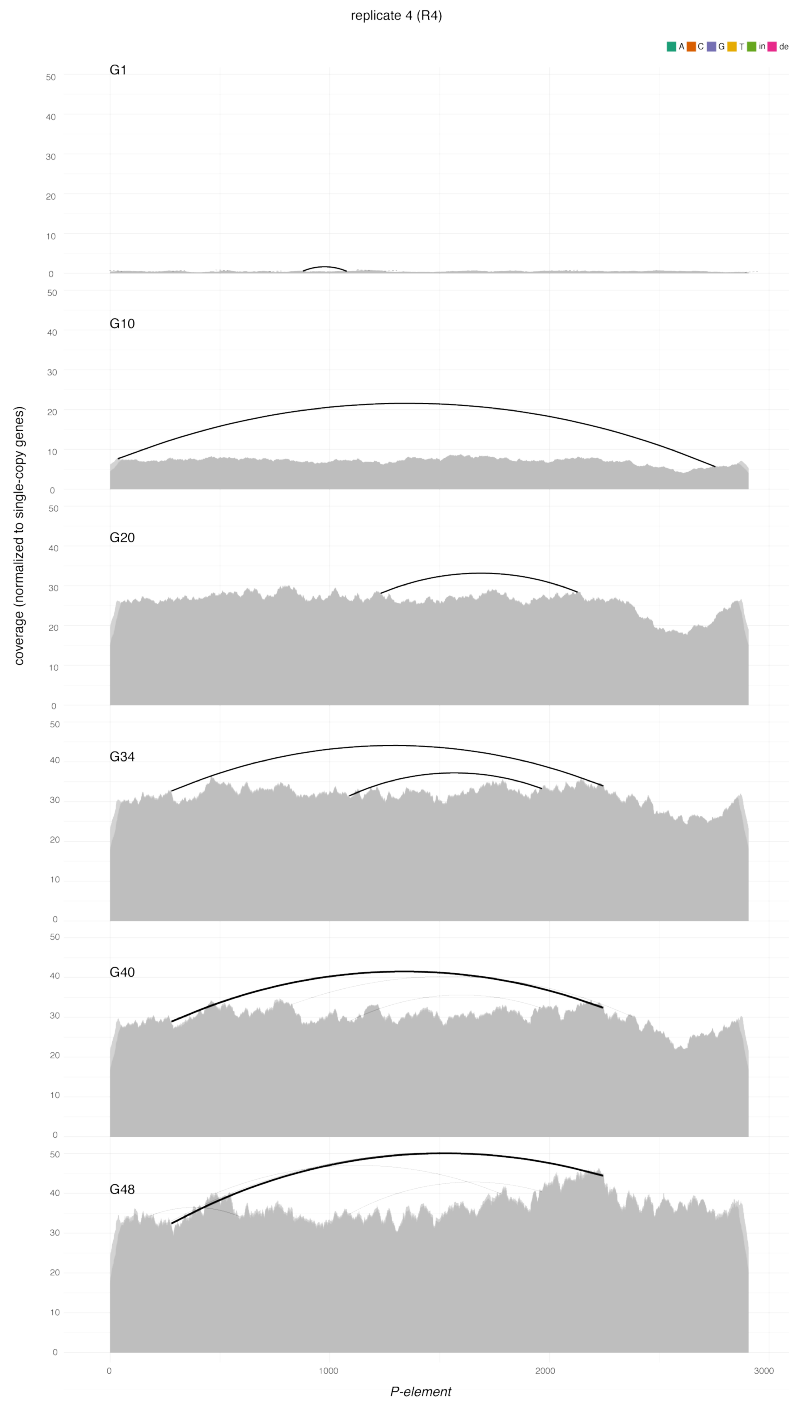

Figure 5: Abundance and diversity of the *P-element* during the invasion in replicate 4.

Supplement: Supplement 5 [file Supplementary_Fig_S5.pdf]

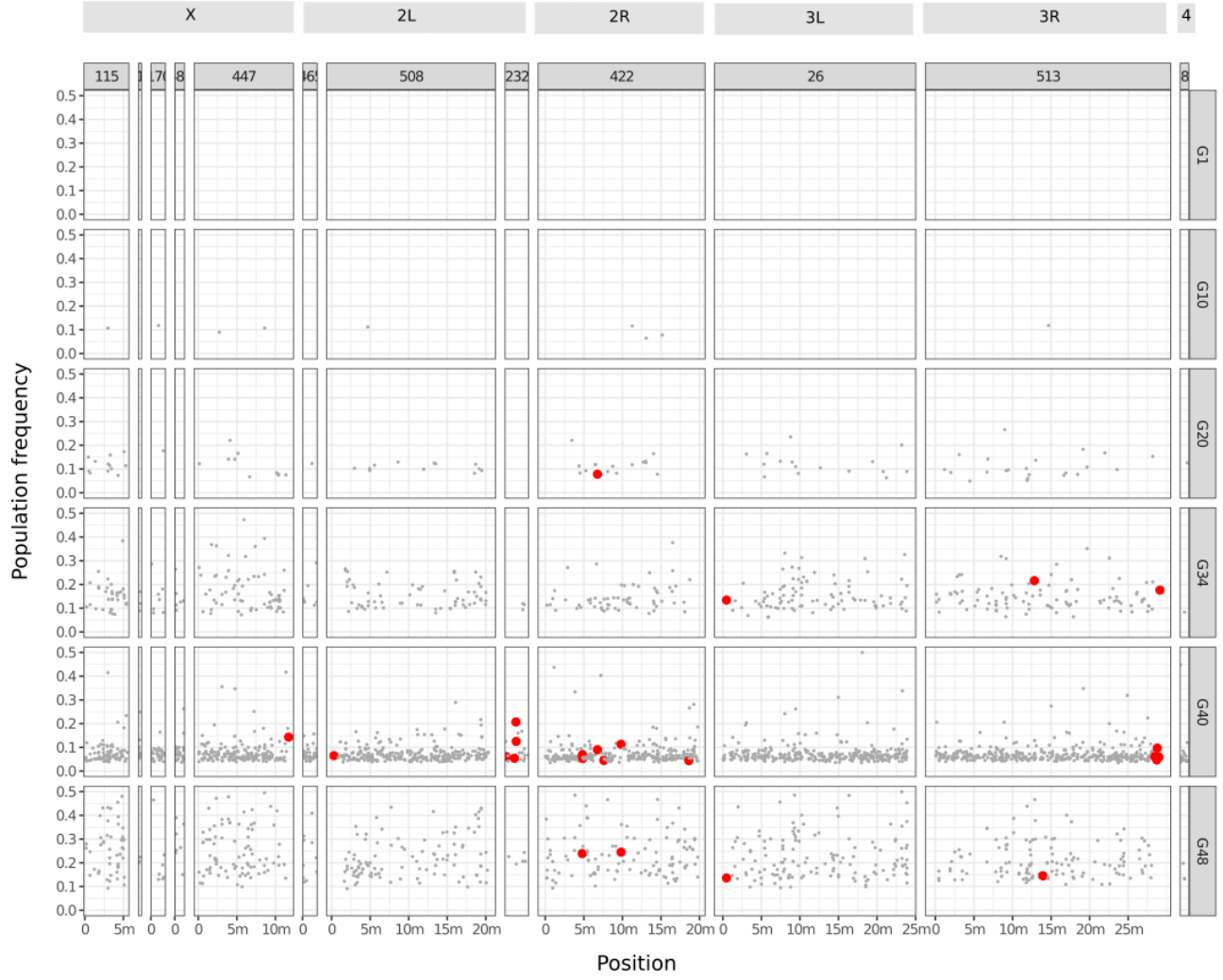

Figure 20: Position and population frequency of *P-element* insertions in replicate 2.

Supplement: Supplement 20 [file Supplementary_Fig_S20.pdf]

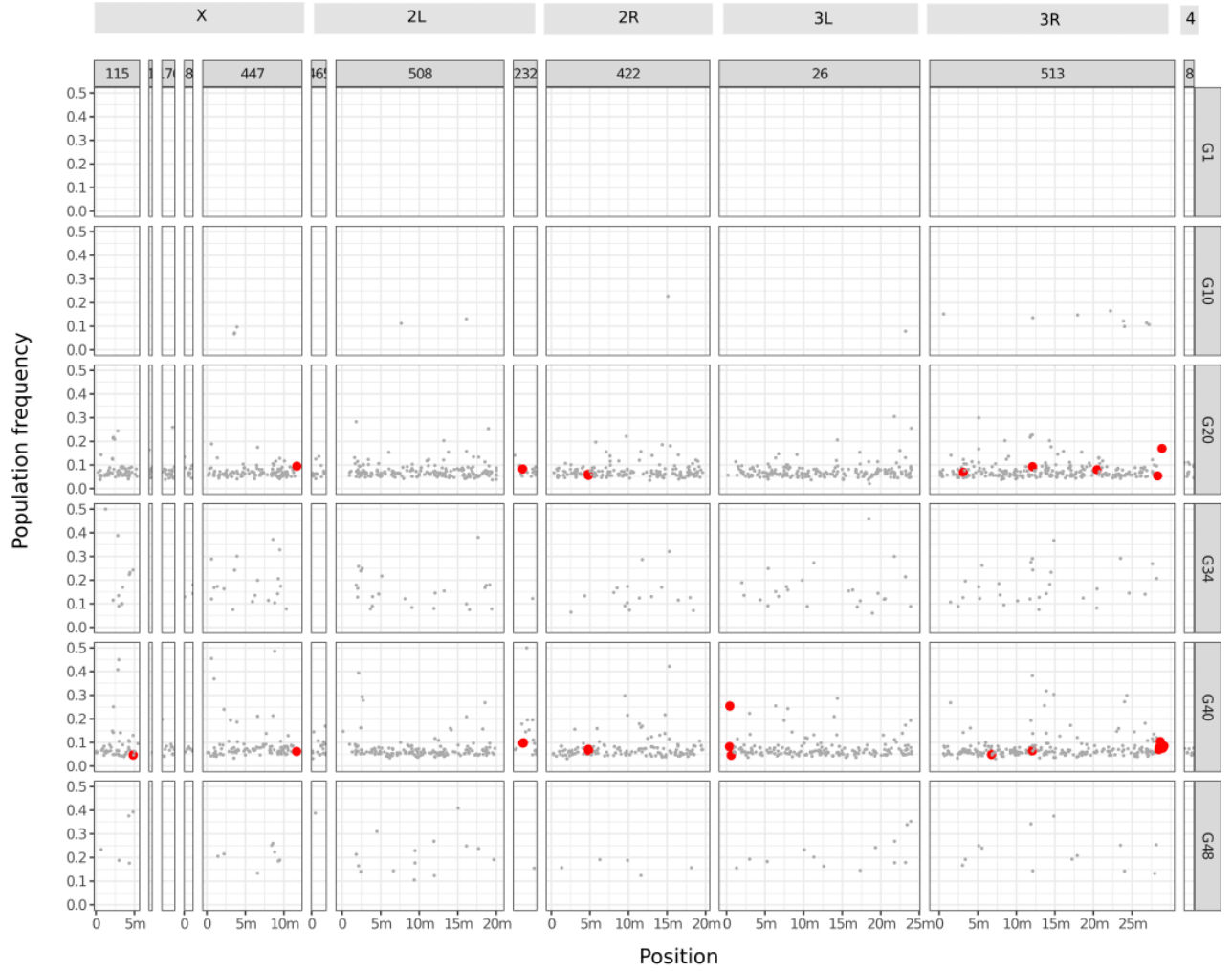

Figure 21: Position and population frequency of *P-element* insertions in replicate 4.

Supplement: Supplement 21 [file Supplementary_Fig_S21.pdf]
