# Supplementary material for: Experimentally evolving Drosophila erecta populations may fail to establish an effective piRNA-based host defense against invading P-elements
Source: Genome Res. 2024 Mar;34(3):410–25. doi: 10.1101/gr.278706.123 (PMC11067887; doi:10.1101/gr.278706.123)
Supplement: Supplement 6 [file Supplementary_Fig_S6.pdf]

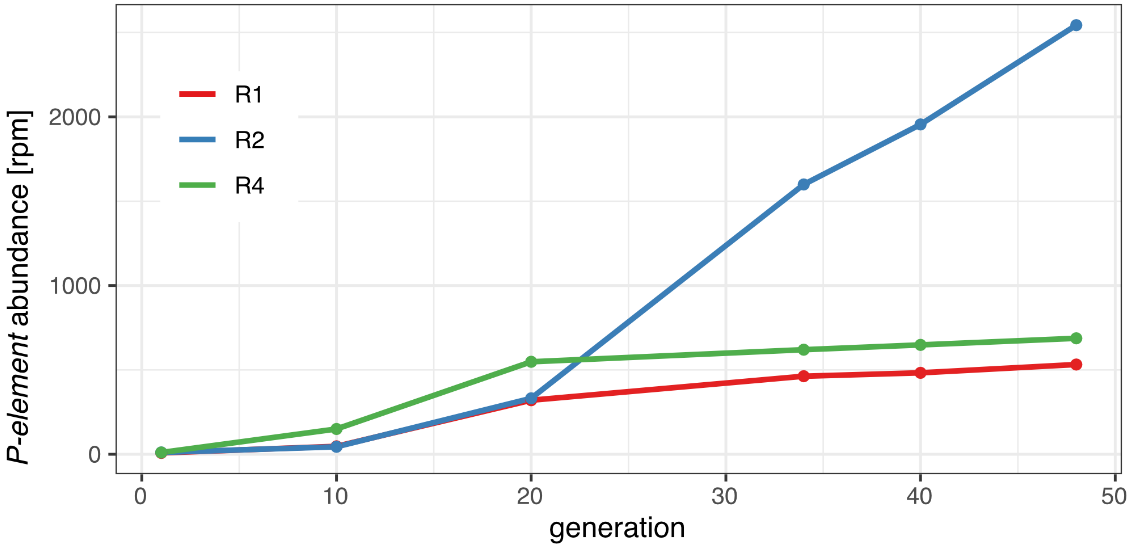


Figure 6: Abundance of *P-element* insertions during the invasion in reads mapping to the *P-element* out of a million reads (reader per million; rpm). Data are shown for three replicates (R1, R2, R4).
