# Supplementary material for: Experimentally evolving Drosophila erecta populations may fail to establish an effective piRNA-based host defense against invading P-elements
Source: Genome Res. 2024 Mar;34(3):410–25. doi: 10.1101/gr.278706.123 (PMC11067887; doi:10.1101/gr.278706.123)
Supplement: Supplement 7 [file Supplementary_Fig_S7.pdf]

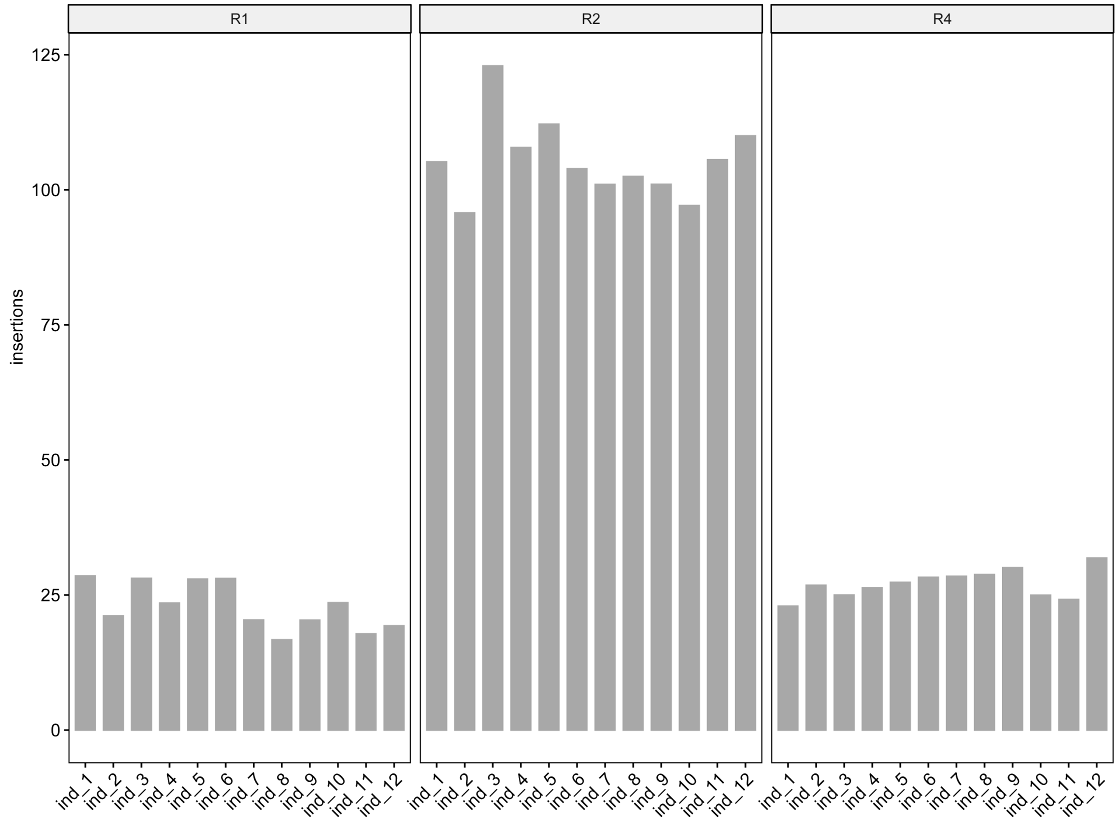

Figure 7: Abundance of *P-element* insertions in 12 individual flies sampled at generation 42 from each replicate.
