# Supplementary material for: Experimentally evolving Drosophila erecta populations may fail to establish an effective piRNA-based host defense against invading P-elements
Source: Genome Res. 2024 Mar;34(3):410–25. doi: 10.1101/gr.278706.123 (PMC11067887; doi:10.1101/gr.278706.123)
Supplement: Supplement 8 [file Supplementary_Fig_S8.pdf]

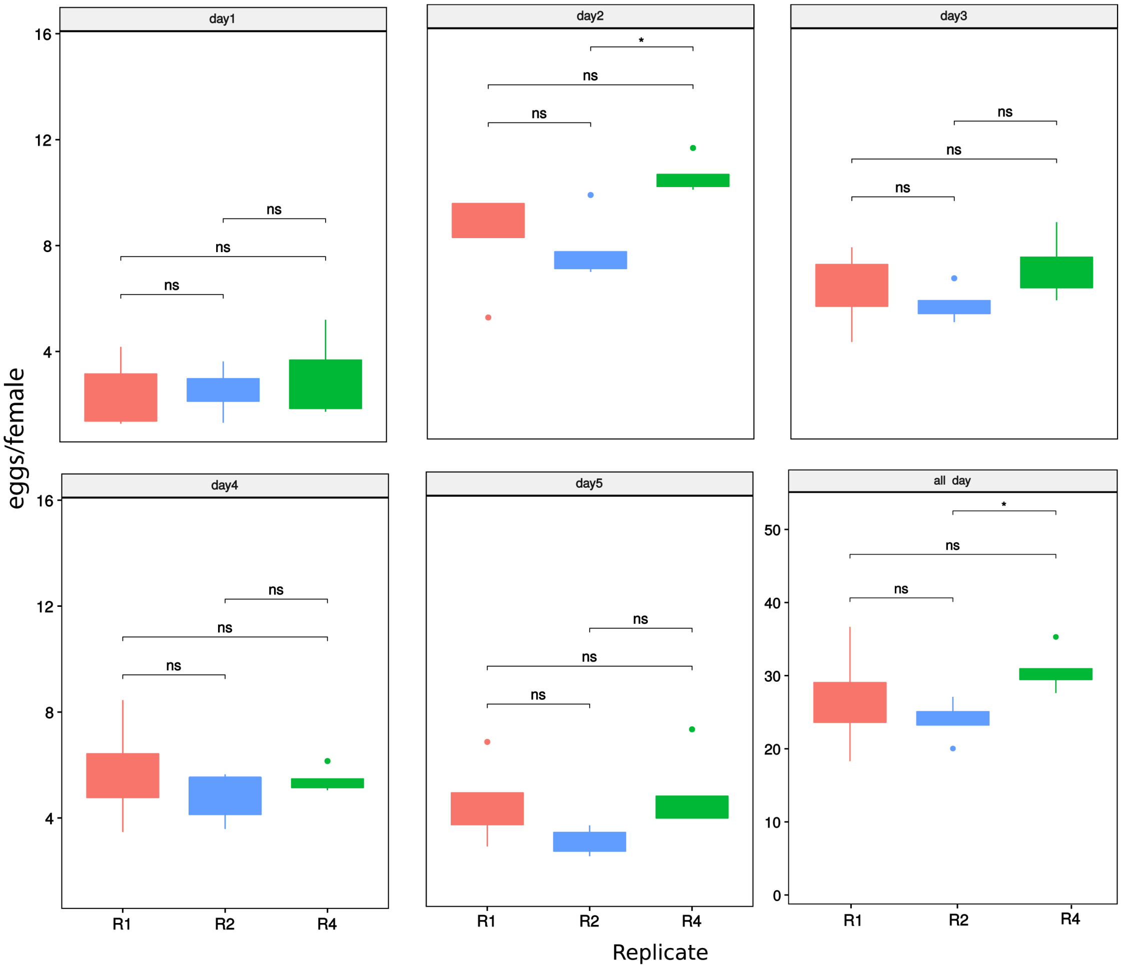

Figure 8: Fecundity in the different replicates at generation 88. We estimated the average fecundity per fly, by counting the number of eggs laid by 29-40 female at 5 successive days (starting with 5 day old flies). One generation of density control was performed at generation 88. Significance was computed with t-tests.
