# Supplementary material for: Experimentally evolving Drosophila erecta populations may fail to establish an effective piRNA-based host defense against invading P-elements
Source: Genome Res. 2024 Mar;34(3):410–25. doi: 10.1101/gr.278706.123 (PMC11067887; doi:10.1101/gr.278706.123)
Supplement: Supplement 9 [file Supplementary_Fig_S9.pdf]

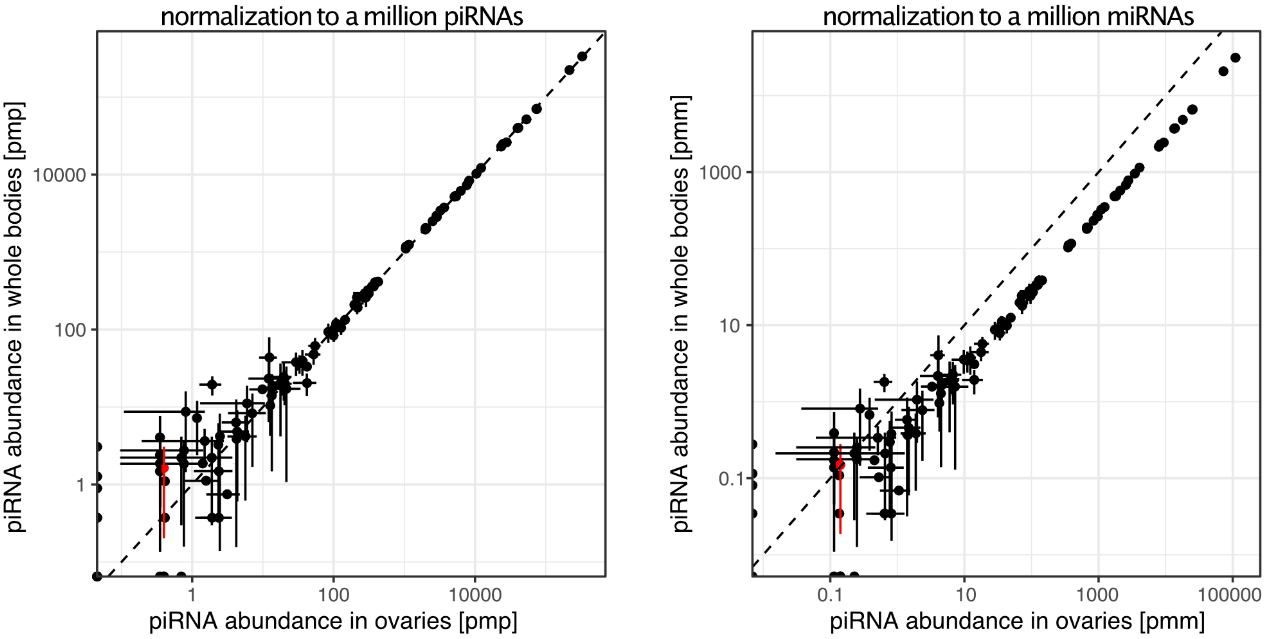

Figure 9: Effect of the normalization method on the piRNA abundance in ovaries and whole female flies. The abundance of piRNAs for each TE family (each dot is a TE family) was either normalized to a million piRNAs (left) or to a million miRNAs (right). We sequenced three replicates from ovaries and whole female flies of naive *D. erecta* flies (not having the *P-element*). For each replicate RNA was extracted from different flies. The error bars show the standard deviation among the three replicates and the dashed lines indicate the diagonal, i.e. the perfect correlation. The abundance of piRNAs complementary to the *P-element* is shown in red. Note that sequencing of small RNAs from ovaries and whole female flies yield similar results if the normalization to a million piRNAs is used. pmp piRNAs per million piRNAs, pmm piRNAs per million miRNAs
