# Supplementary material for: Experimentally evolving Drosophila erecta populations may fail to establish an effective piRNA-based host defense against invading P-elements
Source: Genome Res. 2024 Mar;34(3):410–25. doi: 10.1101/gr.278706.123 (PMC11067887; doi:10.1101/gr.278706.123)
Supplement: Supplement 10 [file Supplementary_Fig_S10.pdf]

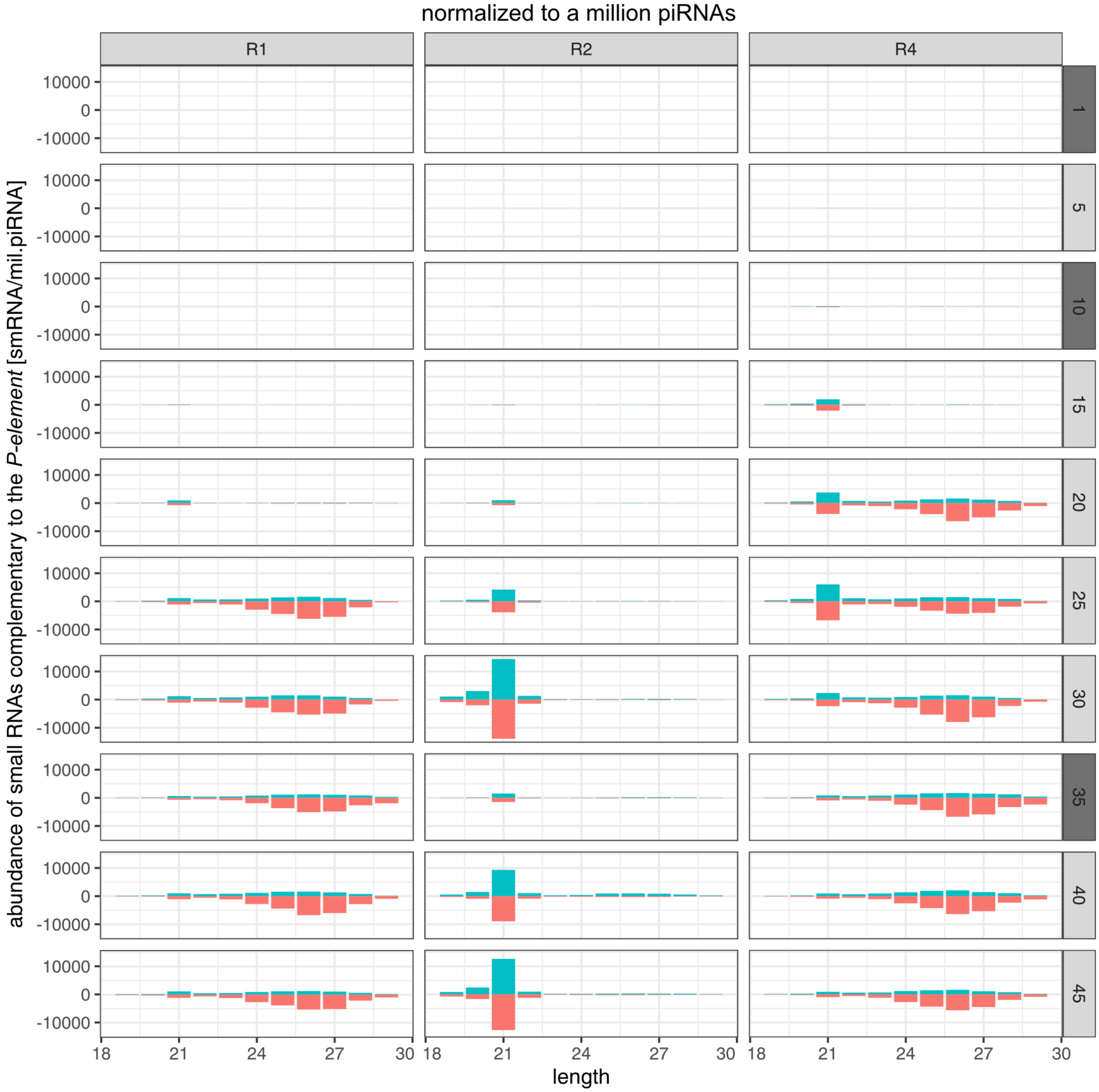

Figure 10: Length distribution of small RNAs mapping to the *P-element*. The abundance of small RNAs was normalized to 1 million piRNAs (smRNAs/mil.piRNA). Data are shown for three replicates (top panels) and several generations during the invasion (right panel). Sense RNAs are on the positive y-axis and antisense RNAs on the negative y-axis. small RNA data were either generated for whole bodies of female flies (light grey panels) or ovaries (dark grey panels).
