# Supplementary material for: Experimentally evolving Drosophila erecta populations may fail to establish an effective piRNA-based host defense against invading P-elements
Source: Genome Res. 2024 Mar;34(3):410–25. doi: 10.1101/gr.278706.123 (PMC11067887; doi:10.1101/gr.278706.123)
Supplement: Supplement 12 [file Supplementary_Fig_S12.pdf]

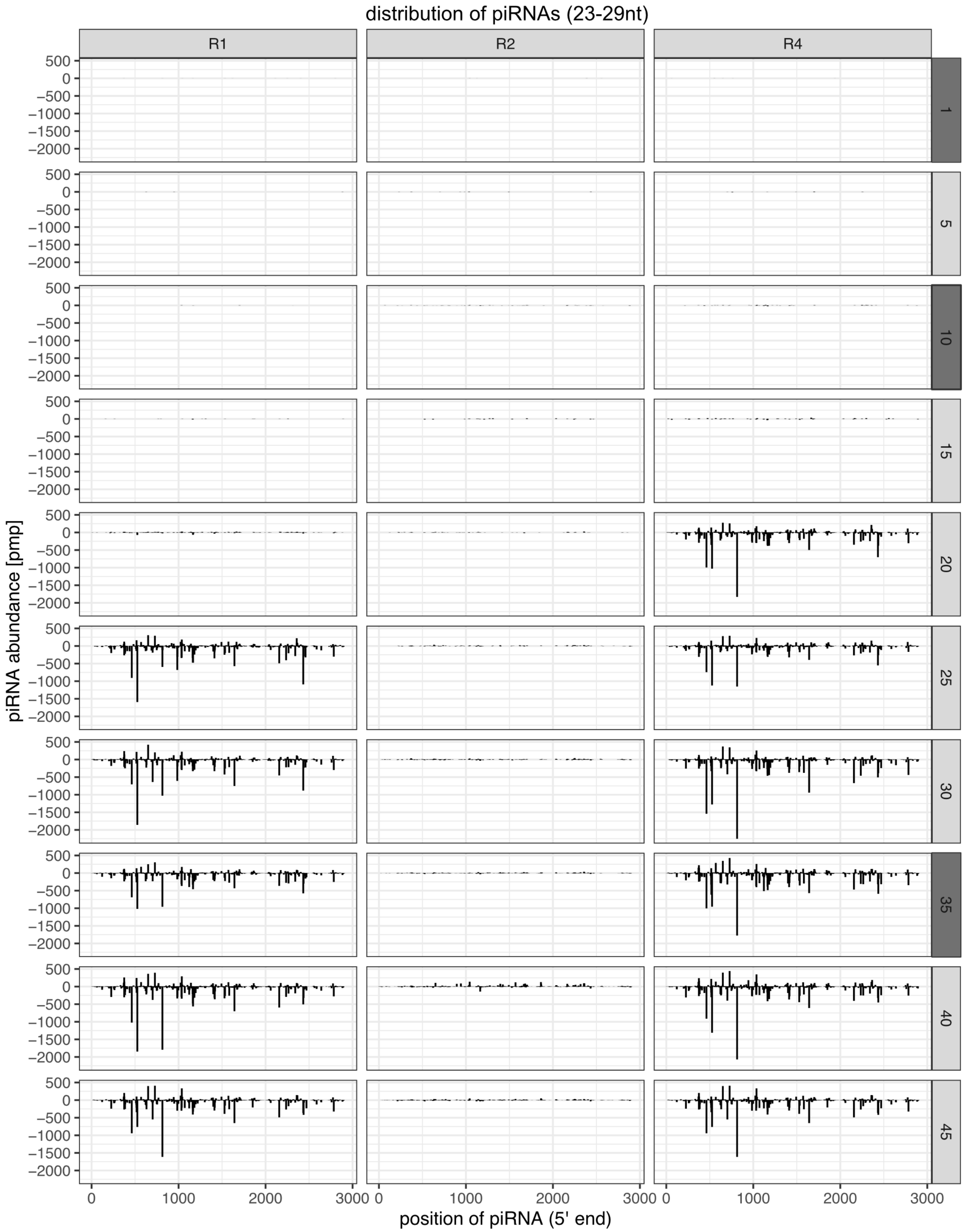

Figure 12: Distribution of piRNAs (23-29nt) along the *P-element*. Only the 5' positions of piRNAs are shown and the piRNA abundance is normalized to one million piRNAs. Replicates are at the top panel and the generations at the right panel. Sense piRNAs are shown on the positive y-axis and antisense piRNAs on the negative y-axis. small RNA data were either generated for whole bodies of female flies (light grey panels) or ovaries (dark grey panels).
