# Supplementary material for: Experimentally evolving Drosophila erecta populations may fail to establish an effective piRNA-based host defense against invading P-elements
Source: Genome Res. 2024 Mar;34(3):410–25. doi: 10.1101/gr.278706.123 (PMC11067887; doi:10.1101/gr.278706.123)
Supplement: Supplement 13 [file Supplementary_Fig_S13.pdf]

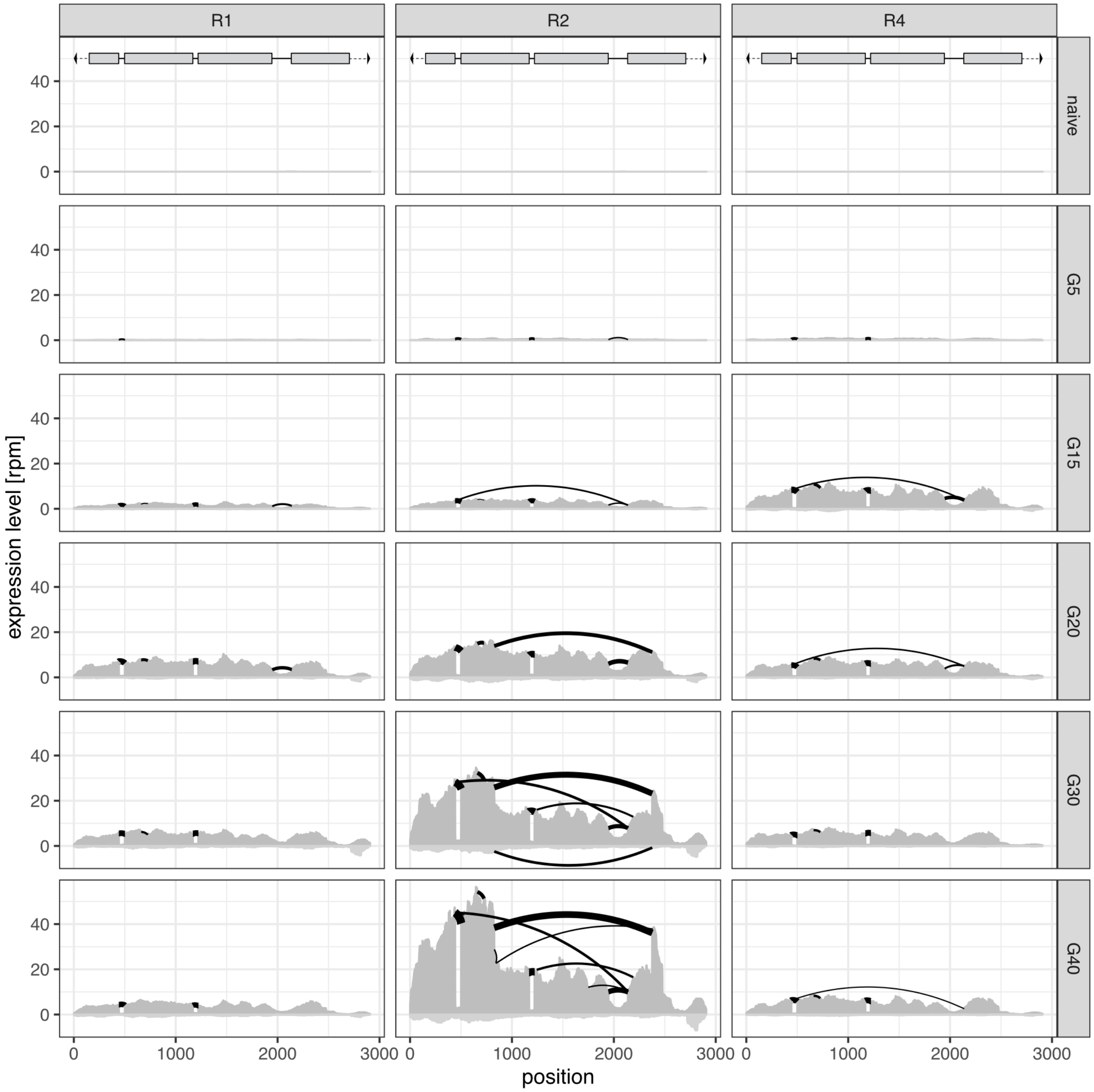

Figure 13: Sashimi plots showing the expression level and splicing of the *P-element* in female flies (whole body). Data are shown for different replicates (top panel) and generations during the invasion (right panel). Both the expression and splicing level (width of the black arcs) were normalized to a million mapped reads. Sense expression and gaps of sense transcripts (splicing or internal deletions) are shown on the positive y-axis whereas antisense expression and gaps of antisense transcripts are shown on the negative y-axis. The structure of the *P-element* is shown at the top, where terminal inverted repeats (TIRs) (black triangles), the four exons (grey rectangles) and introns (black lines) are shown.
