# Supplementary material for: Experimentally evolving Drosophila erecta populations may fail to establish an effective piRNA-based host defense against invading P-elements
Source: Genome Res. 2024 Mar;34(3):410–25. doi: 10.1101/gr.278706.123 (PMC11067887; doi:10.1101/gr.278706.123)
Supplement: Supplement 14 [file Supplementary_Fig_S14.pdf]

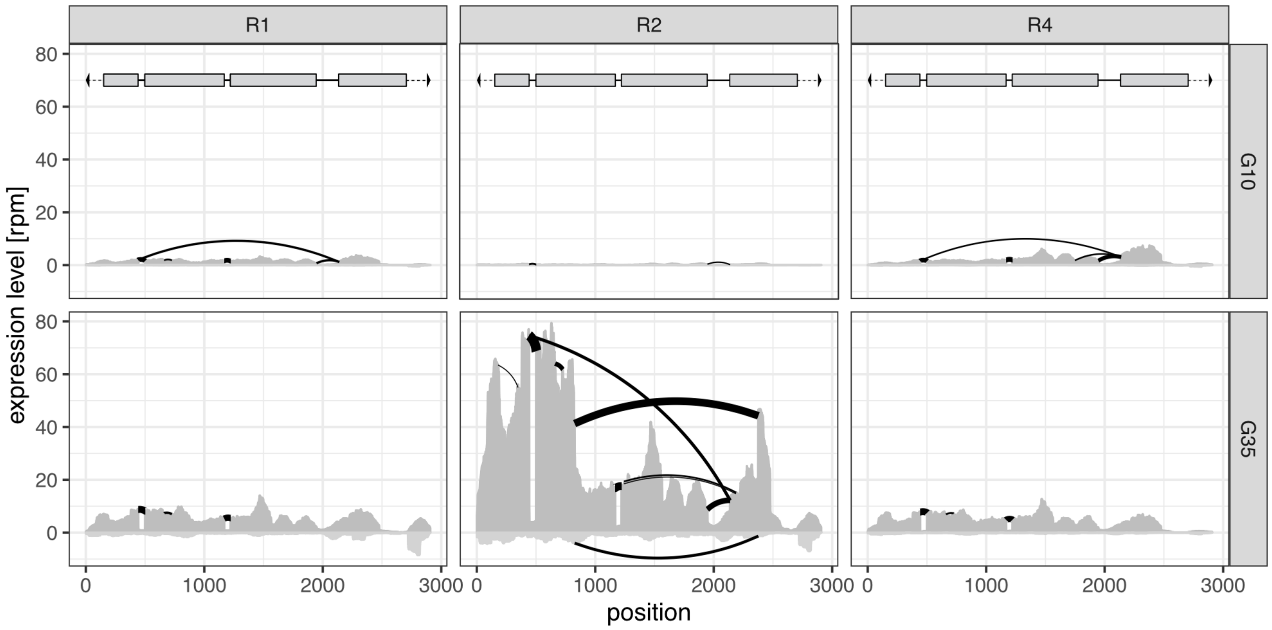

Figure 14: Sashimi plots showing the expression level and splicing of the *P-element* in ovaries. Data are shown for different replicates (top panel) and generations during the invasion (right panel). Both the expression and splicing level (width of the black arcs) were normalized to a million mapped reads. Sense expression and gaps of sense transcripts (splicing or internal deletions) are shown on the positive y-axis whereas antisense expression and gaps of antisense transcripts is shown on the negative y-axis. The structure of the *P-element* is shown at the top, where TIRs (black triangles), the four exons (grey rectangles) and introns (black lines) are shown.
