# Supplementary material for: Experimentally evolving Drosophila erecta populations may fail to establish an effective piRNA-based host defense against invading P-elements
Source: Genome Res. 2024 Mar;34(3):410–25. doi: 10.1101/gr.278706.123 (PMC11067887; doi:10.1101/gr.278706.123)
Supplement: Supplement 15 [file Supplementary_Fig_S15.pdf]

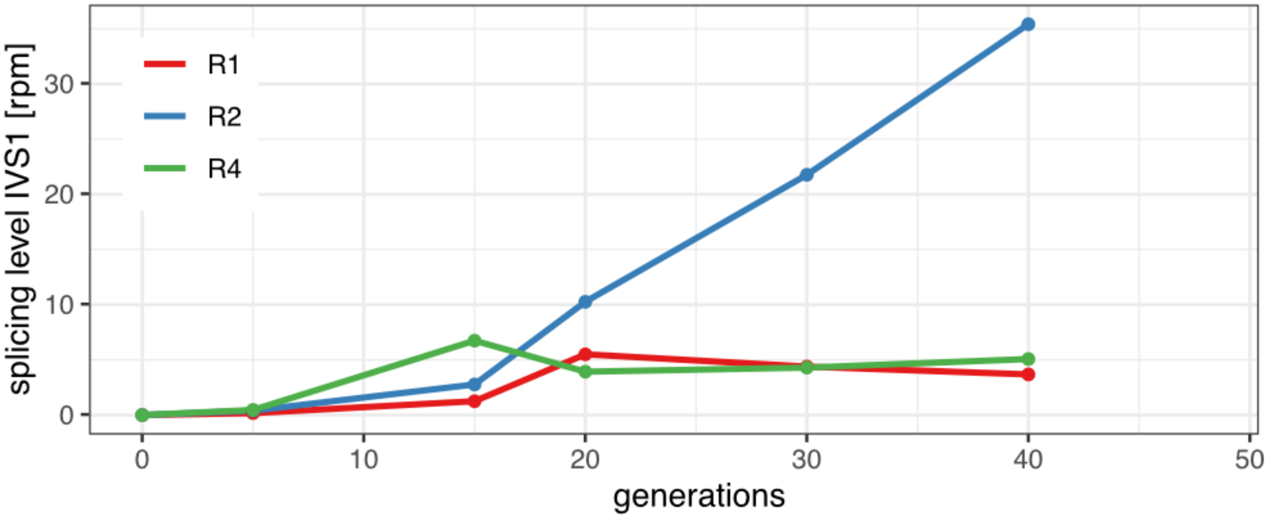

Figure 15: Splicing level of the first intron of the the *P-element* (IVS1) in the experimental populations.
