# Supplementary material for: Experimentally evolving Drosophila erecta populations may fail to establish an effective piRNA-based host defense against invading P-elements
Source: Genome Res. 2024 Mar;34(3):410–25. doi: 10.1101/gr.278706.123 (PMC11067887; doi:10.1101/gr.278706.123)
Supplement: Supplement 17 [file Supplementary_Fig_S17.pdf]

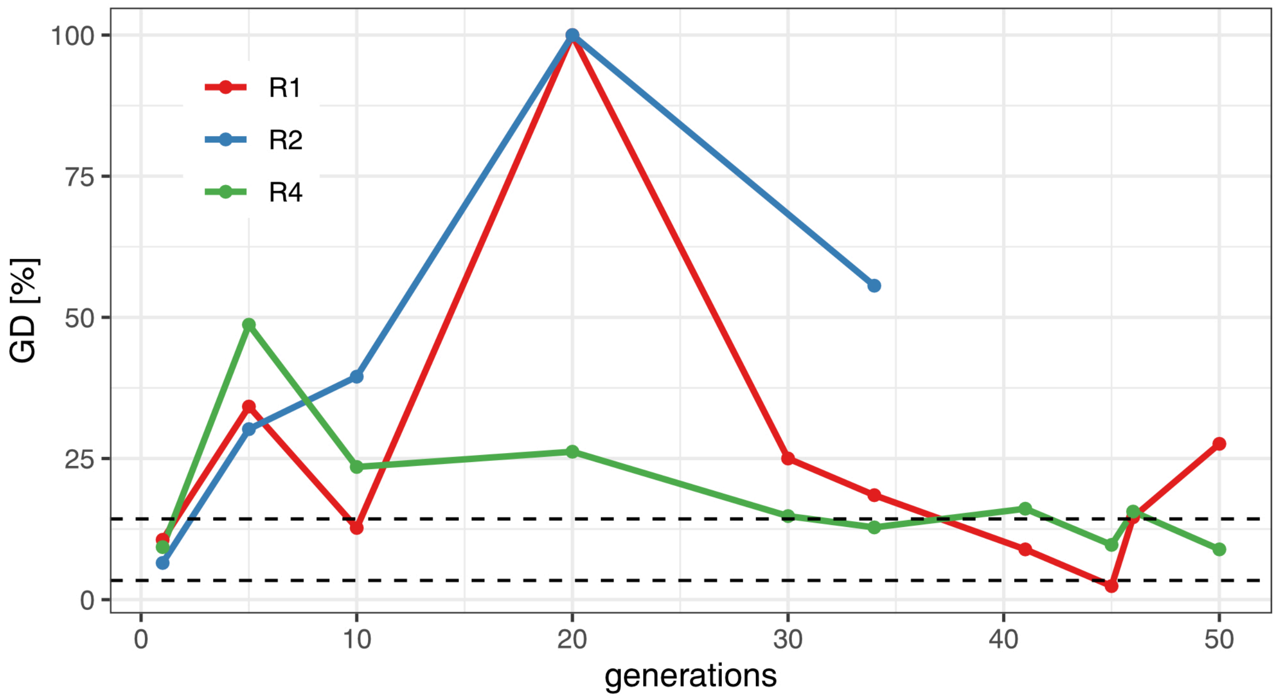

Figure 17: Extend of gonadal dysgenesis (GD) during the experiment. We allowed the experimental populations to lay eggs for 2-3 days, the eggs were reared at 29°C and the percentage of atrophied ovaries was estimated for all three replicates (R1-R4). Dashed lines indicate the range of GD observed for naive flies not having the *P-element*. Note that insufficient numbers of flies eclosed for replicate 2 after generation 34.
