# Supplementary material for: Experimentally evolving Drosophila erecta populations may fail to establish an effective piRNA-based host defense against invading P-elements
Source: Genome Res. 2024 Mar;34(3):410–25. doi: 10.1101/gr.278706.123 (PMC11067887; doi:10.1101/gr.278706.123)
Supplement: Supplement 18 [file Supplementary_Fig_S18.pdf]

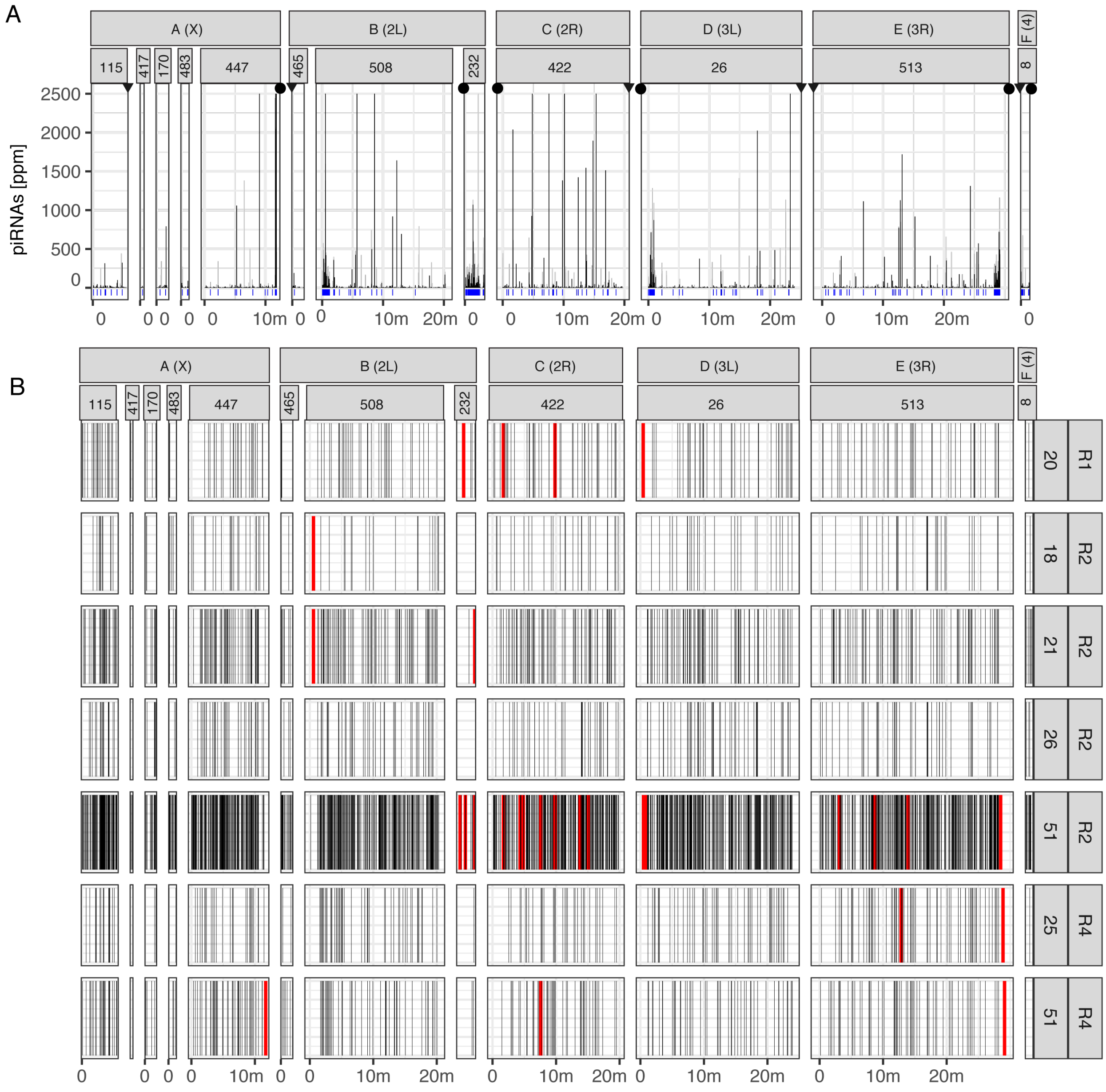

Figure 18: Position of *P-element* insertions in the experimental populations based on long-read data. A) Overview of ambiguously (light grey) and unambiguously (black) mapping piRNA in 1kb windows along the 12 largest contigs of the *D. erecta* assembly. The corresponding Muller element and the likely direction of the telomere (triangle) and the centromere (circle) are shown. At the bottom we show the positions of the annotated piRNA clusters (blue) B) Positions of *P-element* insertions outside (black) and inside of piRNA clusters (red bold) for different replicates and generations (righ panel). Only insertions supported by at least two long reads are shown.
