# Supplementary material for: Experimentally evolving Drosophila erecta populations may fail to establish an effective piRNA-based host defense against invading P-elements
Source: Genome Res. 2024 Mar;34(3):410–25. doi: 10.1101/gr.278706.123 (PMC11067887; doi:10.1101/gr.278706.123)
Supplement: Supplement 19 [file Supplementary_Fig_S19.pdf]

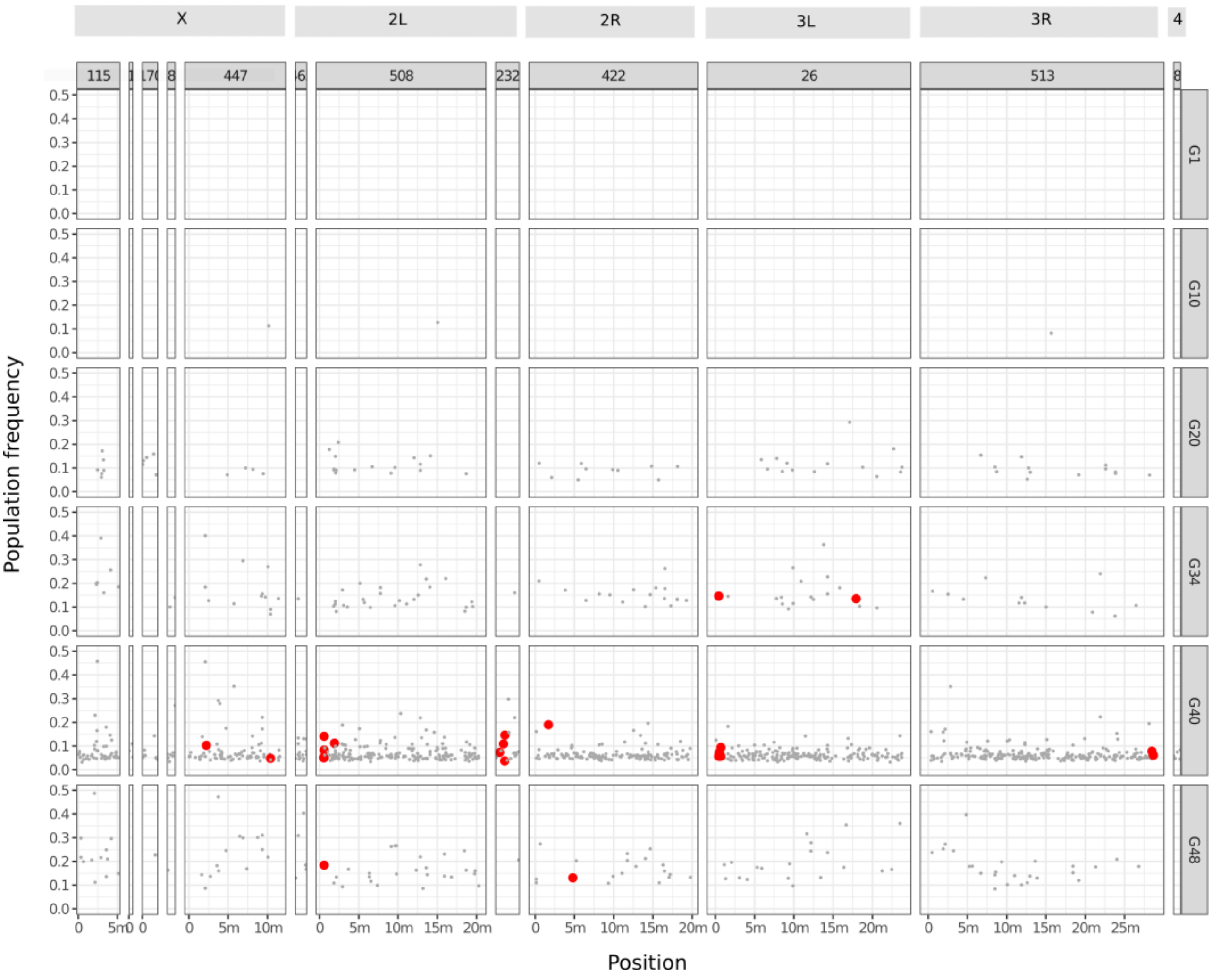

Figure 19: Position and population frequency of *P-element* insertions in replicate 1 based on short-read data. The contigs are in the top panel and the generations in the right panels. *P-element* insertions in piRNA clusters are shown in red. The likely order of *D. erecta* contigs was inferred by aligning the contigs to the *D. melanogaster* genome using Mauve [Darling et al., 2004]. m million base pairs
