# Supplementary material for: Experimentally evolving Drosophila erecta populations may fail to establish an effective piRNA-based host defense against invading P-elements
Source: Genome Res. 2024 Mar;34(3):410–25. doi: 10.1101/gr.278706.123 (PMC11067887; doi:10.1101/gr.278706.123)
Supplement: Supplement 22 [file Supplementary_Fig_S22.pdf]

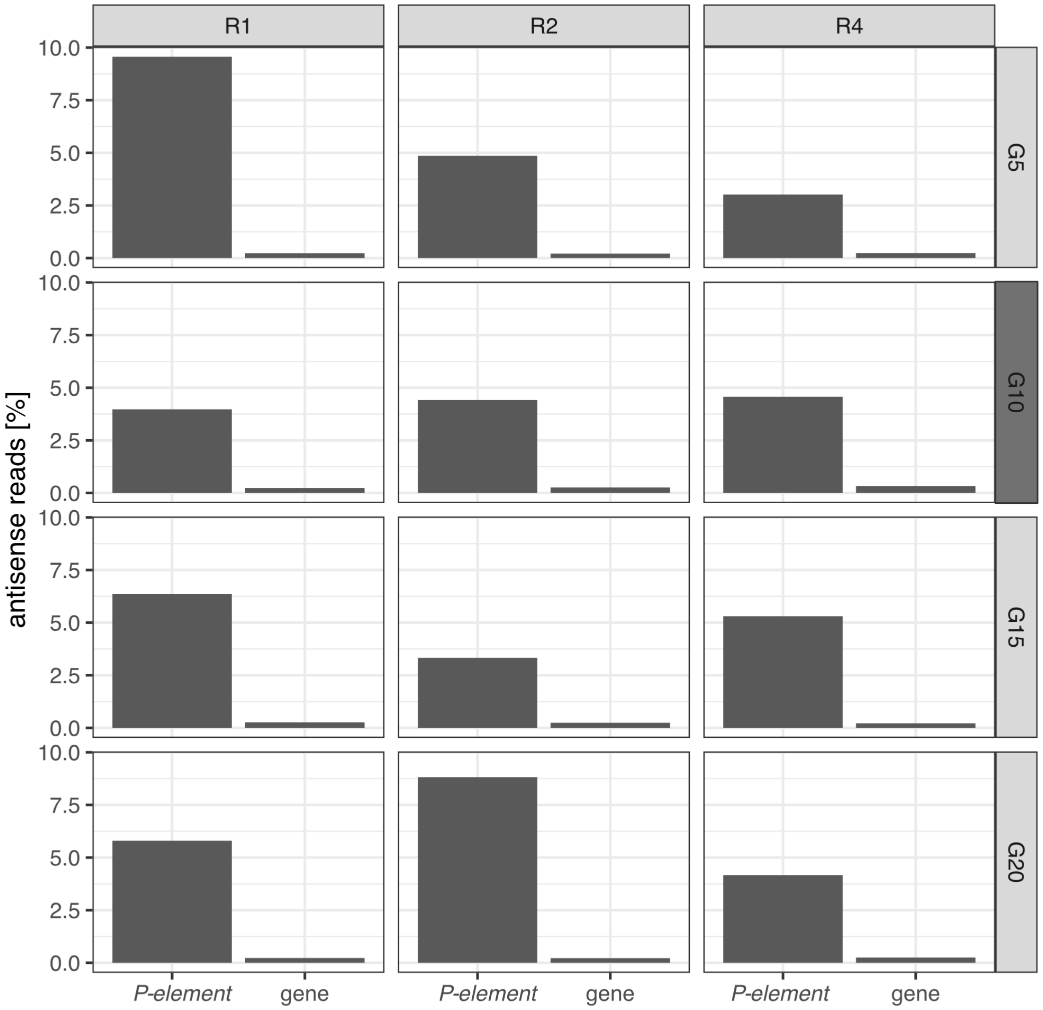

Figure 22: Percentage of antisense reads for the *P-element* and *D. erecta* transcripts at early generations of the invasion ($\leq$ 20 generations). Stranded RNA-seq data were generated for female flies (light grey panels) and ovaries (dark grey panel).
