# Supplementary material for: Experimentally evolving Drosophila erecta populations may fail to establish an effective piRNA-based host defense against invading P-elements
Source: Genome Res. 2024 Mar;34(3):410–25. doi: 10.1101/gr.278706.123 (PMC11067887; doi:10.1101/gr.278706.123)
Supplement: Supplement 23 [file Supplementary_Fig_S23.pdf]

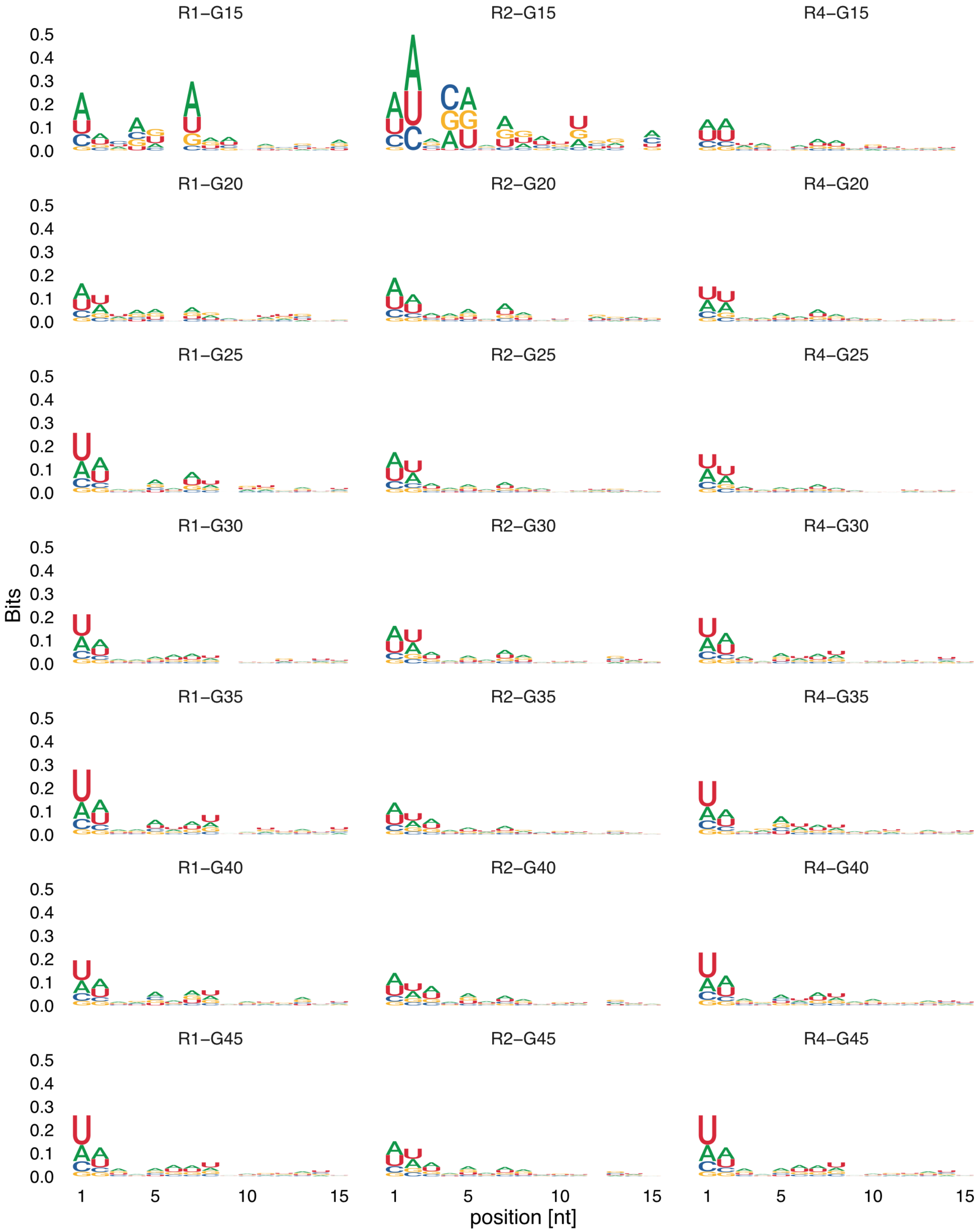

Figure 23: DNA motif of *P-element* siRNAs (20-22nt). Data are shown for three replicates (R1, R2, R4) and multiple generations during the invasion (G15-G45). Prior to generation 15 the abundance of siRNAs was too small for computing motifs.
