# Supplementary material for: Experimentally evolving Drosophila erecta populations may fail to establish an effective piRNA-based host defense against invading P-elements
Source: Genome Res. 2024 Mar;34(3):410–25. doi: 10.1101/gr.278706.123 (PMC11067887; doi:10.1101/gr.278706.123)
Supplement: Supplement 24 [file Supplementary_Fig_S24.pdf]

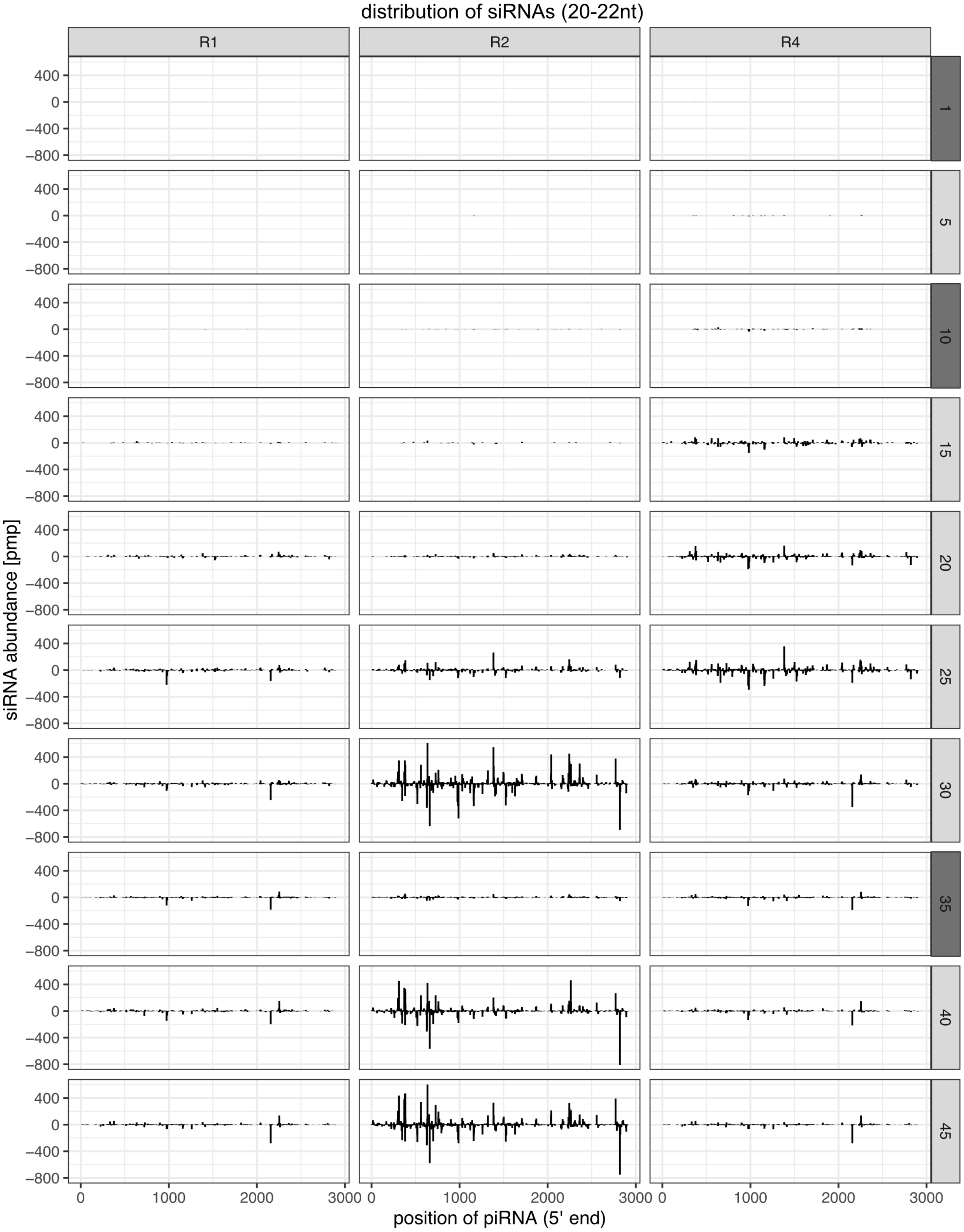

Figure 24: Distribution of siRNAs (20-22nt) along the *P-element*. Only the 5' positions of siRNAs are shown. The abundance of siRNAs is normalized to one million piRNAs (pmp). Replicates are at the top panel and the generations at the right panel. Sense piRNAs are shown on the positive y-axis and antisense piRNAs on the negative y-axis. We extracted small RNAs either from whole bodies of female flies (light grey panels) or from ovaries (dark grey panels).
