# Supplementary material for: Experimentally evolving Drosophila erecta populations may fail to establish an effective piRNA-based host defense against invading P-elements
Source: Genome Res. 2024 Mar;34(3):410–25. doi: 10.1101/gr.278706.123 (PMC11067887; doi:10.1101/gr.278706.123)
Supplement: Supplement 25 [file Supplementary_Fig_S25.pdf]

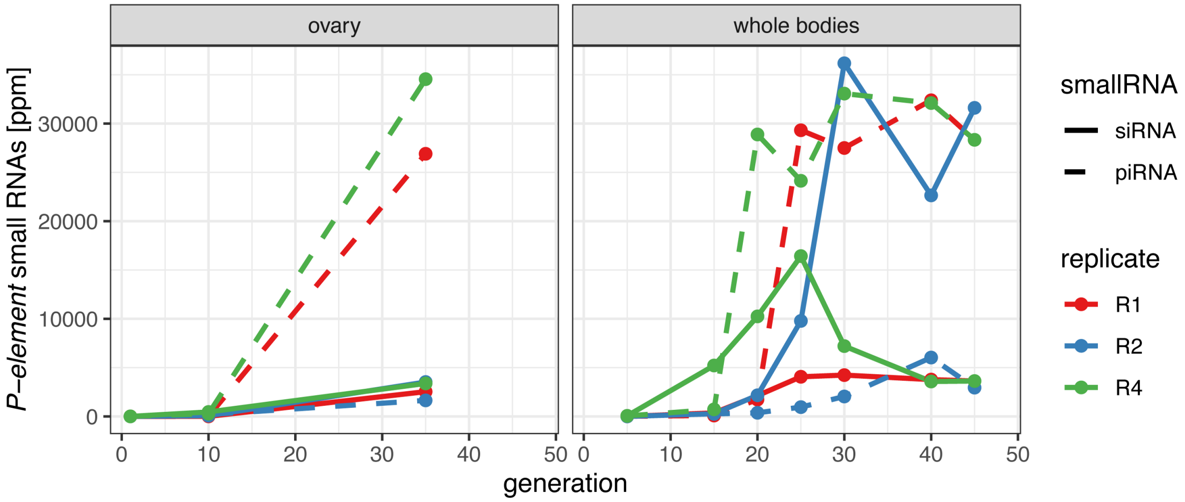

Figure 25: Abundance of siRNAs (20-22nt) during the *P-element* invasion in the three replicates (solid line). The abundance of piRNAs (23-29nt) is shown as reference (dashed lines). RNA was either extracted from whole bodies of female flies or from ovaries.
