# Supplementary material for: Experimentally evolving Drosophila erecta populations may fail to establish an effective piRNA-based host defense against invading P-elements
Source: Genome Res. 2024 Mar;34(3):410–25. doi: 10.1101/gr.278706.123 (PMC11067887; doi:10.1101/gr.278706.123)
Supplement: Supplement 26 [file Supplementary_Fig_S26.pdf]

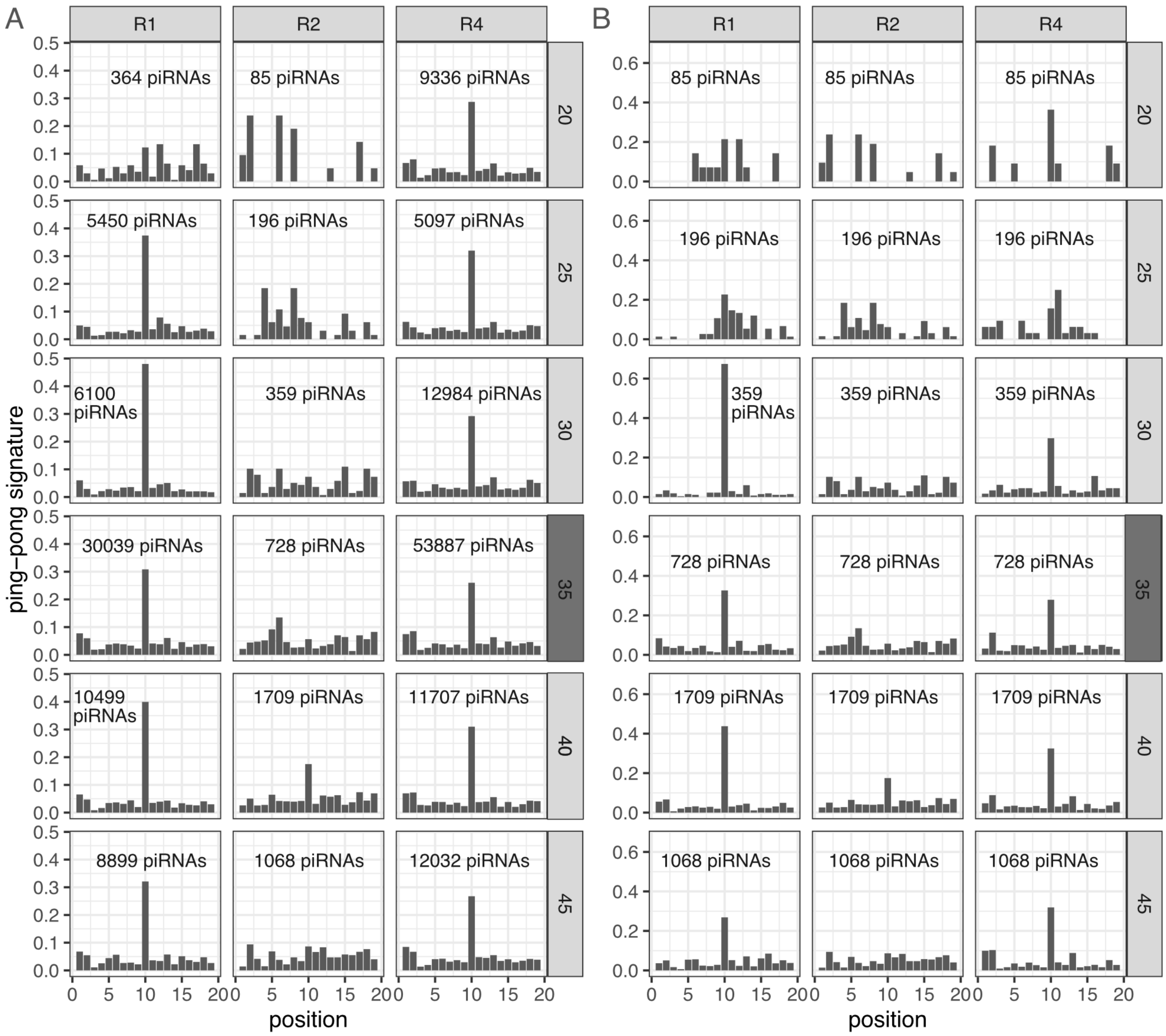

Figure 26: Ping-pong signatures for the *P-element* during the invasion. Data are shown for all piRNAs in a sample (A) and a random subset of the piRNAs (B), where the number of piRNAs within a generation is identical among replicates. The number of piRNAs used for computing the ping-pong signature are indicated in the graph. Ping-pong signatures are computed for different replicates (top panels) and generations during the invasion (right panels). small RNAs were either extracted from whole bodies of female flies (light grey panels) or ovaries (dark grey panels). Due to a low number of piRNAs we could not compute ping-pong signatures for any replicate before generation 20.
