# Supplementary material for: Experimentally evolving Drosophila erecta populations may fail to establish an effective piRNA-based host defense against invading P-elements
Source: Genome Res. 2024 Mar;34(3):410–25. doi: 10.1101/gr.278706.123 (PMC11067887; doi:10.1101/gr.278706.123)
Supplement: Supplement 29 [file Supplementary_Fig_S29.pdf]

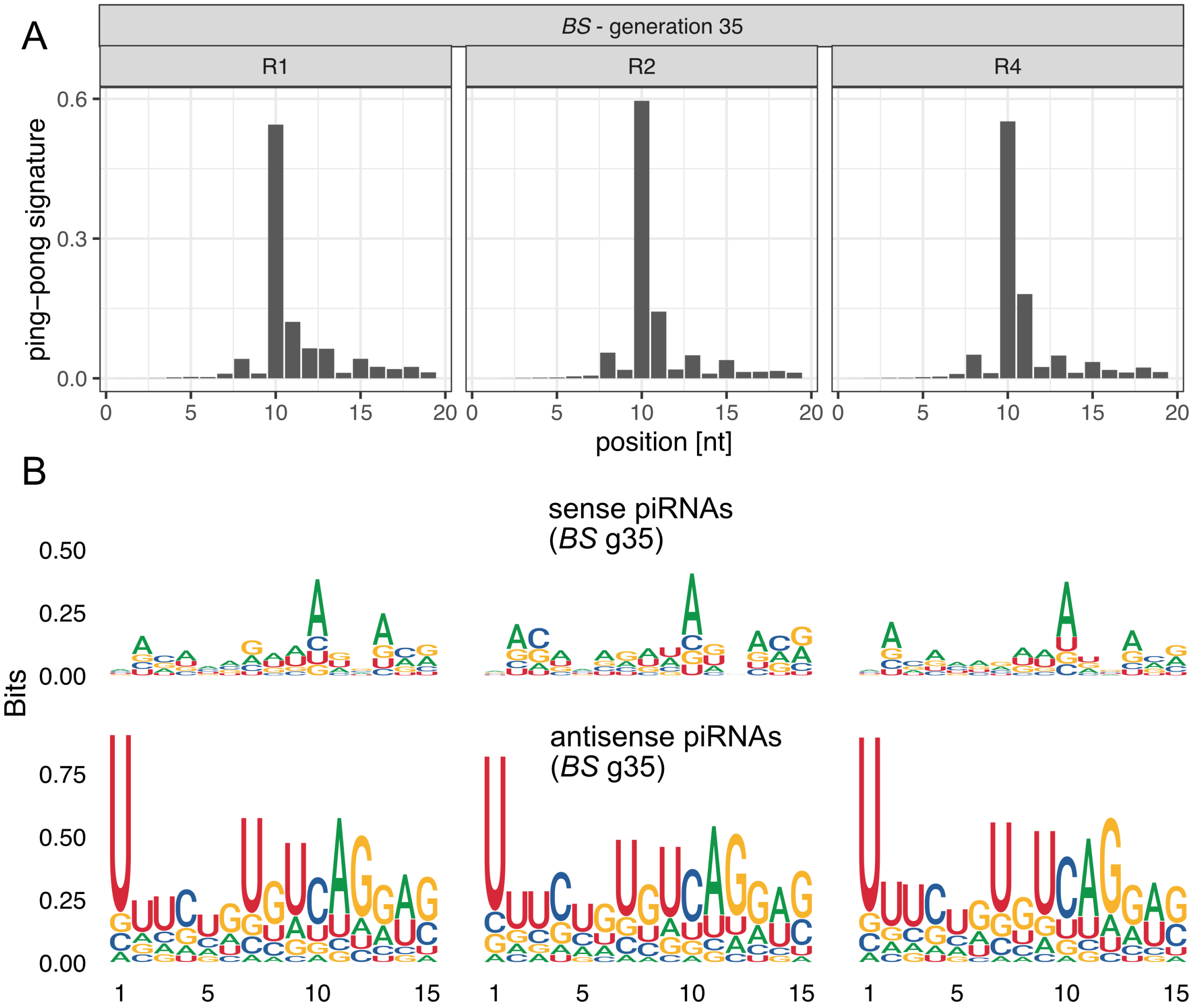

Figure 29: A) The ping-pong signature of BS in ovaries at generation 35. B) Motifs of sense and antisense piRNAs (23-29nt) complementary to BS in ovaries at generation 35.
