# Supplementary material for: Experimentally evolving Drosophila erecta populations may fail to establish an effective piRNA-based host defense against invading P-elements
Source: Genome Res. 2024 Mar;34(3):410–25. doi: 10.1101/gr.278706.123 (PMC11067887; doi:10.1101/gr.278706.123)
Supplement: Supplement 30 [file Supplementary_Fig_S30.pdf]

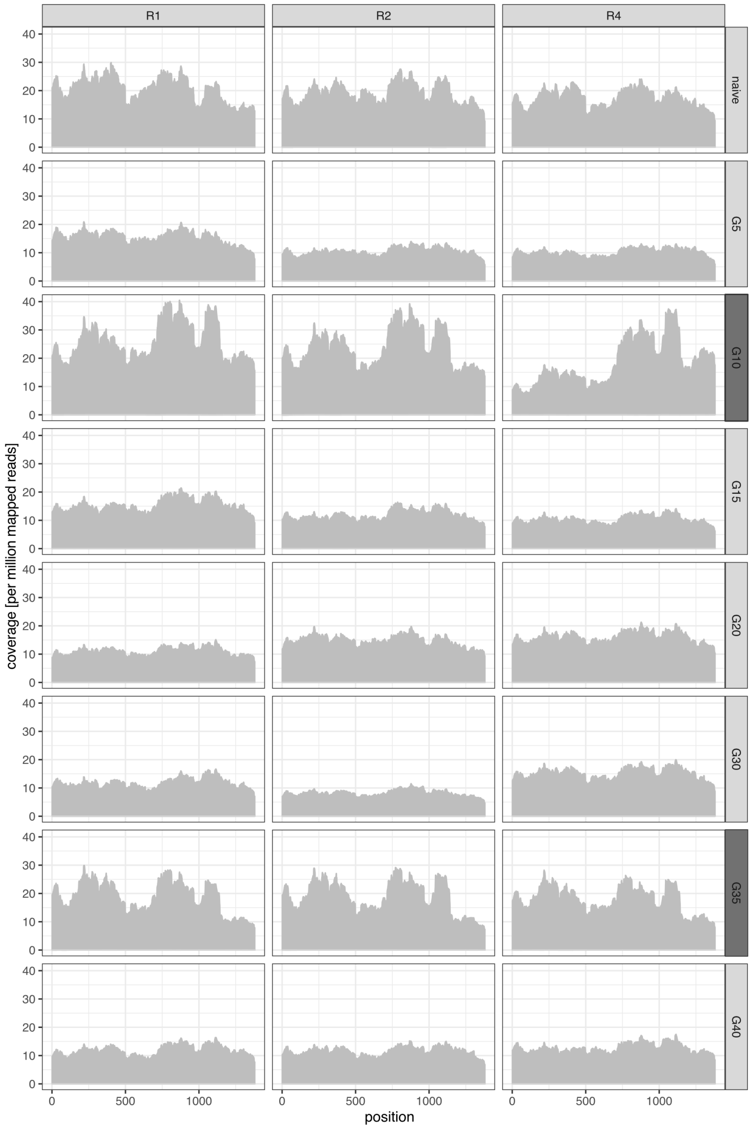

Figure 30: Expression level of *FBtr0141271* (the *D. erecta* ortholog of *lok*) in our experimental populations. Results are shown for all replicates (top panel) at different generations (right panel). RNA extracted from whole flies (light grey panel) or ovaries (dark grey panel) was aligned to *D. erecta* transcripts and the coverage was normalized to a million mapped reads. No significant differences in the average coverage of *FBtr0141271* were found between replicate 2 and replicates 1,4 (Wilcoxon rank sum test $W = 59$, $p = 0.79$).
