# Supplementary material for: Experimentally evolving Drosophila erecta populations may fail to establish an effective piRNA-based host defense against invading P-elements
Source: Genome Res. 2024 Mar;34(3):410–25. doi: 10.1101/gr.278706.123 (PMC11067887; doi:10.1101/gr.278706.123)
Supplement: Supplement 31 [file Supplementary_Fig_S31.pdf]

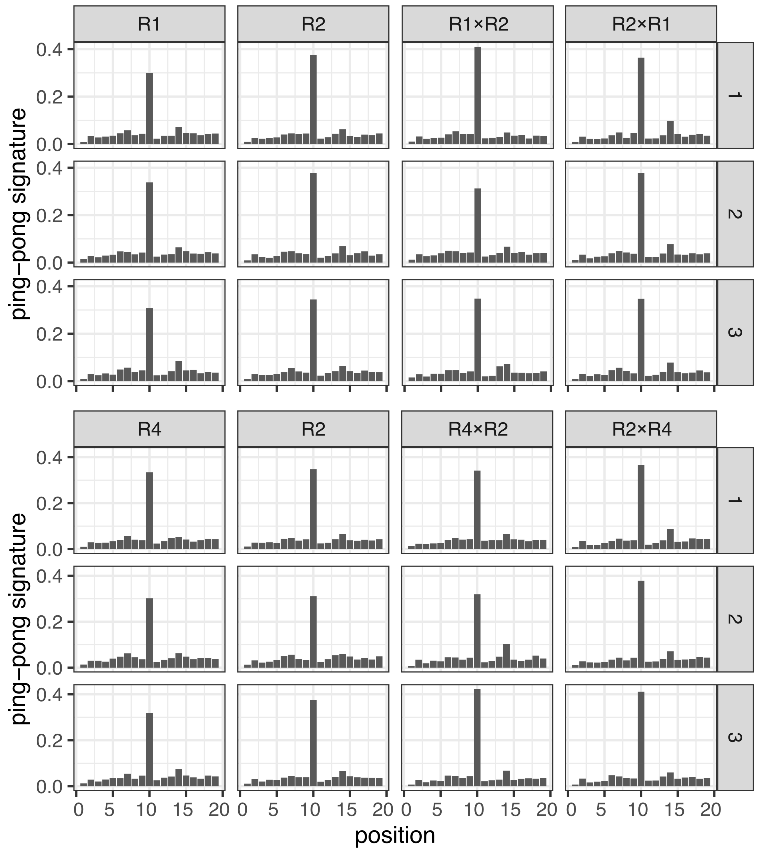

Figure 31: Ping-pong signatures of *Quasimodo* in the parents (R1, R2, R4) and the offspring (R1×R2, R2×R1, R4×R2, R2×R4) of reciprocal crosses among replicates.
