# Supplementary material for: Experimentally evolving Drosophila erecta populations may fail to establish an effective piRNA-based host defense against invading P-elements
Source: Genome Res. 2024 Mar;34(3):410–25. doi: 10.1101/gr.278706.123 (PMC11067887; doi:10.1101/gr.278706.123)
Supplement: Supplement 33 [file Supplementary_Table_S1.pdf]

Table 1: Overview of the Pool-Seq data used in this work. For all replicates (rep) and generations (gen) we show the lane ID, the sequencing technology (platform), the read length (rl.), the inner distance (id.) and the number of sequenced paired-end reads in millions. Samples from naive flies not having the *P-element* are also included.

| rep | gen | lane ID | platform | rl. | id. | reads [M] |
|---|---|---|---|---|---|---|
| naive | - | CEBYBANXX | Illumina HiSeq 2500 | 125 | xx | 13.2 |
| naive | - | CD67CANXX | Illumina HiSeq 2500 | 125 | xx | 19.0 |
| R1 | G1 | CDMKUANXX | Illumina HiSeq 2500 | 125 | 55 | 27.6 |
| R1 | G10 | CDMKUANXX | Illumina HiSeq 2500 | 125 | 52 | 25.9 |
| R1 | G20 | CDMKUANXX | Illumina HiSeq 2500 | 125 | 52 | 28.8 |
| R1 | G34 | CE7UPANXX | Illumina HiSeq 2500 | 125 | 45 | 22.2 |
| R1 | G40 | CEBYBANXX | Illumina HiSeq 2500 | 125 | 60 | 30.1 |
| R1 | G48 | CD67CANXX | Illumina HiSeq 2500 | 125 | 43 | 23.7 |
| R2 | G1 | CDMKUANXX | Illumina HiSeq 2500 | 125 | 44 | 31.7 |
| R2 | G10 | CDMKUANXX | Illumina HiSeq 2500 | 125 | 50 | 25.9 |
| R2 | G20 | CDMKUANXX | Illumina HiSeq 2500 | 125 | 56 | 26.4 |
| R2 | G34 | CE7UPANXX | Illumina HiSeq 2500 | 125 | 39 | 30.8 |
| R2 | G40 | CEBYBANXX | Illumina HiSeq 2500 | 125 | 72 | 25.6 |
| R2 | G48 | CD67CANXX | Illumina HiSeq 2500 | 125 | 34 | 26.9 |
| R4 | G1 | CDMKUANXX | Illumina HiSeq 2500 | 125 | 44 | 27.1 |
| R4 | G10 | CDMKUANXX | Illumina HiSeq 2500 | 125 | 55 | 24.5 |
| R4 | G20 | CDMKUANXX | Illumina HiSeq 2500 | 125 | 57 | 25.8 |
| R4 | G34 | CE7UPANXX | Illumina HiSeq 2500 | 125 | 51 | 23.0 |
| R4 | G40 | CEBYBANXX | Illumina HiSeq 2500 | 125 | 79 | 22.9 |
| R4 | G48 | CD67CANXX | Illumina HiSeq 2500 | 125 | 50 | 18.8 |
