# Supplementary material for: Experimentally evolving Drosophila erecta populations may fail to establish an effective piRNA-based host defense against invading P-elements
Source: Genome Res. 2024 Mar;34(3):410–25. doi: 10.1101/gr.278706.123 (PMC11067887; doi:10.1101/gr.278706.123)
Supplement: Supplement 34 [file Supplementary_Table_S2.pdf]

Table 2: Abundance and effective transposition rate (u') of the *P-element* in the experimentally evolving *D. erecta* populations. Data are shown for different replicates (rep) and generations (gen). The abundance of the *P-element* was estimated as reads per million (rpm) using PoPoolationTE2 [Kofler et al., 2016] and as insertions per haploid genome (ins) using DeviaTE [Weilguny and Kofler, 2019]. t.[M]: total number of reads in million; P: number of reads mapping to the *P-element*.

rep	gen	t.[M]	P	rpm	ins	u'_{rpm}	u'_{ins}
R1	1	27.6	442	8.0	0.4	-	-
R1	10	25.8	2,439	47.2	2.4	0.218	0.224
R1	20	28.8	18,469	320.3	15.5	0.211	0.203
R1	34	22.1	20,510	462.5	21.8	0.027	0.025
R1	40	30	29,032	482.8	22.3	0.007	0.004
R1	48	23.6	25,205	531.9	26.9	0.012	0.023
R2	1	31.6	686	10.8	0.5	-	-
R2	10	25.8	2,292	44.3	2.0	0.169	0.156
R2	20	26.3	17,477	331.6	15.1	0.223	0.224
R2	34	30.7	98,445	1,599.2	92.5	0.119	0.138
R2	40	25.5	100,053	1,954.9	125.8	0.034	0.053
R2	48	26.8	136,585	2,543.3	151.4	0.033	0.023
R4	1	27.1	567	10.4	0.5	-	-
R4	10	24.4	7,340	149.8	7.1	0.344	0.342
R4	20	25.8	28,330	548.1	25.9	0.138	0.139
R4	34	22.9	28,460	619.7	31.7	0.009	0.014
R4	40	22.8	29,645	648.3	30.1	0.008	-0.008
R4	48	18.8	25,880	687.3	37.0	0.007	0.026
