# Supplementary material for: Experimentally evolving Drosophila erecta populations may fail to establish an effective piRNA-based host defense against invading P-elements
Source: Genome Res. 2024 Mar;34(3):410–25. doi: 10.1101/gr.278706.123 (PMC11067887; doi:10.1101/gr.278706.123)
Supplement: Supplement 35 [file Supplementary_Table_S3.pdf]

Table 3: Overview of small RNA data used in this work. For each replicate (r.) we sequenced small RNA at multiple time points (gen.) during the *P-element* invasions and assessed the abundance of reads mapping to TEs (i.e. siRNAs and piRNAs), miRNAs, tRNAs, rRNAs and mRNAs. For each class of small RNAs we also estimated the fraction of reads mapping to the sense strand. Small RNAs were sequenced either from ovaries (tis.: ov) embrios (tis.: em) or whole bodies (tis.: b). Additionally, we sequenced whole bodies and ovaries of female flies not having the *P-element* using three replicates (marked with '-' at r. and gen.). t.[M]: total number of reads in million, m.[M]: mapped reads in million, sr: sub-replicate

					abundance [%]					fraction sense[%]				
r.	gen.	tis.	t. [M]	m.[M]	TE	miRNA	tRNA	rRNA	mRNA	TE	miRNA	tRNA	rRNA	mRNA
R1	1	ov	17.3	8.8	15.5	36.1	1.7	36.9	9.9	6.5	100.0	99.8	99.4	53.9
R1	5	b	11.4	4.9	4.3	55.0	6.2	28.1	6.5	7.7	100.0	99.9	99.8	74.7
R1	10	ov	18.5	8.1	16.5	28.2	6.7	37.4	11.2	6.8	100.0	99.9	99.5	54.8
R1	15	b	10.9	4.7	5.2	67.6	5.1	17.3	4.8	8.0	100.0	99.9	99.6	59.0
R1	20	b	11.5	4.7	4.8	76.2	3.8	12.0	3.3	10.4	100.0	99.9	99.4	53.9
R1	25	b	9.2	3.9	5.1	72.5	2.8	15.9	3.7	9.5	100.0	99.8	99.5	56.6
R1	30	b	15.3	4.4	5.3	66.8	4.1	19.7	4.2	9.0	100.0	99.9	99.6	60.2
R1	35	ov	13.1	6.0	19.7	53.0	2.1	15.3	9.9	8.5	100.0	99.7	97.9	44.0
R1	40	b	11.3	5.0	6.8	79.1	2.9	6.7	4.5	9.2	100.0	99.8	98.2	54.0
R1	45	b	11.5	4.8	7.2	76.4	3.3	8.6	4.5	8.0	100.0	99.9	98.6	51.6
R2	1	ov	13.1	6.3	19.0	46.1	1.8	22.3	10.8	6.6	100.0	99.7	98.9	46.7
R2	5	b	12.7	5.2	5.3	67.8	5.5	15.2	6.2	8.0	100.0	99.9	99.5	69.3
R2	10	ov	10.7	5.5	9.5	23.2	3.5	55.8	8.0	7.1	100.0	99.9	99.8	59.6
R2	15	b	9.9	3.5	3.7	71.3	4.1	16.7	4.3	9.5	100.0	99.9	99.7	66.5
R2	20	b	12.8	5.5	4.4	74.8	4.1	12.9	3.8	10.9	100.0	99.9	99.6	61.3
R2	25	b	12.1	5.2	4.2	73.2	4.2	14.4	4.0	10.7	100.0	99.9	99.6	64.2
R2	30	b	13.0	6.1	3.2	67.2	7.4	18.2	4.1	11.8	100.0	99.9	99.8	73.9
R2	35	ov	5.4	2.5	18.9	57.1	1.5	12.4	10.1	9.2	100.0	99.7	97.5	43.3
R2	40	b	12.8	5.7	5.4	76.1	4.9	9.2	4.4	10.1	100.0	99.9	99.1	60.0
R2	45	b	15.3	7.1	5.6	76.6	4.1	9.5	4.3	9.7	100.0	99.9	98.9	57.2
R4	1	ov	14.3	7.3	13.8	26.7	1.5	47.9	10.1	6.8	100.0	99.8	99.6	57.7
R4	5	b	13.6	5.6	4.8	67.1	6.0	16.9	5.2	7.8	100.0	99.9	99.6	66.8
R4	10	ov	13.9	8.4	2.7	10.0	1.6	82.2	3.5	10.6	100.0	99.9	100.0	75.5
R4	15	b	10.2	4.6	2.0	58.9	4.2	30.5	4.5	11.9	100.0	99.9	99.9	79.4
R4	20	b	10.7	4.7	7.3	75.0	4.5	8.4	4.8	8.2	100.0	99.9	98.7	54.8
R4	25	b	10.5	4.4	5.2	72.9	3.3	14.8	3.8	10.0	100.0	99.8	99.4	54.7
R4	30	b	13.2	6.0	7.0	77.6	3.3	7.9	4.2	8.5	100.0	99.9	98.5	50.5
R4	35	ov	17.8	8.0	20.8	57.4	1.3	10.6	10.1	9.4	100.0	99.5	97.4	42.3
R4	40	b	12.3	6.0	6.4	79.5	3.4	6.3	4.4	9.7	100.0	99.9	98.7	54.9
R4	45	b	14.1	6.6	6.8	78.1	3.6	6.8	4.7	8.2	100.0	99.9	98.4	54.1
-	-	b	9.7	4.3	7.8	77.8	3.1	6.5	4.9	7.6	100.0	99.8	97.8	49.3
-	-	b	12.6	5.8	7.2	77.2	4.1	6.8	4.6	7.9	100.0	99.9	98.1	50.3
-	-	b	30.6	15.1	6.4	69.6	2.9	16.7	4.5	8.0	100.0	99.8	99.4	54.2
-	-	ov	10.9	5.1	21.1	59.9	2.0	6.4	10.7	7.9	100.0	99.7	94.2	40.3
-	-	ov	10.2	4.9	18.7	51.7	1.8	17.7	10.1	7.9	100.0	99.7	97.9	45.2
-	-	ov	9.4	4.2	22.7	57.7	2.3	6.1	11.2	7.7	100.0	99.7	93.9	40.9
	-	em	4.9	2.9	10.9	67.7	1.3	13.3	6.9	4.0	100.0	99.1	99.3	45.2
R1_sr1	G70	b	9.9	4.9	8.6	76.6	5.5	5.8	3.6	8.6	100.0	100.0	99.4	47.3
R1_sr2	G70	b	9.6	4.7	8.4	68.2	5.6	13.8	4.0	8.9	100.0	100.0	99.8	51.6
R1_sr3	G70	b	12.8	6.1	9.2	74.3	5.5	6.7	4.4	9.0	100.0	99.9	99.4	49.5
R1xR2_sr1	F1	b	9.3	4.2	7.8	73.3	6.5	8.8	3.6	10.18	100.0	100.0	99.6	53.2
R1xR2_sr2	F1	b	9.1	4.3	8.7	74.6	6.5	6.8	3.5	9.6	100.0	100.0	99.6	46.5
R1xR2_sr3	F1	b	8.6	4.2	6.8	74.8	7.6	7.6	3.3	10.76	100.0	100.0	99.6	53.6
R2_sr1	G67	b	10.8	5.2	7.2	75.9	5.6	8.0	3.4	10.9	100.0	100.0	99.6	48.4
R2_sr2	G67	b	9.4	4.5	5.9	71.7	6.7	12.5	3.2	12.3	100.0	100.0	99.8	54.2
R2_sr3	G67	b	8.2	4.0	6.3	74.6	7.2	8.5	3.4	10.4	100.0	100.0	99.6	53.5
R2_sr4	G67	b	7.4	3.6	6.4	70.5	9.3	10.0	3.8	9.7	100.0	100.0	99.7	57.8
R2_sr5	G67	b	10.0	5.1	6.5	74.9	7.1	8.1	3.5	11.9	100.0	100.0	99.7	55.4
R2_sr6	G67	b	11.8	5.9	6.8	71.3	7.6	10.4	3.9	11.3	100.0	100.0	99.7	56.6
R2xR1_sr1	F1	b	11.4	6.4	3.6	43.9	17.0	30.0	5.5	11.3	100.0	100.0	99.9	83.4
R2xR1_sr2	F1	b	11.0	6.1	4.9	58.3	21.4	11.0	4.4	11.1	100.0	100.0	99.8	71.6
R2xR1_sr3	F1	b	11.3	6.3	4.3	52.2	27.1	11.6	4.8	11.4	100.0	100.0	99.8	77.2
R2xR4_sr1	F1	b	8.9	4.5	5.0	72.5	9.3	9.9	3.3	10.1	100.0	100.0	99.8	58.7
R2xR4_sr2	F1	b	9.8	4.9	5.7	71.1	9.7	9.8	3.7	10.7	100.0	100.0	99.8	60.7
R2xR4_sr3	F1	b	9.1	4.5	5.6	66.3	10.6	13.7	3.8	10.7	100.0	100.0	99.8	61.2
R4_sr1	G70	b	9.5	4.6	7.8	71.1	7.3	9.9	3.9	8.6	100.0	100.0	99.7	52.4
R4_sr2	G70	b	8.5	3.9	8.8	74.4	6.2	6.7	3.9	8.3	100.0	100.0	99.5	47.6
R4_sr3	G70	b	11.1	5.2	8.5	69.5	7.7	10.0	4.3	7.8	100.0	100.0	99.7	52.9
R4xR2_sr1	F1	b	6.4	2.7	7.7	72.0	9.0	7.7	3.7	11.1	100.0	100.0	99.7	50.8
R4xR2_sr2	F1	b	9.2	4.4	7.2	73.9	7.7	7.7	3.5	10.2	100.0	100.0	99.7	53.2
R4xR2_sr3	F1	b	8.0	4.1	6.4	63.4	10.6	15.8	3.8	10.2	100.0	100.0	99.9	63.4
