# Supplementary material for: Experimentally evolving Drosophila erecta populations may fail to establish an effective piRNA-based host defense against invading P-elements
Source: Genome Res. 2024 Mar;34(3):410–25. doi: 10.1101/gr.278706.123 (PMC11067887; doi:10.1101/gr.278706.123)
Supplement: Supplement 36 [file Supplementary_Table_S4.pdf]

Table 4: Overview of the RNA-Seq data used in this work. RNA was either extracted from ovaries (ov) or whole bodies of female flies (wf). Data are shown for different replicates (rep.) and generations (gen.) of the experimental populations. We also sequenced three replicates of naive flies not having the *P-element*. rl: read length, reads: total number of reads in million (including both reads of the paired ends), m.: mapped reads in million, hq.m.: reads mapped with a high mapping-quality in million (≥ 20). gene m.: reads mapping to a gene in million, $\%se_{gene}$: percentage of 'gene m.' reads aligning to sense strand, pele m.: reads mapping to the *P-element*, $\%se_{pele}$: percentage of 'pele m.' aligning to sense strand

rep.	gen.	tissue	rl	reads	m.	hq.m.	gene m.	$\%se_{gene}$	pele m.	$\%se_{pele}$
-	naive	wf	100	17.07	10.08	9.67	9.65	99.87	1	-
-	naive	wf	100	21.27	13.57	13.02	13.00	99.87	1	-
-	naive	wf	100	22.17	14.50	13.94	13.92	99.88	0	-
R1	5	wf	100	58.72	39.60	37.91	37.84	99.86	82	91.11
R1	10	ov	100	70.74	45.40	41.32	41.23	99.86	1763	96.97
R1	15	wf	100	61.88	44.74	42.81	42.72	99.83	1672	93.93
R1	20	wf	100	72.57	51.42	49.41	49.31	99.85	7062	94.69
R1	30	wf	100	41.03	28.94	27.76	27.70	99.85	3327	87.12
R1	35	ov	100	83.79	52.48	51.00	50.85	99.80	7531	83.94
R1	40	wf	100	56.17	40.18	38.73	38.65	99.84	3849	88.52
R2	5	wf	100	52.82	37.33	35.97	35.90	99.86	326	95.60
R2	10	ov	100	78.33	50.41	46.72	46.61	99.83	291	97.00
R2	15	wf	100	53.05	37.88	36.37	36.29	99.83	2537	96.91
R2	20	wf	100	51.24	34.98	33.70	33.62	99.86	8207	91.17
R2	30	wf	100	71.36	51.35	49.02	48.89	99.83	20539	92.02
R2	35	ov	100	85.42	54.40	52.96	52.80	99.84	39917	93.72
R2	40	wf	100	51.54	36.79	35.37	35.28	99.86	21325	93.65
R4	5	wf	100	57.66	41.14	39.56	39.47	99.83	471	97.31
R4	10	ov	100	73.43	43.94	40.86	40.74	99.78	2559	96.93
R4	15	wf	100	58.40	42.43	40.60	40.52	99.85	6824	94.82
R4	20	wf	100	73.02	49.39	47.52	47.41	99.85	6576	96.04
R4	30	wf	100	46.35	32.79	31.49	31.43	99.87	3743	95.78
R4	35	ov	100	79.75	52.30	51.00	50.89	99.86	6549	94.86
R4	40	wf	100	60.70	43.15	41.74	41.65	99.86	5317	95.29
