# Supplementary material for: Experimentally evolving Drosophila erecta populations may fail to establish an effective piRNA-based host defense against invading P-elements
Source: Genome Res. 2024 Mar;34(3):410–25. doi: 10.1101/gr.278706.123 (PMC11067887; doi:10.1101/gr.278706.123)
Supplement: Supplement 37 [file Supplementary_Table_S5.pdf]

Table 5: Effect of piRNAs on the expression and splicing of the *P-element*. The expression level of the *P-element* (expr. in rpkm), the splicing level of the three introns (IVS1, IVS2, IVS3 in rpm) and the abundance of *P-element* piRNAs (ppm) are shown. Data are shown for all replicates (r.) at different generations (g.). Estimates of TE copy numbers (TEs) for generations 5, 15 and 30 are based on exponential interpolations (based on the transposition rate; see above) between the two closest neighbouring data points. For each independent variable (expr., IVS1, IVS2, IVS3) we generated a linear model where TEs and piRNAs served as explanatory variables [$lm(splicing \sim tes + pirna)$]. TE copy numbers were significantly positively correlated with all four independent variables ($p < 0.001$). piRNAs showed a significant negative correlation with the splicing level of IVS1 ($p = 0.0021$) and IVS3 ($p = 0.0015$) but did not show any significant correlation with the splicing level of IVS2 ($p = 0.34$) nor the expression of the *P-element* ($p = 0.067$). Finally we generated an alternative linear models where the expression of the *P-element* and the piRNAs served as explanatory variables of the splicing level [$lm(splicing \sim tes + pirna)$]. In all three tests (one test for each of IVS1, IVS2 and IVS3) the expression of the *P-element* was significantly positively correlated with the splicing level ($p < 10^-7$). Under this model piRNAs solely had a significant effect (i.e. a negative correlation) on the splicing of IVS3 (IVS1: $p = 0.14$, IVS2: $p = 0.50$, IVS3: $p = 0.0057$).

| r. | g. | piRNAs | TEs | expr. | IVS1 | IVS2 | IVS3 |
|---|---|---|---|---|---|---|---|
| 1 | 5 | 14.8 | 0.89 | 0.83 | 0.18 | 0.08 | 0.00 |
| 1 | 15 | 90.7 | 6.15 | 12.80 | 1.25 | 1.45 | 0.20 |
| 1 | 20 | 1724.7 | 15.46 | 46.60 | 5.50 | 6.34 | 0.66 |
| 1 | 30 | 27500.0 | 19.79 | 39.20 | 4.39 | 4.53 | 0.03 |
| 1 | 40 | 32387.0 | 22.33 | 32.20 | 3.68 | 3.28 | 0.00 |
| 2 | 5 | 49.7 | 0.97 | 3.17 | 0.40 | 0.40 | 0.13 |
| 2 | 15 | 255.5 | 5.51 | 22.60 | 2.77 | 2.77 | 0.13 |
| 2 | 20 | 372.5 | 15.12 | 79.60 | 10.26 | 8.72 | 1.63 |
| 2 | 30 | 2041.8 | 55.08 | 139.00 | 21.77 | 12.87 | 1.27 |
| 2 | 40 | 6031.1 | 125.80 | 202.00 | 35.42 | 16.42 | 3.07 |
| 4 | 5 | 50.8 | 1.62 | 3.96 | 0.46 | 0.51 | 0.02 |
| 4 | 15 | 721.8 | 13.57 | 55.10 | 6.74 | 7.24 | 0.80 |
| 4 | 20 | 28886.0 | 25.91 | 44.60 | 3.93 | 5.37 | 0.20 |
| 4 | 30 | 33070.1 | 29.77 | 38.50 | 4.30 | 5.03 | 0.00 |
| 4 | 40 | 32101.9 | 30.13 | 41.30 | 5.08 | 5.03 | 0.00 |
