# Supplementary material for: Experimentally evolving Drosophila erecta populations may fail to establish an effective piRNA-based host defense against invading P-elements
Source: Genome Res. 2024 Mar;34(3):410–25. doi: 10.1101/gr.278706.123 (PMC11067887; doi:10.1101/gr.278706.123)
Supplement: Supplement 38 [file Supplementary_Table_S6.pdf]

Table 6: Extent of gonadal dysgenesis during the *P-element* invasion. Results are shown for all three replicates (rep) at different generations (gen). Naive flies not having the *P-element* (marked with '-' in rep and gen) were also analyzed. We estimated the number of flies having clearly visible ovarioles (normal), weakly visible ovarioles (weak), and no discernible ovarioles (dysgenic) at 29°C. Note that we did not obtain any viable females for some generations of R2.)

rep	gen	normal	weak	dysgenic	dysgenic [%]
R1	1	28	3	2	10.6
R2	1	28	2	1	6.5
R4	1	65	6	4	9.3
R1	5	73	0	38	34.2
R2	5	30	0	13	30.2
R4	5	37	5	35	48.7
R1	10	61	2	8	12.7
R2	10	35	5	22	39.5
R4	10	26	0	8	23.5
R1	20	0	0	23	100.0
R2	20	0	0	27	100.0
R4	20	15	1	5	26.2
R1	30	24	0	8	25.0
R2	30	-	-	-	-
R4	30	76	3	12	14.8
R1	34	42	4	8	18.5
R2	34	7	2	9	55.6
R4	34	34	0	5	12.8
R1	41	25	1	2	8.9
R2	41	-	-	-	-
R4	41	94	5	16	16.1
R1	45	83	0	2	2.4
R2	45	-	-	-	-
R4	45	60	1	6	9.7
R1	46	67	1	11	14.6
R2	46	-	-	-	-
R4	46	92	0	17	15.6
R1	50	41	2	15	27.6
R2	50	-	-	-	-
R4	50	25	1	2	8.9
-	-	23	0	1	4.2
-	-	18	0	3	14.3
-	-	28	0	1	3.4
