# Supplementary material for: Experimentally evolving Drosophila erecta populations may fail to establish an effective piRNA-based host defense against invading P-elements
Source: Genome Res. 2024 Mar;34(3):410–25. doi: 10.1101/gr.278706.123 (PMC11067887; doi:10.1101/gr.278706.123)
Supplement: Supplement 39 [file Supplementary_Table_S7.pdf]

Table 7: Overview of Oxford Nanopore long-read data used in this work. For each run we show the mean, median and N50 of the read length. Data are shown for different replicates (rep.) and generations (gen.)

rep.	gen.	run	flowcell	output [Gb]	reads [m.]	N50	mean	median
R1	G20	1	R9.4.1	14.66	2.47	12,968	5,917	2,891
R2	G18	1	R9.4.1	10.68	2.04	11,346	5,224	2,723
R2	G21	1	R9.4.1	11.43	2.21	7,566	5,157	4,121
R2	G21	2	R9.4.1	6.88	1.8	5,508	3,724	2,793
R2	G21	3	R9.4.1	2.40	0.36	9,646	6,611	5,087
R2	G26	1	R9.4.1	5.66	0.96	8,773	5,875	4,445
R2	G51	1	R9.4.1	3.65	0.73	9,575	4,954	3,002
R2	G51	2	R9.4.1	25.5	6.55	7,833	3,901	2,205
R4	G25	1	R9.4.1	10.7	1.4	12,239	7,610	4,739
R4	G51	1	R9.4.1	15.9	3.85	8,037	4,137	2,442
