# Supplementary material for: Experimentally evolving Drosophila erecta populations may fail to establish an effective piRNA-based host defense against invading P-elements
Source: Genome Res. 2024 Mar;34(3):410–25. doi: 10.1101/gr.278706.123 (PMC11067887; doi:10.1101/gr.278706.123)
Supplement: Supplement 40 [file Supplementary_Table_S8.pdf]

Table 8: Overview of *P-element* insertions in piRNA clusters that are supported by at least two long-reads at early generations of the experimental populations ($g. \leq 26$). Reads from different Oxford Nanopore libraries (runs) of the same sample were merged. For each insertion we show the contig, the position (*pos*), the maximum number of bases aligning to the *P-element* (*sup*; the length of the *P-element* is 2,907bp) and the orientation of the *P-element* insertion (*ori*). Given the coverage (*cov*) and the number of reads (r) supporting a *P-element* insertion the population frequency of the insertion ($f = r/cov$) can be computed. The coverage was inferred from IGV and the number of reads supporting an insertion was corrected ($+x$ in column r) if a read was missed by our automated approach (for very long reads with central *P-element* insertions, minimap2 may miss supplementary alignments to the *P-element*; these *P-element* insertions are instead reported as long insertions). For 5kb windows around each insertion we also show the expression level of piRNAs (*expr* uniquely mapping piRNAs per million piRNAs), the strand bias (*s.b*: -1 all piRNAs are antisense, 1 all piRNAs are sense, 0 equal amounts of piRNAs are sense and antisense) and the degree of maternal transmission (*m.t*: 0 no piRNAs are found in the embryo, 1 equal amounts of piRNA in ovaries and embryo). Note that the low population frequency (f) of the insertions is not compatible with a model assuming that a fixed cluster insertions is responsible for silencing the *P-element* around generation 20 in replicates 1 and 4 (R1, R4). rep. replicate

rep.	g.	contig	pos	cov	r	f	sup	ori	expr	s.b	m.t
R1	20	contig_232	1,319,408	73	6+2	0.11	2,021	fwd	39.40	0.62	0.51
R1	20	contig_26	428,194	92	2	0.02	2,437	rev	809.94	-0.88	0.06
R1	20	contig_422	1,690,050	203	3	0.01	2,907	fwd	1049.45	0.82	0.61
R1	20	contig_422	9,830,474	95	3	0.03	2,907	fwd	2291.91	-0.21	0.06
R2	18	contig_508	579,832	62	7+2	0.14	2,389	rev	9.11	-0.47	0.84
R2	21	contig_232	3,074,029	180	2	0.01	2,825	fwd	225.86	-0.96	0.02
R2	21	contig_508	579,833	134	5+2	0.05	1,559	rev	9.11	-0.47	0.84
R4	25	contig_513	12,838,058	91	3+1	0.04	1,434	fwd	1128.25	1.00	0.37
R4	25	contig_513	28,883,839	64	3	0.05	2,613	rev	24.30	-0.47	0.24
