# Supplementary material for: Experimentally evolving Drosophila erecta populations may fail to establish an effective piRNA-based host defense against invading P-elements
Source: Genome Res. 2024 Mar;34(3):410–25. doi: 10.1101/gr.278706.123 (PMC11067887; doi:10.1101/gr.278706.123)
Supplement: Supplement 41 [file Supplementary_Table_S9.pdf]

Table 9: Overview of reads supporting *P-element* insertions inside (*obs.ci*) and outside of piRNA clusters (*non.ci*) in our long-read libraries. Based on the shotgun model each diploid individual should carry about four *P-element* insertions in a piRNA cluster, i.e. two insertions per haploid genome [Kofler et al., 2018, Kofler, 2019]. Given that the average coverage (*cov*) approximates the number of haploid genomes present in a sample, the number of cluster insertions expected under the shotgun model can be computed ($exp.ci = 2 \times cov$). Note that, in all samples, the number of *P-element* insertions in piRNA clusters is lower than expected under the shotgun model ($obs.ci/exp.ci < 1$).

rep	gen	run	cov	non.ci	obs.ci	exp.ci	obs./exp
R1	G20	1	90.5	1043	18	181.0	0.10
R2	G18	1	53.2	685	12	106.5	0.11
R2	G21	1	58.5	1394	4	116.9	0.03
R2	G21	2	31.8	854	3	63.6	0.05
R2	G21	3	12.1	450	7	24.2	0.29
R2	G26	1	28.8	1267	6	57.7	0.10
R2	G51	1	12.4	1239	17	24.8	0.69
R2	G51	2	127.2	14758	170	254.4	0.67
R4	G25	1	63.7	1619	8	127.5	0.06
R4	G51	1	70.9	2521	26	141.8	0.18
