# Supplementary material for: Experimentally evolving Drosophila erecta populations may fail to establish an effective piRNA-based host defense against invading P-elements
Source: Genome Res. 2024 Mar;34(3):410–25. doi: 10.1101/gr.278706.123 (PMC11067887; doi:10.1101/gr.278706.123)
Supplement: Supplement 42 [file Supplementary_Table_S10.pdf]

Table 10: Normalized number of reads aligning to *Drosophila* viruses in the small RNA libraries [rpm]. We sequenced small RNAs for each replicate (r.) at multiple generations (gen.). Small RNAs were sequenced either from ovaries (tis.: ov) or whole bodies of female flies (tis.: b). Additionally, we sequenced whole bodies and ovaries of naive flies not having the *P-element* (marked with '-' at r. and gen.). Data are shown for the five most abundant viruses across the libraries. 1: MG969167 *Mauternbach Nudivirus*, 2: MZ852356 *picorn-like virus*, 3: BlastFreeCandidate48, 4: *Gosford Narnavirus*, 5: MF893259 *Teise virus* segment 1). No significant differences in the abundance of any of these viruses was found between replicate 2 and replicates 1+4 (Wilcoxon rank sum tests; $p \geq 0.198$

r.	gen.	tis.	1	2	3	4	5
R1	1	ov	53.32	171.63	1.01	15.15	7.98
R1	5	b	127.44	26.34	47.65	17.72	76.28
R1	10	ov	19.99	9.67	0.96	23.1	18.68
R1	15	b	98.14	5.66	7.85	18.12	29.05
R1	20	b	125.65	0.47	95.27	17.87	11.42
R1	25	b	111.64	2.41	69.14	17.56	17.08
R1	30	b	48.59	18.16	41.72	14.56	10.8
R1	35	ov	100.27	13.27	0.52	42.62	5.16
R1	40	b	24.91	0.21	55.41	25.74	9.72
R1	45	b	29.93	2.45	57.12	14.75	12.23
R2	1	ov	39.71	245.37	0.52	15.16	7.11
R2	5	b	42.1	2.03	47.43	20.94	32.12
R2	10	ov	639.87	11.87	0.42	16.87	60.95
R2	15	b	75.24	6.41	5.64	16.4	27.67
R2	20	b	136.03	3.81	54.22	17.19	14.34
R2	25	b	347.03	2.96	64.39	19.17	16.48
R2	30	b	123.75	3.99	26.39	18.81	25.24
R2	35	ov	103.95	17.88	1.21	36.97	5.76
R2	40	b	178.61	0.46	44.73	19.22	16.77
R2	45	b	61.52	2.22	29.83	20.25	14.59
R4	1	ov	68.07	575.14	0	14.54	6.18
R4	5	b	80.64	0.55	28.03	20.77	33.16
R4	10	ov	293.01	9.49	1.87	12.36	21.99
R4	15	b	187.66	16.69	10.56	12.9	34.68
R4	20	b	59.82	3.31	78.04	20.55	12.18
R4	25	b	122.56	2.01	63.14	17.36	12.99
R4	30	b	69.05	4.89	26.32	20.34	14.35
R4	35	ov	62.97	40.51	1.05	42.86	2.55
R4	40	b	40.57	0.64	89.58	25.75	10.99
R4	45	b	97.99	4.3	44.64	19.24	13.18
-	-	b	41.89	2.4	92.69	22.27	10.11
-	-	b	50.12	1.22	24.52	25.45	10.51
-	-	b	155.05	10.3	47.3	22.66	25.04
-	-	ov	29.87	4.43	0.39	42.47	5.21
-	-	ov	48.22	13.69	0.58	32.37	3.4
-	-	ov	25.75	2.97	0.26	44.51	3.05
