# Supplementary material for: Experimentally evolving Drosophila erecta populations may fail to establish an effective piRNA-based host defense against invading P-elements
Source: Genome Res. 2024 Mar;34(3):410–25. doi: 10.1101/gr.278706.123 (PMC11067887; doi:10.1101/gr.278706.123)
Supplement: Supplement 43 [file Supplementary_Table_S11.pdf]

Table 11: *P-element* polymorphisms in the experimental populations. The coverage (cov) and allele frequency of the minor allele (%) are shown for polymorphisms having a minimum frequency of 5% and a minimum allele count of 2 in at least one generation (G) of the experiment. rep replicate, ma major allele, mi minor allele

				G1		G10		G20		G34		G40		G48	
rep	pos	ma	mi	cov	%	cov	%	cov	%	cov	%	cov	%	cov	%
1	571	G	A	26	7.69	123	0.00	869	0.23	840	0.12	1260	0.00	836	0.00
1	1342	G	T	24	8.33	106	0.00	786	0.00	877	0.00	1159	0.00	1005	0.10
1	1758	T	A	20	10.00	110	0.00	861	0.12	911	0.11	1251	0.00	1065	0.19
2	100	T	G	34	5.88	83	0.00	743	0.67	4919	0.69	4910	0.49	7083	0.37
2	1924	T	A	32	6.25	107	0.00	708	0.00	3274	0.21	3384	0.06	3586	0.03
2	2399	A	G	30	10.00	83	0.00	661	0.00	4706	0.08	5148	0.04	6832	0.00
2	2800	A	T	24	8.33	95	0.00	671	0.30	4692	0.06	5216	0.06	6445	0.02
4	570	C	T	21	9.52	327	0.00	1276	0.16	1213	0.00	1244	0.00	994	0.00
4	1193	A	T	36	5.55	285	0.00	1222	0.00	1222	0.00	1340	0.00	961	0.10
4	1304	C	T	15	13.33	291	0.00	1157	0.09	1145	0.00	1145	0.00	860	0.00
4	1904	A	T	19	10.53	297	0.67	1142	0.18	1166	0.34	1157	0.00	1019	0.00
