# Supplementary material for: Experimentally evolving Drosophila erecta populations may fail to establish an effective piRNA-based host defense against invading P-elements
Source: Genome Res. 2024 Mar;34(3):410–25. doi: 10.1101/gr.278706.123 (PMC11067887; doi:10.1101/gr.278706.123)
Supplement: Supplement 44 [file Supplementary_Text_S1.pdf]

Supplementary discussion

Alternative models for the silencing of an invading TE

Some alternative hypotheses have been proposed that may account for the silencing of an invading TE. In Koalas it was suggested that failed splicing of retroviruses may trigger piRNA production [Yu et al., 2019]. Under this model we expect that the host defense is triggered by sense piRNAs. However, in replicate 2 we found sense piRNAs (even with a 1U bias) but the host defense was not triggered. Furthermore, it is not clear how general this mechanism is, i.e. if this mechanism also applies to invertebrates and to TEs other than retroviruses. In fact, in some organisms, such as *Drosophila*, the causality may actually be reversed. In *Drosophila* it was suggested that piRNAs act by preventing splicing of introns of some TEs [Teixeira et al., 2017]. For these reasons we do not think that failed splicing is responsible for triggering the host response in our experimental populations.

Casier et al. [2019] observed that *BX2*, a famous piRNA cluster in *D. melanogaster*, may due to elevated temperatures (29°C) occasionally switch spontaneously from an OFF-state (non-piRNA producing) into an ON-state (piRNA producing). It is thus feasible that the spontaneous formation of piRNA-producing loci could contribute to the silencing of an invading TE. *BX2* was however the sole genomic locus that spontaneously generated piRNAs, possibly because even in the OFF-state *BX2* already showed high levels of H3K9me3 and Rhi binding, two factors that may be important for the formation of piRNA clusters [Casier et al., 2019]. Furthermore, spontaneous formation of piRNA clusters was not observed at 25°C, i.e the temperature used in our experiments. We therefore do not consider it likely that environmentally induced spontaneous formation of piRNA clusters contributed to the silencing of our *P-element* invasions.

Finally Komarov et al. [2020] suggested that a low amount of piRNAs, for example generated from remnants of previous invasions, could trigger the formation of novel piRNA-producing loci. For the *P-element*, where we could not find any reads resembling the *P-element* in naïve flies and the negligible amount of piRNAs in naïve flies, this mechanism is likely not responsible for triggering the host defense.

References

K. Casier, V. Delmarre, N. Gueguen, C. Hermant, E. Viodé, C. Vaury, S. Ronsseray, E. Brasset, L. Teysset, and A. Boivin. Environmentally-induced epigenetic conversion of a pirna cluster. *Elife*, 8:e39842, 2019.

P. A. Komarov, O. Sokolova, N. Akulenko, E. Brasset, S. Jensen, and A. Kalmykova. Epigenetic requirements for triggering heterochromatinization and Piwi-Interacting RNA production from transgenes in the drosophila germline. *Cells*, 9(4), 2020.

F. K. Teixeira, M. Okuniewska, C. D. Malone, R.-X. Coux, D. C. Rio, and R. Lehmann. piRNA-mediated regulation of transposon alternative splicing in the soma and germ line. *Nature*, 552:268–272, 2017.

T. Yu, B. S. Koppetsch, S. Pagliarani, S. Johnston, N. J. Silverstein, J. Luban, K. Chappell, Z. Weng, and W. E. Theurkauf. The pirna response to retroviral invasion of the koala genome. *Cell*, 179(3):632–643, 2019.
