# Supplementary material for: Experimentally evolving Drosophila erecta populations may fail to establish an effective piRNA-based host defense against invading P-elements
Source: Genome Res. 2024 Mar;34(3):410–25. doi: 10.1101/gr.278706.123 (PMC11067887; doi:10.1101/gr.278706.123)
Supplement: Supplement 45 [file Supplementary_Text_S2.pdf]

Material and Methods

Strains and Transformation

We obtained the reference strain of *D. erecta*, 14021-0224.01, from the UCSD *Drosophila* Species Stock Center (now the National *Drosophila* Species Stock Center, Cornell University). This strain is derived from a wild-type strain, inbred for eight generations [*Drosophila* 12 Genomes Consortium, 2007]. To introduce the *P-element* into *D. erecta*, the plasmid ppi25.1 - which contains a full-length insertion of the canonical *P-element* (kindly provided by E. Kelleher; GenBank:X06779; [O'Hare and Rubin, 1983]) - was injected into early embryos by Rainbow Transgenic Flies Inc (`https://www.rainbowgene.com/`; Camarillo, CA, USA). We established 12 lines by crossing the transformed adults (2 males and 3 females). The lines were screened for the presence of the *P-element* with PCR using four different primer pairs as described previously [Hill et al., 2016]. Out of the 12 lines tested, 7 contained the *P-element*. The transformed lines were maintained at 20°C (the *P-element* has reduced activity at such low temperatures [Moon et al., 2018, Kofler et al., 2018]) for 3 generations before setting up the experimental populations.

Experimental populations

To establish the experimental populations, we crossed five males from five *P-element* lines with 5 naïve virgin females (in total we crossed 25 males with 25 females) and allowed them to mate for 3 days. After mating, we mixed the 50 crossed flies with 200 naïve *D. erecta* flies. The resulting 250 flies constitute the base population. We maintained 3 replicates of the experimental populations for 50 generations at 25°C using non-overlapping generations. Until generation 34, the populations were maintained at a census size of $N = 250$ by counting 50 flies and then generating 5 piles of flies of identical size. The emergence of Covid around generation 34, forced us to reduce the amount of time spent in the laboratory. Hence, after generation 34, populations were maintained by flipping adult flies to a new bottle.

Illumina sequencing of genomic DNA

Flies from each generation were stored in pure EtOH at -80°C. At about each 10th generation DNA was extracted from pools of 60 flies using a high salt extraction protocol [Miller et al., 1988]. Sequencing libraries were generated with about 330ng DNA and the NEBNext Ultra II FS DNA Library Prep Kit in combination with NEBNext Multiplex Oligos (New England Biolabs, Ipswich, MA, USA). We sequenced 2×125bp reads with an Illumina HiSeq 2500 at the VBCF facility. Individual flies at generation 42 were sequenced by BGI using 2×150bbp reads (BGI Tech Solutions, Hong Kong). For each fly between 23 and 39 million reads were obtained.

Abundance and diversity of TE insertions

We estimated the abundance and diversity of the *P-element* with DeviaTE [Weilguny and Kofler, 2019]. The short reads were trimmed to a size of 125bp and aligned with BWA-SW (v0.7.17 [Li and Durbin, 2009]) to the consensus sequences of TEs in *D. melanogaster* (which contains the *P-element* [Quesneville et al., 2005]) and three single copy genes of *D. erecta*: *tj*, *RpL32* and *rhi* (from FlyBase release 2017_05). The abundance of TEs is estimated by normalizing the coverage of TEs to the coverage of the single-copy genes. DeviaTE also provides the position and frequency of internal deletions and SNPs of the *P-element*.

To identify the genomic position and population frequency of *P-element* insertions, we used PoPoolationTE2 (v1.10.04 [Kofler et al., 2016]). We trimmed reads to a size of 75bp at the 3'-end (which increases the inner distance and thus the accuracy of population frequency estimates with PoPoolationTE2) and aligned the reads with BWA-SW (v0.7.17 [Li and Durbin, 2009] as single-ends to a FASTA file consisting of a long-read based assembly of *D. erecta* [Kim et al., 2021] and the sequence of the *P-element* (X06779.1 [O'Hare and Rubin, 1983]). Using PoPoolationTE2 we restored the paired-end information (se2pe), generated a ppileup file (ppileup --map-qual 15), identified signatures of TE insertions (identifySignatures --mode

separate, --min-count 1, --signature-window fix100), determined the strand of the insertions (updateStrand --map-qual 15 --max-disagreement 0.4), estimated the population frequencies of TE signatures (frequency), filtered TE signatures (filterSignatures filterSignatures --min-count 1 --max-otherte-count 1 --min-coverage 10 --max-structvar-count 1) and finally paired TE signatures to obtain a list of *P-element* insertions (pairupSignatures). The number of reads mapping to the *P-element* (rpm) was also estimated with PopoolationTE2 (stat-reads). We estimated the transposition rate as $u = -1 - (n_{k+t}/n_k)^{(1/t)}$, where t is the time (in generations) between two measurements of TE copy numbers n_k and n_{k+t} (either rpm or abundance normalized to single copy genes).

RNA sequencing

We sequenced RNA either from whole female flies or ovaries. To obtain ovaries, we kept flies aged between 7-15 days on sugar-agar supplemented with yeast for two days. Ovaries were dissected on PBS. We used 30 flies for the extractions of all samples except generation 10 where solely 10-15 flies were used. To obtain embryos, flies (<7 days old) were allowed to lay eggs for 30 minutes on apple-juice agar plates with yeast paste. Embryos were transferred to a fine net, washed in cold water and dechorionated with 50% bleach for 2 minutes. Embryos were washed twice with a wash buffer (140mM NaCl, 0.03% Triton X-100) and frozen in liquid nitrogen. We added 200 μl TRIzol®Reagent (Invitrogen, Carlsbad, CA), to all samples, ground them and added another 800 μl TRIzol. After incubation for 10 minutes, samples were vortexed, and 200 μl Chloroform was added. After spinning 500 μl of the upper phase was transferred to new tubes. 550 μl of isopropanol was added and then incubated for 10 minutes. The samples were spun at 4°C. The RNA pellet was cleaned with freshly made 80% EtOH and then dissolved in Nuclease-free water (Solis BioDyne, Estonia). The concentrations of the RNA samples were measured using Qubit (Invitrogen, Carlsbad, CA).

Small RNA and RNA samples were sequenced by Fasteris (`https://www.fasteris.com/en-us/`. After 2S RNA depletion, the small RNA samples were sequenced using Illumina NextSeq 500 with a read length of 75bp and 50bp. The small RNAs from embryos were sequenced using Illumina HiSeq 2500 with a read length of 50bp. The RNA samples were treated with DNase and poly-A selected before they were sequenced on the Illumina NovoSeq machine, with a read length of 2×100bp.

Analysis of small RNA data

Adaptor sequences were removed with cutadapt (v2.6 [Martin, 2011]) and reads with a length between 18 and 35 nt were retained. We aligned small RNA reads to a FASTA file containing the *D. erecta* tRNAs, miRNAs, mRNAs, snRNAs, snoRNAs, rRNAs (r1.3; http://flybase.org/) and the consensus sequences of TEs from *D. melanogaster* [v9.42; plus *Mariner* GenBank: M14653.1 [Quesneville et al., 2005]] using novoalign (v3.09.00; http://www.novocraft.com/ -F STDFQ -o SAM -o FullNW -r RANDOM). The abundance of different small RNAs, the distribution of piRNAs within the *P-element*, the length distribution of the piRNAs and the ping-pong signal were computed using previously described Python scripts [Kofler et al., 2018]. The motifs of small RNAs were computed with a novel script (smallRNA-U-bias.py) and the R-package ggseqlogo [Wagih, 2017].

To identify piRNA clusters, we mapped the small RNA data from naïve ovaries to a long-read assembly of *D. erecta* [Kim et al., 2021] with novoalign (see above). Unambiguously mapped reads with a size between 23 and 29nt were counted within bins of 500bp, normalized to the number of miRNAs (see above) and piRNA clusters were identified with an algorithm based on local scores, as described previously [Kofler et al., 2018, 2022] (–threshold 10). Clusters smaller than 2000bp were ignored. We estimated the abundance of virus-derived reads in the small RNA libraries by aligning the small RNA data to a collection of *Drosophila* viruses maintained by the Obbard-lab (`https://obbard.bio.ed.ac.uk/index.html`; June 22; [Obbard, 2018, Wallace et al., 2021] with novoalign (v3.03.02; -F STDFQ -o SAM -o FullNW -r RANDOM). We quantified the abundance of reads mapping to virus sequences using a custom script (viral-expression.py). Statistical analysis and visualization of the data were performed in R [R Core Team, 2012].

Analysis of expression data

We aligned the RNA data with gsnap (version 2014-10-22; [Wu and Nacu, 2010]; -N 1) to a FASTA file consisting of the transcripts of *D. erecta* (r1.3; FlyBase) and the consensus sequences of TEs in *D. melanogaster* [Quesneville et al., 2005]. The coverage and splicing level of the *P-element* was visualized in R, based on the results of two scripts, which normalized the samples to a million mapped reads (mRNA-coverage-senseantisense.py, mRNA-splicing-senseantisense.py). The raw counts of reads mapping to all transcripts and TEs were estimated with a separate script (mRNA-expression.py). Significant differences in expression levels were identified with edgeR based on the raw counts (v3.38.1; glmQLFit test; [Robinson et al., 2010]). Volcano plots were generated in ggplot2 [Wickham, 2016] based on the results of edgeR. The orthologous sequence of the *D. melanogaster* gene *lok* was identified by aligning *NM_ 001259171.2* to *D. erecta* transcripts with BWA-SW (v0.7.17; [Li and Durbin, 2009]).

Long-read sequencing

We employed Oxford Nanopore long-read sequencing to identify *P-element* insertions in piRNA clusters. We used 60 flies to extract high molecular weight DNA with a phenol-chloroform extraction method [Sambrook et al., 1989]. We used $1\mu g$ DNA to prepare long-read libraries with the Ligation Sequencing kit SQK-LSK109 (Oxford Nanopore Technologies; Oxford). Libraries were run on R9 flow cells for 72 hours.

The long-reads were aligned with minimap2 (v2.10-r761; -x map-ont -Y -c) [Li, 2018] to a FASTA file containing the long-read assembly of *D. erecta* and the sequence of the *P-element* (see above). We identified reads supporting *P-element* insertions (Pele-insertion-finder.py) in the resulting paf-files. This script also resolved duplex-reads - i.e. reads where parts of a genomic region are sequenced twice in a tandem arrangement with one copy being reverse complemented - by truncating the read at the first base of the reverse complemented sequence. Next, we filtered reads for insertions in piRNA clusters (find-lr-bedinsertion.py; using the piRNA clusters identified above). We identified the final set of *P-element* insertions in piRNA clusters by grouping reads supporting a cluster insertion at similar positions (distance of less than 20nt; group-cluster-insertions.py --pos-tol 20).

To estimate the population frequency of different *P-element* variants, we filtered for long-reads supporting either the insertion of a full-length (FL) *P-element* or an *EP-element* (filter-FL-EP.py; for the *EP-element*, the bases 827-2375 are deleted). We linked the identity of the *P-element* insertion inferred from long reads to the population frequency estimates obtained with PoPoolationTE2 (see above). For each *P-element* insertion we used the frequency estimate of the nearest insertions (popfreq-FL-EP.py). Insertions without proximal frequency estimate were ignored (the maximum distance was 20nt).

Crosses among replicates

At generations 67 - 70 we performed reciprocal crosses between flies from replicate 2 with flies from replicates 1 and 4. We crossed 15 virgin females from replicate 2 with males from replicates 1 and 4 and vice versa. For each cross, we set up 3 sub-replicates. The parental females and the F1 females were used for RNA extraction and sequencing.

Gonadal dysgenesis assays

About 150-200 flies from every fifth generation were allowed to lay eggs at 29°C for three days. The progeny was kept at 29°C until the pupal stage and was then moved to 25 °C. Eclosed flies were transferred to apple-juice agar plates with live yeast paste and kept at 25°C for three days. The flies were dissected in 1x PBS solution, and the size of the ovaries was scored using the following classification: clearly visible ovarioles or eggs (clear), ovarioles barely visible, atrophy in one ovary (weak), no ovarioles or eggs could be detected (absent). The percentage of dysgenic ovaries was computed as $100 \times (absent + (weak/2))/(clear + weak + absent)$

References

Drosophila 12 Genomes Consortium. Evolution of genes and genomes on the *Drosophila* phylogeny. *Nature*, 450(7167):203–18, 2007. ISSN 1476-4687.

T. Hill, C. Schlötterer, and A. J. Betancourt. Hybrid Dysgenesis in *Drosophila simulans* Associated with a Rapid Invasion of the P-Element. *PLoS Genetics*, 12(3):1–17, 2016.

B. Y. Kim, J. R. Wang, D. E. Miller, O. Barmina, E. Delaney, A. Thompson, A. A. Comeault, D. Peede, E. R. D'agostino, J. Pelaez, J. M. Aguilar, D. Haji, T. Matsunaga, E. E. Armstrong, M. Zych, Y. Ogawa, M. Stamenković-Radak, M. Jelić, M. S. Veselinović, M. Tanasković, P. Erić, J. J. Gao, T. K. Katoh, M. J. Toda, H. Watabe, M. Watada, J. S. Davis, L. C. Moyle, G. Manoli, E. Bertolini, V. Koštál, R. S. Hawley, A. Takahashi, C. D. Jones, D. K. Price, N. Whiteman, A. Kopp, D. R. Matute, and D. A. Petrov. Highly contiguous assemblies of 101 drosophilid genomes. *eLife*, 10:1–32, 2021.

R. Kofler, D. Gómez-Sánchez, and C. Schlötterer. PoPoolationTE2: Comparative Population Genomics of Transposable Elements Using Pool-Seq. *Molecular Biology and Evolution*, 33(10):2759–2764, 2016.

R. Kofler, K.-A. Senti, V. Nolte, R. Tobler, and C. Schlötterer. Molecular dissection of a natural transposable element invasion. *Genome research*, 28(6):824–835, jun 2018.

R. Kofler, V. Nolte, and C. Schlötterer. The transposition rate has little influence on the plateauing level of the P-element. *Molecular Biology and Evolution*, 39(7):msac141, 2022.

H. Li. Minimap2: pairwise alignment for nucleotide sequences. *Bioinformatics*, 34(18):3094–3100, 2018.

H. Li and R. Durbin. Fast and accurate short read alignment with Burrows–Wheeler transform. *Bioinformatics*, 25(14):1754–1760, 2009.

M. Martin. Cutadapt removes adapter sequences from high-throughput sequencing reads. *EMBnet. journal*, 17(1):pp–10, 2011.

S. A. Miller, D. D. Dykes, and H. F. Polesky. A simple salting out procedure for extracting DNA from human nucleated cells. *Nucleic acids research*, 16(3):1215, 1988.

S. Moon, M. Cassani, Y. A. Lin, L. Wang, K. Dou, and Z. Z. Zhang. A Robust Transposon-Endogenizing Response from Germline Stem Cells. *Developmental Cell*, 47(5):660–671.e3, 2018.

D. J. Obbard. Expansion of the metazoan virosphere: Progress, pitfalls, and prospects. *Current opinion in virology*, 31:17–23, 2018.

K. O'Hare and G. M. Rubin. Structures of P transposable elements and their sites of insertion and excision in the Drosophila melanogaster genome. *Cell*, 34(1):25–35, 1983.

H. Quesneville, C. M. Bergman, O. Andrieu, D. Autard, D. Nouaud, M. Ashburner, and D. Anxolabéhère. Combined evidence annotation of transposable elements in genome sequences. *PLoS computational biology*, 1(2):166–175, 2005.

R Core Team. *R: A Language and Environment for Statistical Computing*. R Foundation for Statistical Computing, Vienna, Austria, 2012. URL `http://www.R-project.org`. ISBN 3-900051-07-0.

M. D. Robinson, D. J. McCarthy, and G. K. Smyth. edgeR: a Bioconductor package for differential expression analysis of digital gene expression data. *Bioinformatics*, 26(1):139–140, 2010.

J. Sambrook, E. F. Fritsch, T. Maniatis, et al. *Molecular cloning: a laboratory manual*. Number Ed. 2. Cold spring harbor laboratory press, 1989.

O. Wagih. ggseqlogo: a versatile R package for drawing sequence logos. *Bioinformatics*, 33(22):3645–3647, 2017.

M. A. Wallace, K. A. Coffman, C. Gilbert, S. Ravindran, G. F. Albery, J. Abbott, E. Argyridou, P. Bellosta, A. J. Betancourt, H. Colinet, K. Eric, A. Glaser-Schmitt, S. Grath, M. Jelic, M. Kankare, I. Kozeretska, V. Loeschcke, C. Montchamp-Moreau, L. Ometto, B. S. Onder, D. J. Orengo, J. Parsch, M. Pascual, A. Patenkovic, E. Puerma, M. G. Ritchie, O. Rota-Stabelli, M. F. Schou, S. V. Serga, M. Stamenkovic-Radak, M. Tanaskovic, M. S. Veselinovic, J. Vieira, C. P. Vieira, M. Kapun, T. Flatt, J. González, F. Staubach, and D. J. Obbard. The discovery, distribution, and diversity of DNA viruses associated with *Drosophila melanogaster* in Europe. *Virus Evolution*, 7(1), 2021.

L. Weilguny and R. Kofler. DeviaTE: Assembly-free analysis and visualization of mobile genetic element composition. *Molecular Ecology Resources*, 19(5):1346–1354, 2019.

H. Wickham. *ggplot2: Elegant Graphics for Data Analysis.* Springer-Verlag New York, 2016. ISBN 978-3-319-24277-4.

T. D. Wu and S. Nacu. Fast and SNP-tolerant detection of complex variants and splicing in short reads. *Bioinformatics*, 26(7):873–881, 2010.
